# Bridged Boranoanthracenes: Precursors for Free Oxoboranes through Aromatization-Driven Oxidative Extrusion

**DOI:** 10.1021/jacs.4c15496

**Published:** 2025-05-12

**Authors:** Stav Deri, Moran Feller, Shibaram Panda, Batya Blank, Mark A. Iron, Yael Diskin-Posner, Liat Avram, Linda J. W. Shimon, Rakesh Mondal, Samer Gnaim

**Affiliations:** a Department of Molecular Chemistry and Materials Science, 34976 Weizmann Institute of Science, Rehovot 7610001, Israel; b Department of Chemical Research Support, 34976 Weizmann Institute of Science, Rehovot 7610001, Israel

## Abstract

We introduce a novel class of boranobornadiene derivatives, termed boranoanthracene, along with an in-depth study of their structures and reactivities. Using these versatile precursors, we propose a fundamentally novel mechanism for generating free oxoborane species. This pathway enables the formation of aminoxoborane species, which are rarely reported in the literature. The proposed mechanism unfolds via the coordination of an *oxygen*-Lewis base (dimethyl sulfoxide) to the boron center, triggering a fragmentation cascade propelled by oxidative aromatization. A detailed experimental analysis, NMR measurements, and DFT calculations provide a strong evidence supporting our findings. We explored three distinct reactivities of these species: first, the insertion of oxoborane species into B–C bonds, representing, to the best of our knowledge, the first example of this reactivity. Second, we demonstrated the [3 + 2] cycloaddition reaction of oxoboranes with nitrones, offering viable access to new boranoheterocycles. Third, we reported the first example of a [5 + 2] cycloaddition between oxoboranes and azomethine imines, leading to the formation of a seven-membered boracycle. The diverse reactivities and facile generation of aminoxoboranes highlight their immense potential as versatile tools in organic chemistry.

## Graphical abstract

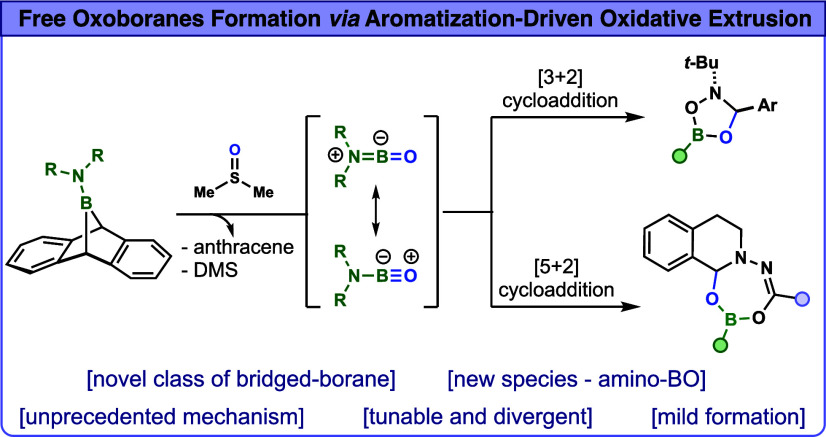

## Introduction

Free oxoboranes are a class of low-coordinate, reactive boron intermediates that have garnered significant interest over the past few decades due to their unique molecular structure and relatively unstudied electronic properties and reactivity. Historically, oxoboranes were initially formed under synthetically impractical conditions, allowing their gas phase characterization under high temperatures pyrolysis conditions (>600 °C) and through photolysis matrix conditions.^1–5^

The first method to generate a free oxoborane under ambient conditions was reported in 1985 by West and co-workers, who synthesized dioxadiboretane **A** (Figure 1A), a dimeric oxoborane.^6^ Through photolysis with irradiation at 254 nm, in one example, they achieved the formation of an arylic oxoborane intermediate. A recent report by Ingleson and co-workers suggests that there are challenges in reproducing the synthesis of dioxadiboretane **A**.^7^ In 1997, Okazaki and co-workers developed a thermal method by utilizing dithiastannaboretane **B** (Figure 1A), which underwent thermolysis in the presence of DMSO to generate an arylic oxoborane species.^8^ However, both methods depend on exotic precursors with highly sterically hindered aryl groups and exhibit low hydrolytic stability, which restricts the synthetic utility of the resulting species. Consequently, research has increasingly shifted toward studying isolable oxoborane species stabilized by Lewis acids and/or Lewis bases. The first example was reported in 2005 by Cowley and colleagues, who demonstrated an isolated three-coordinated oxoborane stabilized by a β-diketiminate substituent and AlCl_3_ as a Lewis acid.^9^ Since then, several stable and isolable oxoboranes, supported by a wide range of ligands and Lewis acids, have been synthesized.^10–17^ Recently, Ingleson and colleagues reported the first isolable base-free, two-coordinate oxoborane with AlCl_3_ and studied its reactivity with phenyl azide.^7^ Moreover, Braunschweig reported the first example of a metal oxoborane complex, BrPt (PCy_3_)_2_-B≡O, as an exotic ligand that is isoelectronic with carbon monoxide.^18,19^ Despite these developments, over the past three decades, progress in generating and studying free oxoborane species has been limited.

**Figure 1:**
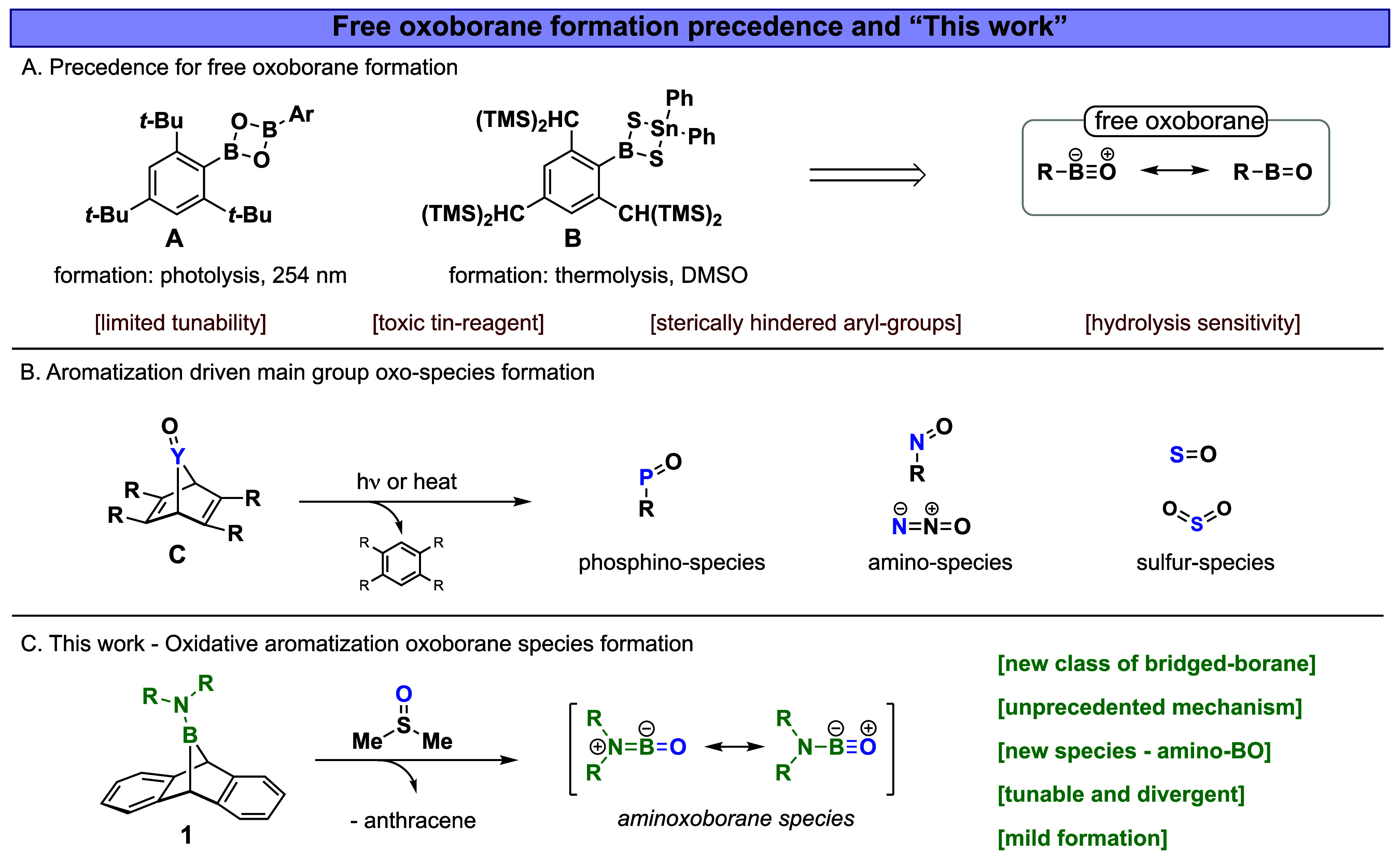
(A) Precedence for free oxoborane formation under solution conditions. (B) Aromatization driven oxo-species formation. (C) This work – Oxidative aromatization aminoxoborane formation.

The aromatization process, which drives the formation of reactive intermediates, is fundamental to organic synthesis.^20–24^ Designing a precursor based on unimolecular fragmentation, AB → À + B̀, relies on selecting an aromatic À to provide the thermodynamic driving force for forming a high-energy species B̀. The energy gain from the aromatization process is crucial for generating such reactive species.^25,26^ One advantage of this chemistry lies in its ability to generate oxygen-containing main group intermediates and species. This ability has been utilized to produce various reactive species from groups 14 to 16, enabling the exploration of their reactivity across different areas of chemistry, as shown in Figure 1B.^27–30^ For instance, Stille and co-workers reported one of the first examples of phosphinidene-oxide generation in solution through the thermal aromatization process of a naphthenelic-based precursor.^31^ Following this seminal work, others have developed analogous precursors, thus allowing them to study their chemical reactivity in synthesis.^32,33^ Similarly, nitrogenic precursors were also employed to generate reactive group 15 intermediates, such as nitrous oxide and nitroso, which are widely applied due to their divergent formation pathways.^34,35^ Interestingly, sulfur bridges have been successfully reported and used by Nakayama and co-workers to investigate the stereochemical reactivity of triplet- and singlet-state sulfur monoxide in cycloaddition reactions.^36^ Although various main group species were formed and studied using the aromatization process, the aromatization-driven generation of reactive boron species with bridged borane heterocycles is still in its infancy. Several attempts by Eisch, Braunschweig, and others to form reactive boron species were unsuccessful since, instead, the electron-deficient nature of boron promoted energetically favorable rearrangement pathways.^37–39^ Cummins et al. recently reported an example of carbene-stabilized haloborylene formation through the aromatization of hexamethylbenzene.^40^

Herein, we introduce a novel class of compounds, boranoanthracenes (**1**), that enable the generation of a previously underexplored class of oxoborane species, aminoxoboranes (Figure 1C). This was achieved via an unprecedented oxidative aromatization mechanism, utilizing DMSO as a mild terminal oxidant. This process facilitates the formation and study of aminoxoboranes under mild thermal conditions, ranging from room temperature to 70 °C. These conditions have enabled the investigation of four electronically and sterically modified aminoxoboranes, in particular, focusing on their unprecedented reactivity in activating and inserting into B–C bonds. Furthermore, we trapped the transient oxoborane species through a novel [3 + 2] cycloaddition reaction with nitrones and [5 + 2] cycloaddition with azomethine imine, resulting in the formation of new borano-heterocycle structures. Through extensive DFT computations, NMR measurements, and experimental analysis, we proposed that the reaction utilizes a biradical stepwise aromatization mechanism initiated by coordinating the *oxygen*-L-type ligand to the bridging borane.

## Results and Discussion

We were intrigued to find that uncoordinated boranobornadiene derivatives **1** (Figure 2A) can be synthesized directly by reacting magnesium-anthracene with various sterically and electronically modified amino borane-dihalides (e.g., chloride, bromide) **2a**–**d** in etheric or aromatic solvents (e.g., THF, DME, or benzene) at room temperature. The reaction mixture was filtered upon consumption of the bright orange magnesium-anthracene complex, all volatile materials were removed *in vacuo*, and the residue was extracted with *n*-pentane or diethyl ether, revealing the bridged boranoanthracenes (BA) **1a**–**d** in 50–75% yields, in gram-scale reactions (Figure 2B). The chemical structures of **1a**–**d** were confirmed using ^1^H-, ^13^C-, and ^11^B-NMR, high-resolution MS, as well as X-ray analysis (Tables S1–S3). Compelling evidence for the formation of bridged boranoanthracenes **1a**–**d** was provided by the appearance of a characteristic ^1^H NMR (benzene-*d*_6_) singlet in the range of 3.77–3.93 ppm for the benzylic protons, ^13^C NMR (benzene-*d*_6_) signals in the range of 41.45–45.74 ppm for the benzylic carbons, and broad ^11^B-NMR (benzene-*d*_6_) signals in the range of 41.33–46.10 ppm for the bridged boron atoms, which shifted downfield compared with that of the starting boron-based materials, amino borane-dihalides **2a**–**d** (sharp ^11^B-NMR signals ranging from 25.0 to 36.3 ppm). Maintaining anhydrous conditions in the reaction system is crucial since conducting the reaction under nonanhydrous conditions revealed the formation of small amounts of side products, including diborane-dihydroanthracene oligomers (Figure S1, Supporting Information).

**Figure 2:**
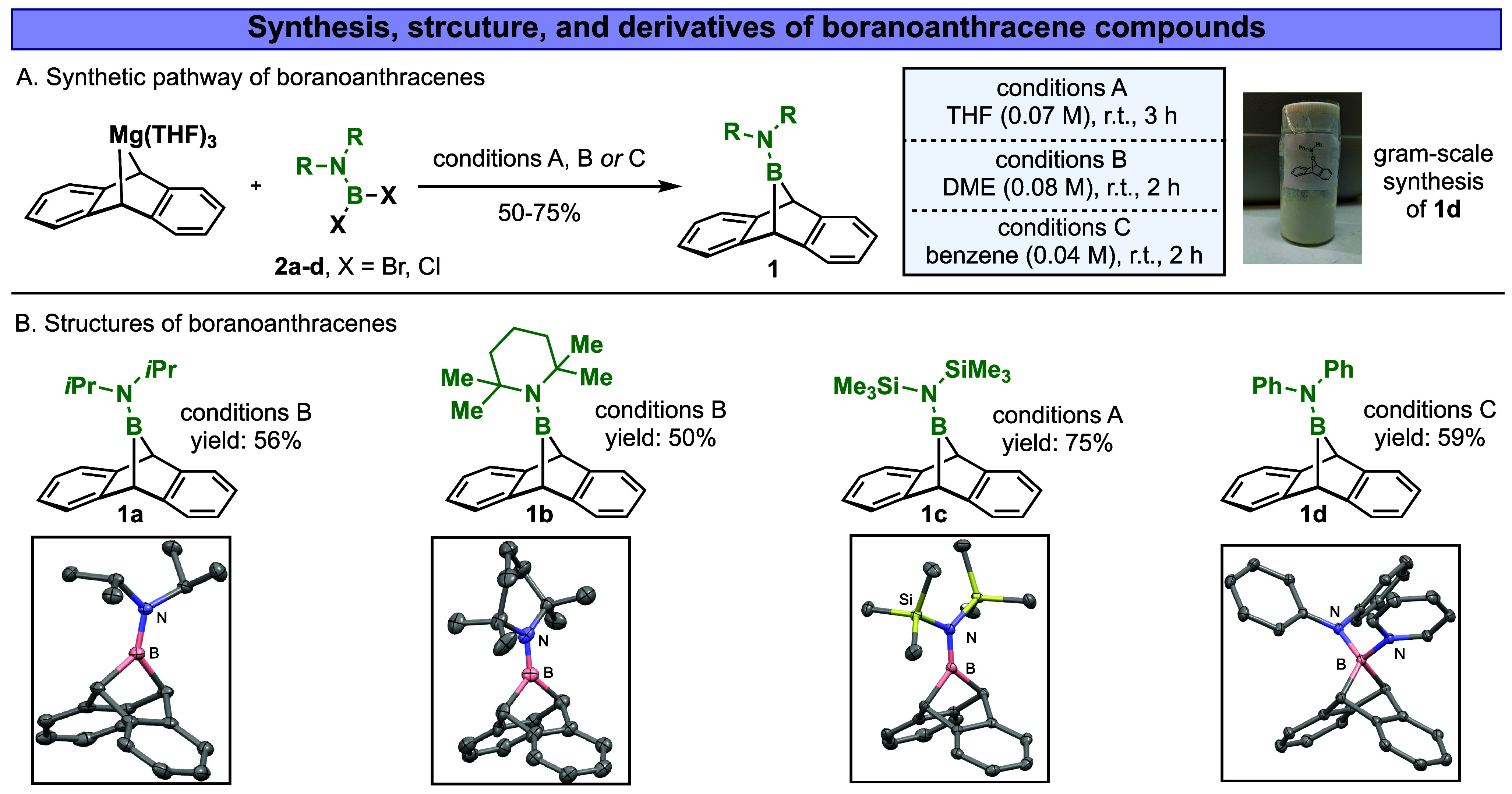
(A) Synthesis of bridged boranoanthracenes **1a**–**d** from magnesium-anthracenes and substituted amino borane-dihalides. (B) X-ray structures of the boranoanthracenes of **1a**–**1c** and the X-ray structure of the pyridine adduct of **1d**. (DME = 1,2-dimethoxy ethane, THF = tetrahydrofuran, Ph = phenyl, SiMe_3_ = trimethylsilyl, *i*Pr = *iso*-propyl).

High-quality single crystals of three bridged boranoanthracenes (**1a**, **1b**, and **1c**), suitable for X-ray diffraction analysis, were obtained by recrystallizing their saturated *n*-pentane or dichloromethane solutions at room temperature or at −35 °C. For boranoanthracene **1d**, an X-ray structure could not be obtained directly; however, its structure was confirmed through X-ray analysis of its pyridine-ligated adduct (Figure 2B, **1d**). Consistent with Piers’ observations,^41^ the solid-state structures of **1** exhibit a pronounced tilt of the trigonal planar boron center toward one of the benzo groups. The acute C_9_–B-C_10_ angle within the boranoanthracenes measures 93.98° for **1a**, 93.49° for **1b**, and 93.21° for **1c**. These angles correlate with increased steric crowding around the boron center, consequently influencing the C_9_–B–C_10_ angle’s width. The angles between the aromatic ring planes (C–C_9_–C or C–C_10_–C) range from 107.02° to 108.52°. Consistent with computational predictions,^42^ the plane defined by boron and the two bridgehead carbon atoms is inclined approximately 6° away from the benzo ring on the same side as the R_2_N substituent on boron. The B_1_–N_1_ interatomic distances are 1.374 Å (**1a**), 1.379 Å (**1b**), and 1.395 Å (**1c**), aligning with the predicted length for a B=N double bond (1.39 Å) and significantly shorter than that of a B–N single bond (1.49 Å). In contrast, the pyridine adduct of **1d** shows a B_1_–N_1_ distance of 1.529 Å, indicative of a single bond character due to boron quaternization. Interestingly, boranoanthracene **1c** exhibits the longest B_1_–N_1_ bond length among the series, likely due to the silyl substituents on nitrogen, which enhance the nitrogen’s *2s* orbital involvement in N–Si interactions, as previously shown with other N–Si systems.^43^

Following the proposed chemical pathway outlined in Figure 1C, we evaluated the behavior and stability of boranoanthracenes (BA) **1a–d** with various mild oxygen-based Lewis bases. All reactions were conducted using benzene as an inert solvent at 70 °C. Whereas alkyl-nitro, phosphine oxide, and various oxygen-containing Lewis bases resulted in only a minor anthracene formation and mainly the recovery of the starting material, DMSO achieved full conversion of the boranoanthracenes through an aromatization process, leading to the complete formation of the expected anthracene. Notably, a control experiment without DMSO, conducted at 70 °C for 24 h, showed full recovery of the BA compounds (Figures S2 and S3). When **1a** was heated at 70 °C in the presence of 20 equiv of DMSO, the boron bridge underwent oxidative thermal extrusion of anthracene, with complete consumption of **1a** within 7 h, as tracked by ^1^H- and ^11^B-NMR analysis (Figures S4–S6). Notably, anthracene was formed during the aromatization process in 84% yield, as measured by ^1^H NMR, referenced to dioxane as an internal standard. The ^11^B-NMR analysis indicates the disappearance of the boranoanthracene **1a** signal at 41.5 ppm and the emergence of a broad boron peak at 22.3 ppm, which corresponds to the trimeric boroxine product (Figure S4).^44–46^ This change indicates the formation of the highly reactive aminoxoborane intermediate **1a’** (Figure 3), which subsequently undergoes rapid cyclization. This result proposes that the aminoxoborane intermediate is formed through the formal oxidative aromatization of boranoanthracene, with DMSO acting as a noninnocent Lewis base and terminal oxidant. This conclusion is further supported by detecting dimethylsulfide (DMS) formation with a 75% yield, as monitored by ^1^H NMR analysis (1.78 ppm; see the mechanistic part for a detailed discussion). Building on these promising results, we examined the behavior of other electronically and sterically modified boranoanthracenes (**1b**–**1d**) under the same reaction conditions. In all instances, the boranoanthracenes underwent similar fragmentation through the proposed reaction pathway, albeit at different reaction rates. TMP-BA **1b** displayed a significantly longer fragmentation time, taking over 10 days to complete the reaction. TMS-BA **1c** and Ph-BA **1d** were fully consumed to produce anthracene and oxoborane species much faster than *i*Pr-BA **1a**, with reaction times of 3 h and 10 min, respectively (Figures S8–S11). Interestingly, the aminoxoboranes behaved differently via an unprecedented reactivity of insertion into B–C bonds to form products **3c** and **3d** (Figure 3). Such products were obtained in decent yields of 60% (**3d**) and 34% (**3c**). Regarding **1c**, running the reaction at room temperature for 48 h improved the insertion reactivity to obtain **3c** in 86% yield. Although the intramolecular insertion reaction of the oxoborane into a C–H bond was reported by Paetzold at high temperatures (600 °C) conditions^3^ (other hydrogen extrusion pathways could be proposed), our result is the first example of oxoborane species engaging in an intermolecular insertion reaction into C–B bonds. Such a result points toward further oxoborane reactivity pathways that remain to be discovered. Finally, it is worth noting that iminoborane intermediacy was not observed under these conditions.

**Figure 3:**
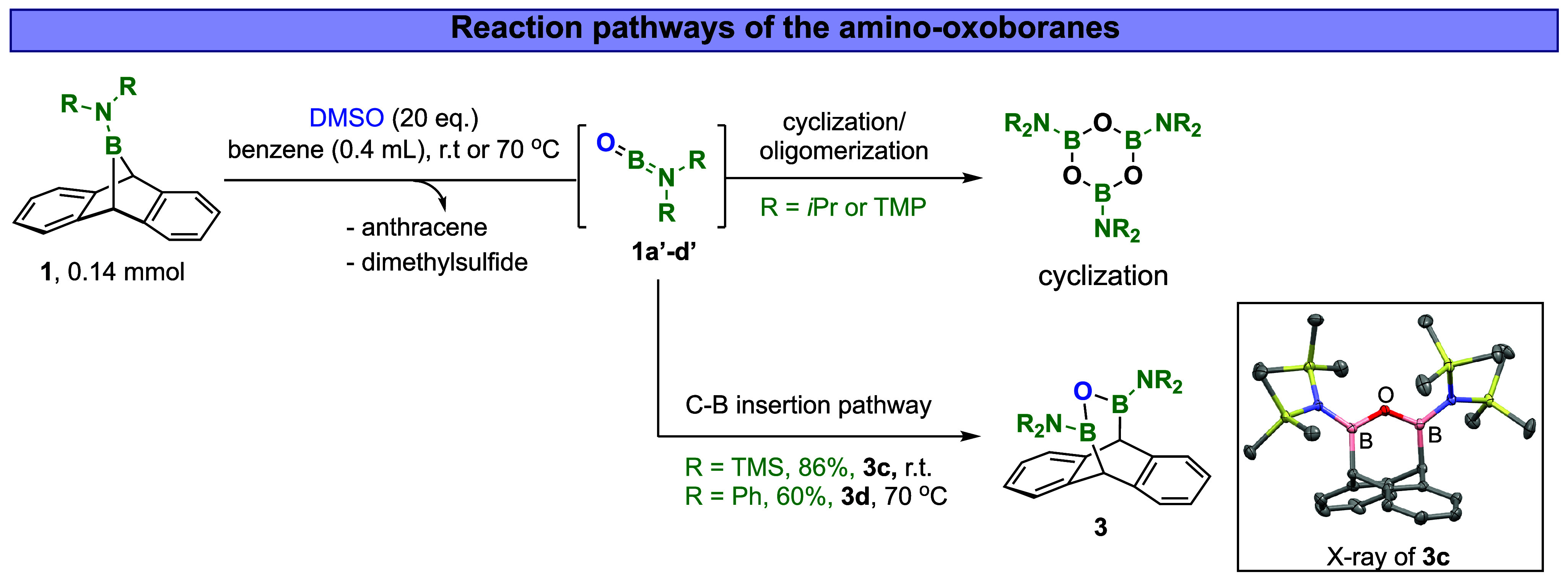
Formation of aminoxoborane species using DMSO and its reaction pathways in cyclization and C–B bond insertion.

We assume that the differences in the oxoborane formation rates are due to variations in the electronic and steric factors of the amine substituents. Regarding **1c** and **1d**, the faster fragmentation compared with **1a** can be attributed to the increased Lewis acidity of the borane, which facilitates improved coordination of DMSO (see the mechanistic part). This effect presumably arises from the weaker π-back-donation of the amine’s lone-pair to the vacant *p*-orbital of boron due to its lower availability. This aligns with the findings of Caputo and Paul, who demonstrated that the amine substituent can directly influence the Lewis acidity of boron in aminoboranes.^47,48^ Conversely, although the electronic characteristics of **1b** are presumably similar to those of **1a**, the prolonged reaction time of **1b** may be explained by the sterically crowded environment of TMP, which hinders DMSO coordination. Indeed, further study in our group is ongoing to assess how steric and electronic factors affect DMSO coordination efficiency.

In order to intercept the proposed transient aminoxoborane intermediate and compete with its cyclization or insertion pathways, the oxidative thermolysis of BA derivatives **1a**–**c** was carried out in benzene and DMSO with stoichiometric amounts of arylnitrones (such as **4**, 1 equiv, Figure 4A). The use of nitrones was motivated by the oxophilic nature of oxoborane, which facilitates 1,3-dipolar cycloaddition. When a solution of TMS-BA (**1c**) and DMSO, in the presence of nitrone **4**, was heated at 80 °C for 3 h, only [3 + 2] cycloaddition product **5a** (in quantitative NMR yield and 60% isolated yield) and anthracene (81%) were observed to form, as measured by ^1^H NMR (an indicative peak appears at 5.91 ppm) and ^11^B-NMR spectroscopy (a sharp signal at 26.3 ppm). X-ray analysis of compound **5a** confirms the formation of 1,3,4,2-dioxazaborolidines heterocycle through a formal [3 + 2] reaction pathway. To the best of our knowledge, such a borano-heterocyclic structure has not been previously reported. Interestingly, due to the steric repulsion, the aryl and *tert*-butyl planes are in *trans*-fashion to each other, with an angle of 148.68° (between the two planes). Similarly, aminoxoboranes formed from **1a** and **1c** underwent a cycloaddition reaction with phenyl-*tert*-butyl-nitrone in 61% NMR yields (Figure 4A). This reactivity can also be expanded to achieve the formal insertion of oxoborane into oxaziridines to form a similar dioxazaborolidines heterocycle (Figure 4B). In this instance, we found out that oxaziridine ring **6** rapidly opens under the reaction conditions, leading to the *in situ* formation of the corresponding nitrone,^49^ followed by its cycloaddition with oxoborane to form **6a**.

**Figure 4:**
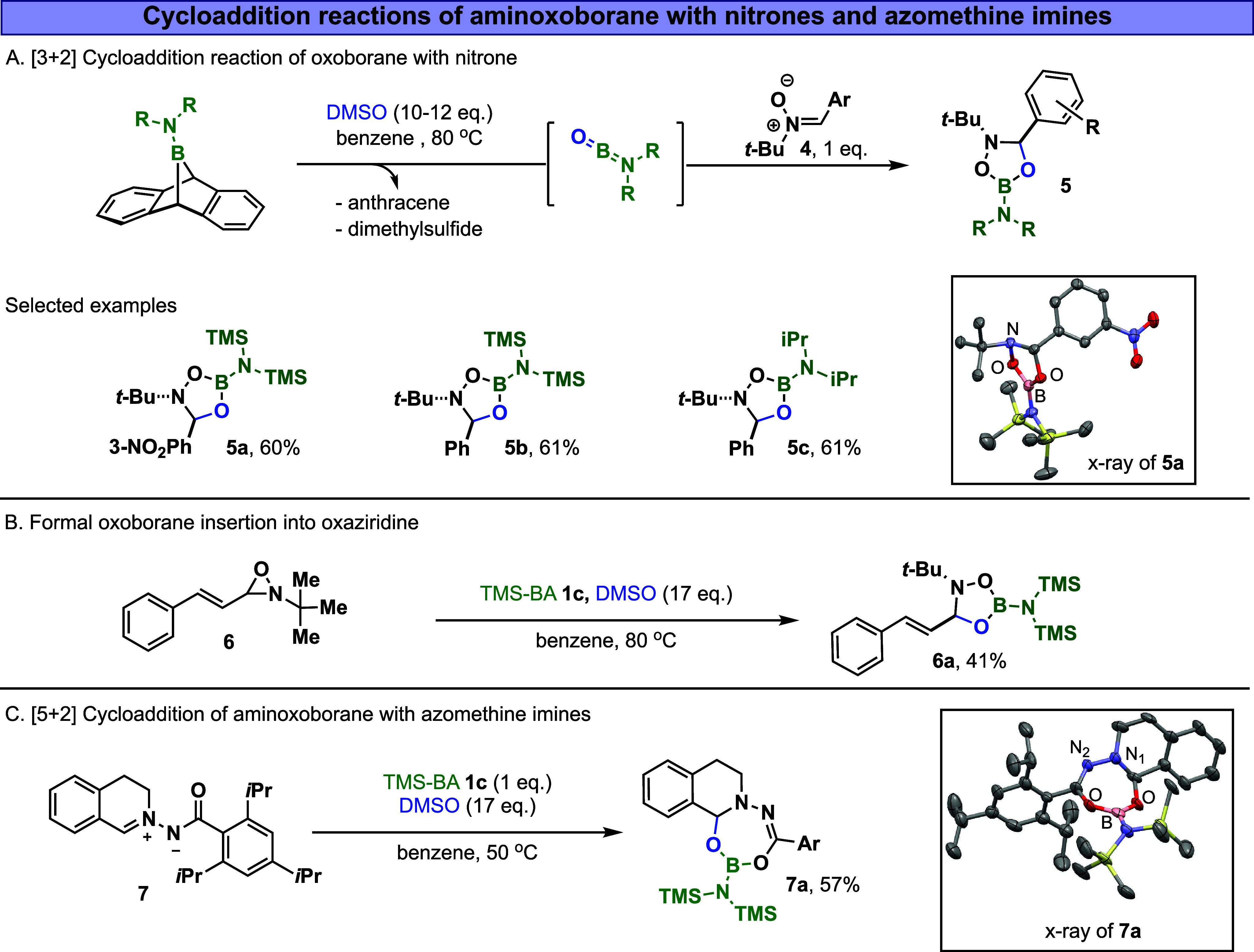
(A) [3 + 2] Cycloaddition reaction of aminoxoborane with nitrones. (B) Formal insertion of oxoborane into oxaziridine through nitrone intermediacy. (C) [5 + 2] Cycloaddition reaction of aminoxoborane with azomethine imines. Ar – triisopropylbenzene.

With these promising results for the [3 + 2] cycloaddition of aminoxoborane and nitrone, we shifted our focus to exploring other potential precursors that might undergo a similar reaction pathway - specifically, azomethine imine. To this end, we investigated the reaction of aminoxoborane, derived from **1c**, with azomethine imine **7** (1 equiv., Figure 4C). Surprisingly, we observed the formation of the [5 + 2]­product **7a** with the boracycle, 1,3,5,6,2-dioxadiazaborepino-isoquinoline, in 57% NMR yield.^50^ The product was confirmed using ^1^H NMR (an indicative peak appears at 6.05 ppm, corresponding to hemiaminal proton), ^11^B-NMR (a sharp signal at 26.3 ppm), and ^13^C NMR (an indicative peak appears at 91.7, corresponding to hemiaminal carbon). X-ray analysis confirms the formation of compound **7a**, featuring a seven-membered boracycle. Notably, the bond length between N_1_ and the adjacent hemiaminal carbon measures 1.416 Å, consistent with a typical single bond. In contrast, the bond length between N_2_ and the imidic carbon is 1.284 Å, resembling a double bond character.

**Figure 5:**
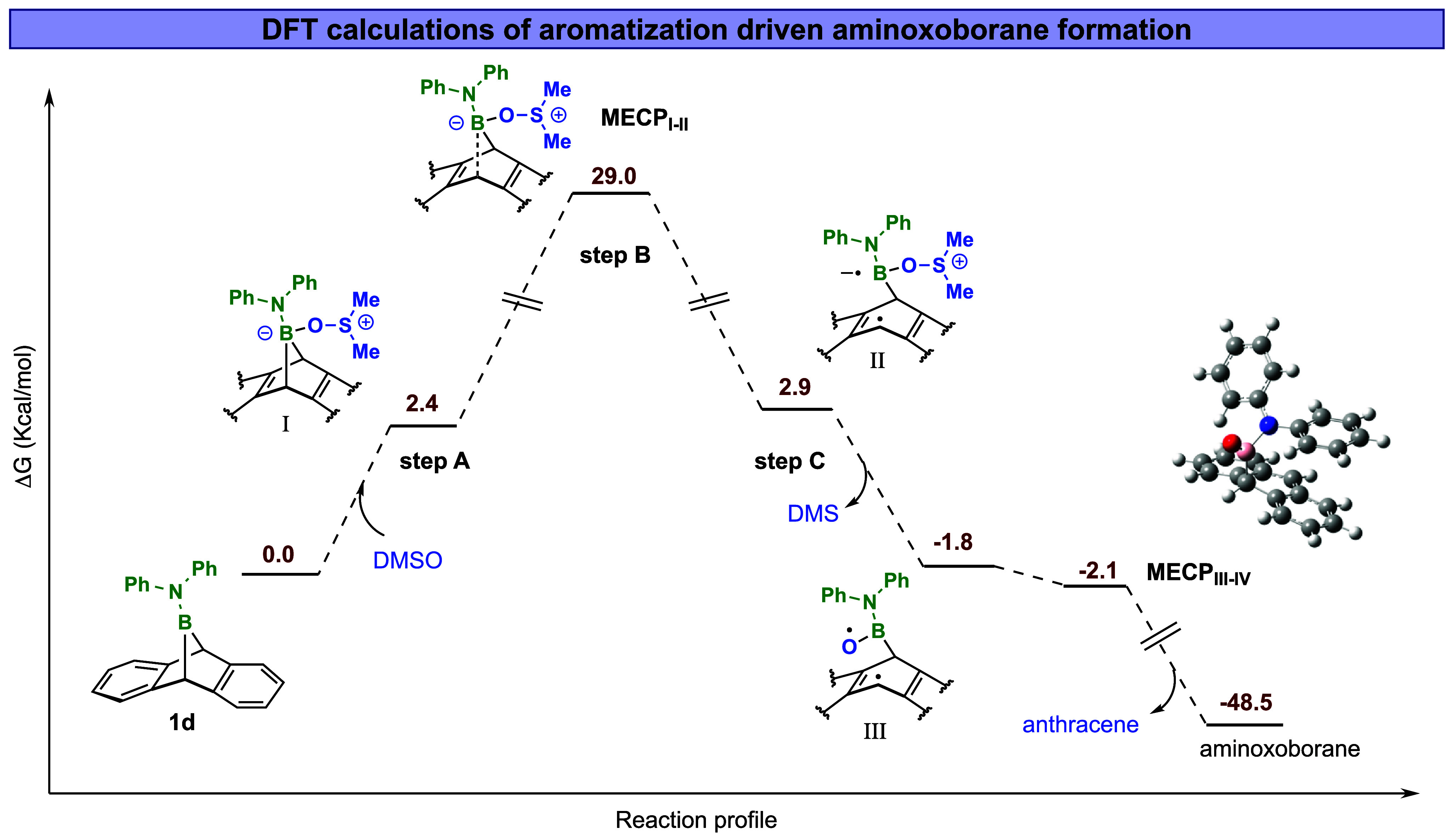
DFT computation of aromatization-driven **1d** fragmentation and aminoxoborane formation.

To gain deeper insights into oxoborane formation via the proposed aromatization pathway, we conducted a comprehensive mechanistic study by combining DFT calculations with experimental and NMR analysis. Typically, such processes can proceed through either a concerted aromatization mechanism or a stepwise route involving radical intermediates. According to our proposed mechanism, the first step is the complexation of DMSO with boranoanthracene, this adduct is supported by DFT calculations (CPCM­(C_6_H_6_)-PBE0-D3BJ/def2-TZVPPD level of theory; all free energies are corrected to match the experimentally measured concentrations in the reaction, see discussion in Computational Section) and by ^1^H- and ^11^B-NMR spectroscopy of Ph-BA (**1d**) with DMSO (Figure 6).

**Figure 6:**
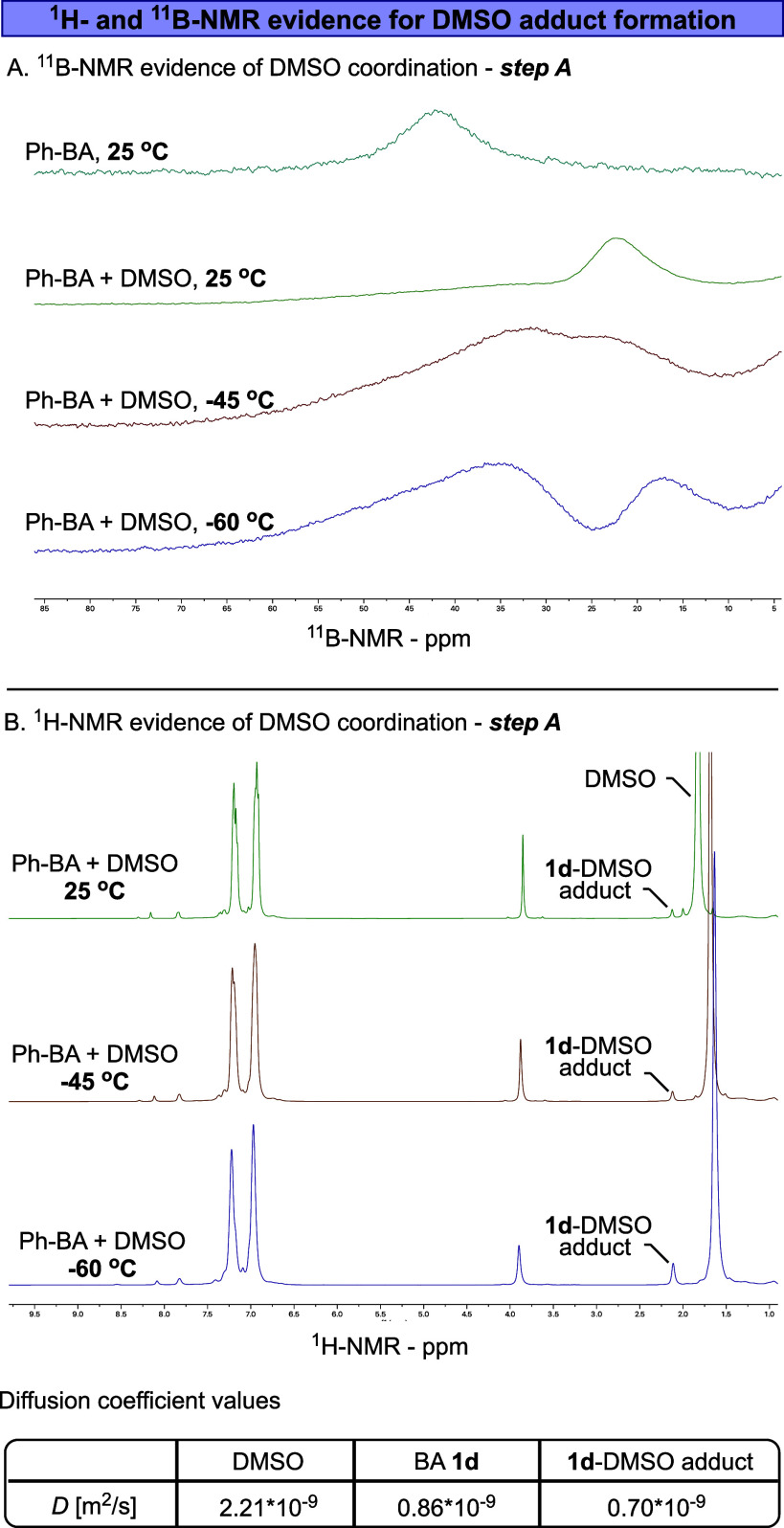
(A) ^11^B-NMR (128 MHz) of **1d** with and without DMSO (20 equiv). (B) top – ^1^H NMR (400 MHz) analysis of **1d** with DMSO (20 equiv), bottom – diffusion coefficient graphs of **1d** and its DMSO adduct, measured at room temperature. The singlet at 1.93 is a satellite of the DMSO. *D* – diffusion coefficient, m^2^/s.

The first evidence of DMSO coordination to **1d** was obtained through low-temperature ^11^B-NMR analysis. As shown in Figure 6A, **1d** has a ^11^B-NMR resonance at 41.3 ppm in toluene-*d*_*8*_ (Figure 7A-blue). However, after adding DMSO (20 equiv), this signal disappeared and was replaced by a new upfield signal at 24.3 ppm at 25 °C (Figure 6A-green).^51^ Interestingly, lowering the temperature to −45 °C resulted in a minor splitting of the boron peak (near coalescence temperature, Figure 6A-brown). However, further decreasing the temperature to −60 °C led to a complete separation of the peaks, yielding two distinct boron signals (Figure 6A-purple). The first peak, at 37.2 ppm, is attributed to the noncoordinated Ph-BA **1d**, while the second peak, at 16.9 ppm, corresponds to the DMSO-coordinated adduct. A similar behavior was observed when a titration experiment was performed by varying the amount of DMSO (5–100 equiv) added to Ph-BA **1d** (Figure S12). In this experiment, the ^11^B-NMR spectrum exhibited a significant shift, ranging from 40 to 22 ppm, depending on the concentration of DMSO in solution. This shift could be attributed to the formation of varying amounts of the DMSO-**1d** adduct in the solution.

**Figure 7:**
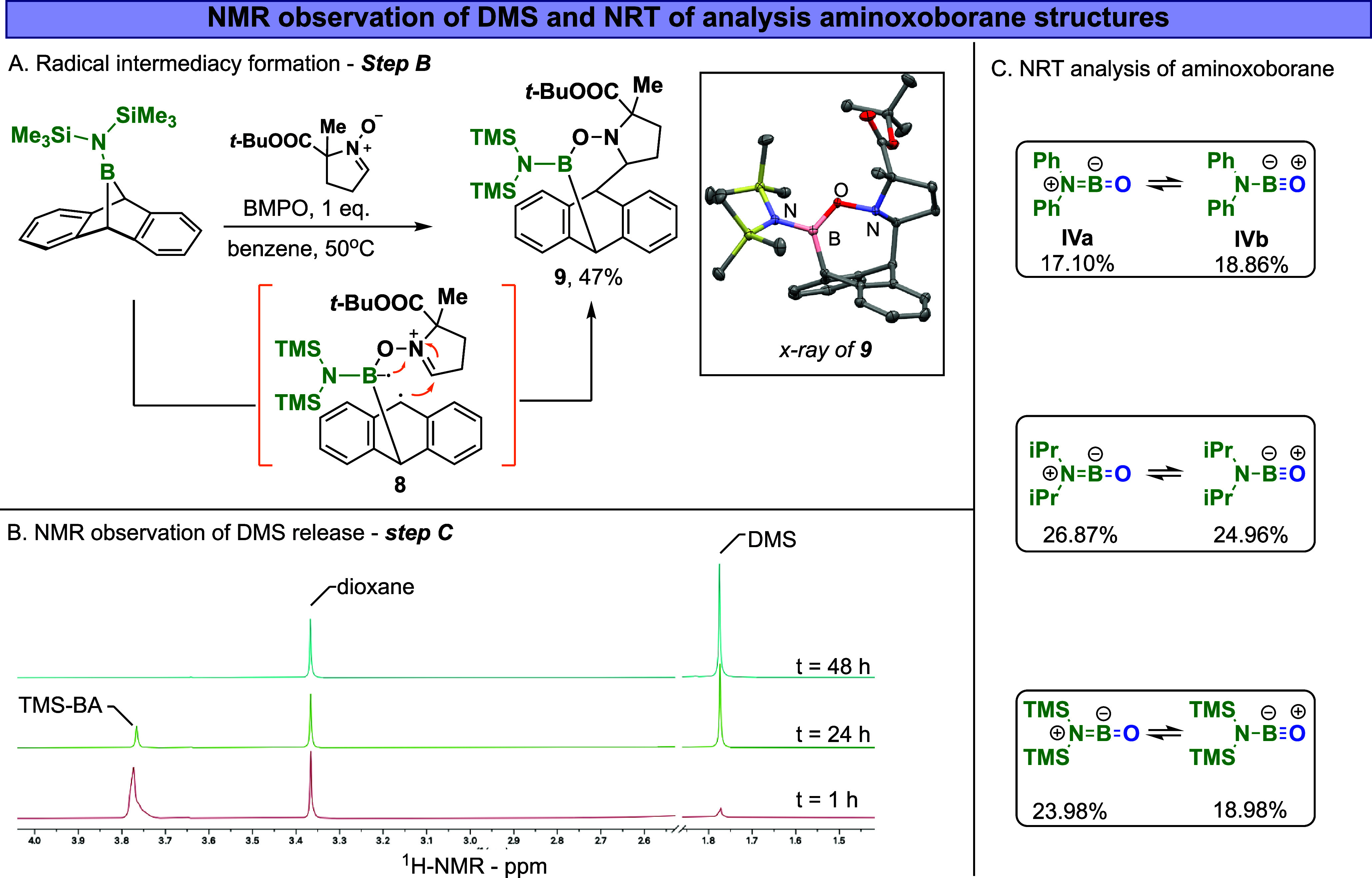
(A) Radical trapping experiment. (B) NMR observation of released dimethyl sulfide. (C) NRT analysis of aminoxoborane structures.

The second evidence for the formation of DMSO-**1d** adduct was obtained through the detection of the coordinated DMSO in the ^1^H NMR spectrum. A new signal was detected at a slightly higher chemical shift (δ 2.15 ppm) than was DMSO (δ 1.8 ppm), which likely corresponds to the methyl groups of the coordinated DMSO in adduct **I** (Figure 6B). Notably, when **1d** was consumed, the coordinated DMSO peak at 2.15 ppm also disappeared (Figure S13). To substantiate this assignment, we resorted to diffusion NMR spectroscopy (Figure 6B, bottom table). The measured diffusion coefficients of DMSO and Ph-BA (**1d**) in benzene-*d*_*6*_ are 2.21 × 10^–9^ and 0.86 × 10^–9^ m^2^/s, respectively. The peak at 2.15 ppm shows a slower diffusion coefficient of 0.70 × 10^–9^ m^2^/s, indicating the formation of a new DMSO-containing species with a higher molecular weight than that of **1d**. These findings provide additional support for the formation of adduct **I** (Figure 5). Finally, we conducted a phase-sensitive 1D-NOE experiment to further confirm that the observed new DMSO peak corresponds to a coordinated DMSO bound to the boron center in the Ph-BA (see Figure S15). Upon irradiating the 2.15 ppm peak (DMSO-**1d** adduct), we detected a strong steric interaction (positive NOE) between the bound DMSO and the aromatic protons of the boranoanthracene.

The next step in the reaction mechanism would be the formation of oxoborane. Initially, it was hypothesized that this formation would proceed through a concerted mechanism. We performed a relaxed scan of one of the B–C bond lengths in **I** to find a suitable transition state, hoping that the second B–C bond would correspondingly elongate (Figure 5). However, these scans revealed the formation of intermediate **II,** where only one of the two bonds was broken (Figure 5). This led us to consider a two-state reactivity with two minimum energy crossing points (MECP), as reviewed by Poli and Harvey.^52^ An MECP is a geometry where the energies of the two states (here, singlet and triplet) are the same, thereby easing the transition between the two states. Although the corresponding singlet intermediate of **II** is high in energy (Δ*G*_298_ = 19.6 kcal/mol, Figure S20 and Table S5), the corresponding triplet state is quite reasonable (Δ*G*_298_ = 2.9 kcal/mol). In our DFT calculations, the spin density accumulation (i.e., radical centers) in **II** (Figure S24) is localized on the sulfur and oxygen and at the anthracene C_10_ position (where B is attached to C_9_). Furthermore, the transition of **I** to **II** would require the presence of a connecting MECP (Figure 6, **MECP**_**I–II**_). However, perhaps due to the relative proximity of an MECP for the concomitant loss of DMS and the aminoxoborane (*vide infra*) to form **III**, a proper MECP could not be found. We, therefore, resorted to a quasi-MECP approach based on singlet and triplet relaxed scans (see the Computational Methods in the Supporting Information for details). Thus, a crude, upper-limit approximation of the connecting quasi-MECP was found, leading to a barrier of Δ*H*_298_^‡^= 17.5 kcal/mol, Δ*G*_298_^‡^= 29.0 kcal/mol for the Ph derivative **1d**.

The proposed stepwise radical pathway and the formation of **II** were further supported by an intramolecular radical trap experiment. More specifically, we replaced DMSO with 5-*tert*-butoxycarbonyl-5-methyl-1-pyrroline-*N*-oxide (commonly known as BMPO); we hypothesized that the zwitterionic *N*-oxide unit would function as a Lewis base, coordinating with the boron center, whereas the iminium unit could engage with any formed radicals.^53^ Indeed, when we reacted **1c** with 1 equiv of BMPO in benzene, we observed the formation of **9** in a 47% yield. One viable explanation for the formation of product **9** is through the radical addition of the benzylic radical (intermediate **8**, Figure 7A) to the iminium moiety. The structure of **9** was confirmed using X-ray analysis (Figure 7A). It is worth noting that other polar pathways could be proposed to form such a product. Moreover, when PhSiH_3_ (a radical scavenger) is added to the reaction of **1c** with DMSO, the oxoborane formation reaction is fully inhibited, likely due to a hydrogen atom transfer process with radical intermediate **II** (Figures S16–S19). Although **1c** was fully consumed, only trace amounts of anthracene and oxoborane-based products were detected by NMR analysis, along with new, unidentified boron species (Figure S16). In a blank reaction, i.e., between PhSiH_3_ and **1c** without DMSO, no reaction was observed. Overall, the synergy between the DFT calculations and the trapping experiments indicates the formation of a biradical intermediacy **II**. Conceptually similar stepwise biradical mechanisms have been reported for the aromatization formation of carbenes, ethylene dione, and silylenes.^54–57^

In the next step of the proposed mechanism (step C), the sulfur–oxygen bond (**II**) dissociates, forming DMS and intermediate **III**. The formation of DMS was observed in the reaction of **1c** by *in situ* ^1^H NMR analysis (Figure 7B). In the plot of the electronic spin density (ρ_σ_) for intermediate **III,** there is spin density accumulation on the anthracene C_10_ and the oxygen atom (Figure S25). In the final step, intermediate **III** undergoes aromatization, forming anthracene and a reactive aminoxoborane intermediate with an energy gain of 46.7 kcal/mol (Figure 5). The second **MECP**_**III–IV**_ is slightly lower in energy (by 0.3 kcal/mol) than intermediate **III**. This step is after the rate-determining step and is very exothermic; considering the approximations involved in the calculation, it is justified to state that this step – cleavage of a weak B–C bond in a reactive species – has a very low barrier that does not affect the kinetics or thermodynamics of the overall reaction. It should also be noted that the equivalent barriers for the TMS and *^i^* Pr systems are positive and very low in energy (see Table S5, Supporting Information).

Although free oxoboranes typically have a strong B–O triple bond resonance structure,^6,17,18^ natural resonance theory (NRT) analysis of aminoxoboranes revealed a significant ionic character in the B–O and B–N bonds (Figure 7C). This analysis reveals two dominant resonance structures: an allenic-like oxoborane **IVa**, which contributes 17% to the overall electronic structure, and structure **IVb**, which contributes 19%. Similar behavior is observed in other formed aminoxoboranes, as shown in Figure 7C. For comparison, we found that an NRT analysis for MeBO contributes 87% from a Me-B≡O resonance structure (see Figure S29, Supporting Information).^5a^

The aromatization-driven formation of the reactive main group species generally proceeds through a redox-neutral process, where fragmentation results in the formal oxidation of the hydrocarbon framework and the formation of an aromatic ring alongside the reduced reactive species.^29,33,40^ In our proposed mechanism for oxoborane formation, however, the involvement of a noninnocent Lewis base facilitates a formal oxidative aromatization that drives the generation of the reactive species. To the best of our knowledge, this represents the first example of such a process in this field.

## Conclusions

To summarize, we introduced a new class of boranoboradiene derivatives, which we termed boranoanthracenes. Using these versatile precursors, we proposed a novel mechanism for generating free oxoborane species. This mechanism is initiated by the coordination of an oxygen-Lewis base to the boron center, triggering a fragmentation pathway driven by oxidative aromatization. This pathway enables the formation of aminoxoborane species, which are rarely reported in the literature. We discovered three distinct reactivities of these species: first, the insertion of oxoborane species into B–C bonds, as observed with oxoboranes derived from TMS-BA **1c** and Ph-BA **1d**, representing, to the best of our knowledge, the first example of this reactivity. Second, the [3 + 2] cycloaddition reaction with nitrones yields new 5-membered boranoheterocycles from oxoboranes formed *via i*Pr-BA **1a** and TMS-BA **1c**. Finally, we demonstrated the first example of a [5 + 2] cycloaddition of oxoboranes with azomethine imine to form a 7-membered boracycle. The varied reactivities of the aminoxoboranes in this context reveal the untapped potential of these species in advancing organic chemistry and synthetic methodologies.

## Supplementary Material





## Notes

Full details of the experimental procedures for the synthesis of all new substances together with characterization data, including details of X-ray diffraction studies and Cartesian coordinates for structures optimized by computational methods (PDF)
Cartesian coordinates for structures (TXT)
