# Correction to “Bridged Boranoanthracenes: Precursors for Free Oxoboranes through Aromatization-Driven Oxidative Extrusion”

**DOI:** 10.1021/jacs.6c09597

**Published:** 2026-08-31

**Authors:** Stav Deri, Moran Feller, Shibaram Panda, Batya Blank, Mark A. Iron, Yael Diskin-Posner, Liat Avram, Linda J. W. Shimon, Rakesh Mondal, Samer Gnaim

A citation is being added in the first two paragraphs of the introduction section (page 19520) to cite Sukhram’s thesis properly.^1^ These two paragraphs are similar in structure, phrasing, and content to pages 33–34 of the undergraduate thesis, therefore requiring citation. Additionally, the authors disclose the use of AI tools for text generation. Specifically, for the summary of oxoborane chemistry; part of this text was used in the first two paragraphs of the Introduction.

The authors note that this addition does not affect any data, results, conclusions, or interpretations reported in our Article.
