# Supplementary material for: Bridged Boranoanthracenes: Precursors for Free Oxoboranes through Aromatization-Driven Oxidative Extrusion
Source: J Am Chem Soc. 2025 May 12;147(23):19520–9. doi: 10.1021/jacs.4c15496 (PMC12164262; doi:10.1021/jacs.4c15496)
Supplement: Supplementary file 1 [file ja4c15496_si_001.pdf]

Bridged Boranoanthracenes: Precursors for Free Oxoboranes through Aromatization-Driven Oxidative Extrusion

Stav Deri[#a], Moran Feller[#a], Shibaram Panda[#a], Batya Blank[a], Mark A. Iron[b], Yael Diskin-Posner[b], Liat Avram[b], Linda J. W. Shimon[b], Rakesh Mondal[a], and Samer Gnaim[*a]

[a] Department of Molecular Chemistry and Materials Science, Weizmann Institute of Science, Rehovot 7610001, Israel.

[b] Department of Chemical Research Support, Weizmann Institute of Science, Rehovot 7610001, Israel.

[#] S.D., M.F. and S.P. contributed equally to this paper.

Table of Contents

**1.**	**Experimental details**			
	**1.1**	**Methods and materials**		3
	**1.2**	**Source of reagents**		4
	**1.3**	**Synthetic procedures, NMR and X-ray crystallographic data**		5
		1.3.1	Synthesis of borano-dihalides **2a-2d**	5
		1.3.2	Synthesis of Mg-anthracene and nitrones	6
		1.3.3	Synthesis of boranoanthracenes **1a-1d**	10
		1.3.4	Synthesis of insertion products **3c** and **3d**	18
		1.3.5	Synthesis of trapping products **5a-5c**	21
		1.3.6	Synthesis of product **6a**	26
		1.3.7	Synthesis of product **7a**	27
		1.3.8	Synthesis of radical trap product **9**	29
**2.**	**X-ray tables**			31
**3.**	**Additional data: NMR kinetics and control experiments**			34
**4.**	**Computational details**			51
**5.**	**^{1}H-, ^{11}B-, ^{13}C-NMR spectra**			79
**6.**	**References**			129

1. Experimental details

1.1. Methods and materials

All air- and moisture-sensitive reactions were carried out under nitrogen using standard Schlenk techniques or a glovebox. All glassware used for reactions were oven-dried overnight at 140 °C. Reagents were purchased at the highest commercial quality and used without further purification unless otherwise stated. Isolated yields refer to chromatographically and spectroscopically (^{1}H-NMR) homogeneous material unless otherwise stated. Chloroform ($CHCl_3$) dichloromethane (DCM), *n*-pentane, *n*-hexane, 1,2-dimethoxyethane (DME), benzene, toluene, dimethylsulfoxide (DMSO), diethylether, and tetrahydrofuran (THF) were obtained by passing the previously degassed solvents through an activated alumina column. For the determination of ^{1}H-NMR yields, dioxane was used as an internal standard (automatic baseline correction was applied). Reactions were monitored by thin layer chromatography (TLC) carried out on 0.25 mm E. Merck silica plates (60 F_{254}), using short-wave UV light (254 nm) for visualization, and *p*-anisaldehyde or potassium permanganate as developing agents. Flash column chromatography was performed using E. Merck silica gel (60, particle size 0.043–0.063 mm) or basic Al_2O_3. NMR spectra were recorded on Bruker 7T (300MHz) AVANCE NEO, 9.4T (400MHz) AVANCE NEO and 11.7T (500MHz) AVANCE IIIHD instruments, and chemical shifts for ^{1}H- and ^{13}C-NMR are reported relative to the solvent peaks (7.26 ppm for ^{1}H-NMR in $CDCl_3$, 77.16 ppm for ^{13}C-NMR in $CDCl_3$) and (7.16 ppm for ^{1}H-NMR in C_6D_6, 128.39 ppm for ^{13}C-NMR in C_6D_6). The following abbreviations were used to explain NMR peak multiplicities: s = singlet, d = doublet, t = triplet, q = quartet, p = pentet, m = multiplet, br = broad.

The mass spectroscopy analysis was conducted on a Waters Xevo G2-XS QTof mass spectrometer (Manchester, UK) with an electrospray ionization (ESI) source operating in negative mode. The solutions were directly infused at a flow rate of 10 µL/min. All spectra were acquired in the mass range of 50–2000 m/z. The mass errors of the analyzed spectra are no more than 5.0 ppm. Analyses were performed using a capillary voltage of 2.20 kV, cone gas flow of 50 L/h, source temperature was set at 120 °C and cone voltage of 40V.

The desolvation temperature was set at 250 °C, and the desolvation gas (N_2) flow rate was set at 800 L/h. All measurements were done using Leucine-Enkephalin (200 µg/uL, acetonitrile:H_2O containing 0.1% formic acid (1:1, v/v)) as a lockspray reference at a flow rate of 10 uL/min to ensure mass accuracy and follow resolution mode. Data acquisition and recording were done by Waters MassLynx v4.2 software.

GC-MS (EI) was recorded on Agilent 7820A GC systems and 5975 Series MSD using *n*-decane as an internal standard.

Single crystals of complexes CCDC 2389617 - 2389625 were obtained by cooling at -35 ºC or slow evaporation. All crystals were coated in oil and flash-frozen in a liquid nitrogen stream. Data were collected at 100 K. Data for structure CCDC 238622 and 2389625 were collected on a Rigaku Synergy-R diffractometer equipped with a HyPix ARC 150º detector and CuKa (l=1.54184Å). Data for structures CCDC 2389617, 2389618, 2389621, 2389623 and 2389624 were collected on a Rigaku Synergy-R diffractometer with HyPix ARC 100º detector, MoKa (l=0.71073Å). CCDC 238619 and CCDC 2389620 were collected on a Rigaku Synergy-S diffractometer dual source equipped with Dectris Pilatus3 R CdTe 300K detector, MoKa (l=0.71073Å). All datasets were processed with CrysAlisPRO, and structures were solved with SHELXT1. All non-hydrogen atoms were further refined by SHELXL2 with anisotropic displacement coefficients. Hydrogens were mostly placed in calculated positions and refined in a riding mode. A few hydrogens were located in the electron density map, incorporated and refined. Refinement was carried out with the OLEX-23 GUI.[1-3]

1.2. Source of reagents

BCl_3, BBr_3, *n*-butyl lithium, anthracene, lithium di-*iso*-propylamide, lithium hexhamethyldisilazane, diphenylamine, and *N*-*tert*-butyl-α-phenylnitrone (PBN) were obtained from Merck and Sigma-Aldrich.

1.3. Synthetic procedures, NMR and X-ray crystallographic data

<u>Starting material synthesis</u>

Compound 2a

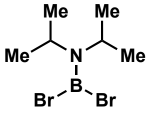

The preparation of the following starting material was carried out according to the literature procedures.[4]

The ^{1}H- and ^{11}B-NMR analysis matches the reported data.

^{1}H-NMR (300 MHz, benzene-d_6) δ (ppm): 4.12 (br, 2H), 1.33 (d, J = 5.9 Hz, 12H).

^{11}B-NMR (96 MHz, benzene-d_6) δ (ppm): 25.01 (s).

Compound 2c

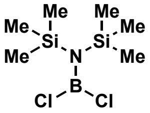

The preparation of the following starting material was carried out according to the literature procedures.[5]

The ^{1}H- and ^{11}B-NMR analysis matches the reported data.

^{1}H-NMR (300 MHz, benzene-d_6) δ (ppm): 0.36 (s, 18H).

^{11}B-NMR (96 MHz, benzene-d_6) δ (ppm): 37.64 (s).

Compound 2d

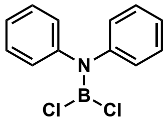

The preparation of the following starting material was carried out according to the literature procedures.[6]

The ^{1}H- and ^{11}B-NMR analysis matches the reported data.

^{1}H-NMR (300 MHz, $CDCl_3$) δ (ppm): 7.44 – 7.34 (m, 4H), 7.32 – 7.19 (m, 6H).

^{11}B-NMR (96 MHz, benzene-*d*$_6$) δ (ppm): 33.52 (s).

Compound 2b

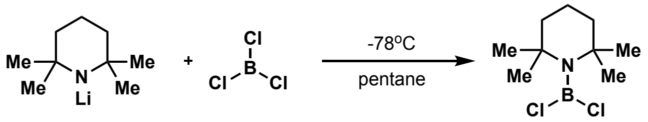

Inside a glove box, 499 mg of lithium 2,2,6,6-tetramethylpiperidide (1.0 eq, 3.3 mmol) was added to a round-bottom flask containing 60 mL of dry *n*-pentane. The flask was taken out of the glove box and was adjusted to a Schlenk line, and cooled to -78 °C using acetone and a dry ice bath under a nitrogen atmosphere. Next, 3.7 mL of 1M BCl_3 (1.1 eq, 3.7 mmol) was added dropwise. The reaction mixture was allowed to warm to room temperature and left to stir overnight under nitrogen. A clear solution with a white precipitate was formed. The flask was introduced to the glove box, and the mixture was filtered using vacuum filtration. The filtrate was then evaporated under reduced pressure. A yellow oil was obtained after evaporation and stored in the freezer, yielding a 79% product **2b**.

^{1}H-NMR (300 MHz, benzene-*d*$_6$) δ (ppm): 1.36 (s, 12H), 1.33 (m, 6H).

^{13}C{^{1}H}-NMR (126 MHz, benzene-*d*$_6$) δ (ppm): 56.1, 34.7, 31.0, 14.0.

^{11}B-NMR (96 MHz, benzene-*d*$_6$) δ (ppm): 34.42 (s).

Compound S5

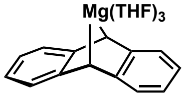

The preparation of the following starting material was carried out according to the literature procedures.[7]

Compound 4

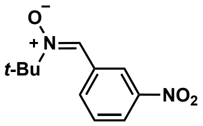

The preparation of the following starting material was carried out according to the literature procedures.[8]

The ^{1}H-NMR analysis matches the reported data.

^{1}H-NMR (300 MHz, $CDCl_3$) δ (ppm): 9.12 (t, *J* = 2.0 Hz, 1H), 8.67 (d, *J* = 7.8 Hz, 1H), 8.22 (ddd, *J* = 8.3, 2.4, 1.1 Hz, 1H), 7.69 (s, 1H), 7.59 (t, *J* = 8.1 Hz, 1H), 1.63 (s, 9H).

Compound S1

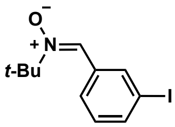

In a round-bottom flask, a solution of an equimolar amount of the suitable N-hydroxyl ammonium-acetate (0.64 gr, 4.3 mmol, 1.0 eq) and 3-iodo-benzaldehyde (1 gr, 4.3 mmol, 1.0 eq.) in DCM (13 mL), and pyrrolidine (0.4 mL, 4.8 mmol, 1.1 eq) was added. The mixture was stirred at room temperature, and the reaction was monitored by TLC until the consumption of aldehyde was almost complete. Then, the reaction crude was filtered through a short silica pad and washed with EtOAc to obtain a crude mixture. Silica gel chromatography, 15-50% EtOAc/Hexane, was performed to furnish the desired **S1** in 83% (1.08 gr, 3.6 mmol).

^{1}H-NMR (400 MHz, $CDCl_3$) δ (ppm): 8.84 (t, *J* = 1.6 Hz, 1H), 8.12 (d, *J* = 8.0 Hz, 1H), 7.74 – 7.70 (m, 1H), 7.49 (s, 1H), 7.14 (t, *J* = 7.9 Hz, 1H), 1.61 (s, 9H).

^{13}C{^{1}H}-NMR (101 MHz, $CDCl_3$) δ (ppm): 138.8, 137.0, 132.9, 130.0, 128.4, 127.8, 94.3, 71.3, 28.3.

TLC: R_f = 0.4 (8.5:1.5 Hexane: EtOAc).

MS (TOF) m/z calcd $C_{11}H_{14}INONa$: 326.0120, found: 326.0024.

Compound 7

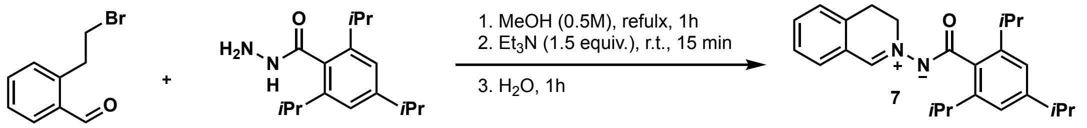

Procedure for the Synthesis of Iminium ylide: 2,4,6-triisopropylbenzohydrazide (2 mmol, 1 equiv, 525 mg) was added to a methanolic solution (0.5 M) of 2-(2-bromoethyl)benzaldehyde (2.1 mmol, 1.05 equiv, 448 mg) and the reaction mixture was refluxed for 1 h. After cooling to room temperature, 0.42 mL of Et_3N (3 mmol, 1.5 equiv) was added to the reaction mixture and stirred for 15 min. To this mixture, 10 mL of H_2O was added and stirred for 30 min. The organic phase was extracted with CH_2Cl_2 (30 mL, 3 times), dried over Na_2SO_4 and concentrated under reduced pressure. The crude reaction mixture was purified by silica-gel column chromatography with EtOAc/Hexane (30:70) as an eluent to get the pure product as a yellow solid with a 93 % yield (700 mg).

^{1}H-NMR (300 MHz, $CDCl_3$) δ (ppm): 9.41 (s, 1H), 7.47 (t, J = 6.5 Hz, 2H), 7.41 – 7.32 (m, 1H), 7.28 (d, J = 7.3 Hz, 1H), 7.01 (s, 2H), 4.18 (t, J = 7.3 Hz, 2H), 3.19 (dt, J = 20.0, 7.0 Hz, 4H), 2.89 (dt, J = 13.7, 6.8 Hz, 1H), 1.27 (dd, J = 11.6, 6.9 Hz, 18H).
^{13}C{^{1}H}-NMR (75 MHz, $CDCl_3$) δ (ppm): 172.8, 148.6, 145.0, 133.9, 132.8, 129.7, 127.9, 127.8, 127.4, 120.6, 53.6, 34.5, 31.0, 26.5, 24.5, 24.1.
TLC: R_f = 0.25 (70:30 Hexane: EtOAc).
MS (TOF): m/z calcd $C_{25}H_{33}N_2O$: 377.2515, found: 377.2592.

Crystallographic data for **7**

Crystal data: $C_{25}H_{32}N_2O$, light yellow chunk, 0.491 x 0.468 x 0.416 mm^3, Monoclinic *$P2_1/n$*, a=10.6136(2)Å, b=13.4079(2)Å, c=15.3481(3)Å, α=90°, β=99.604(2)°, γ=90°, from 41969 reflections, 2Θ range for data collection 5.304° to 61.794°, T=100(2)K, V=2153.52(8)$Å^3$, Z=4, Fw=376.52, Dc=1.161 $Mg{\cdot}m^{-3}$, μ=0.070 mm^{-1}.

Data collection and processing: Rigaku Synergy R diffractometer equipped with Hypix-Arc 100 detector, MoKα (λ=0.71073Å), -13≤h≤14, -19≤k≤17, -21≤l≤21, frame scan width = 0.25°, scan speed 1.0° per 0.48 sec, 41969 reflections collected, 6011 independent reflections (R-int =0.0380). The data were processed with CrysAlis^{PRO}.

Solution and refinement: Structure solved with SHELXT program. Full matrix least-squares refinement based on F^2 with SHELXL on 259 parameters with no restraints gave final R_1= 0.0496 (based on F^2) for data with I>2σ(I), wR_2= 0.1349 on 6011 reflections, and final R_1= 0.0598 (based on F^2), wR_2= 0.1427 for all data, goodness-of-fit on F^2 = 1.034 largest electron density peak 0.53 $e{\cdot}Å^{-3}$. Largest hole -0.21 $e{\cdot}Å^{-3}$.

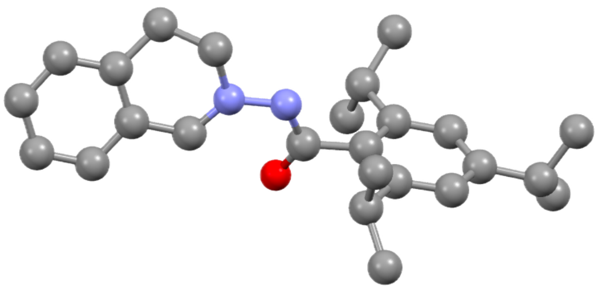

Bridged boranoanthracenes synthesis

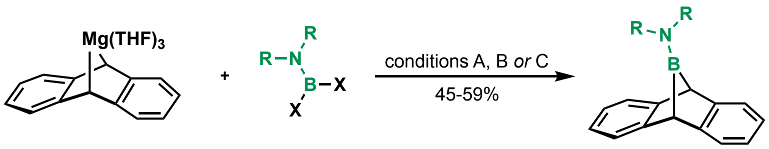

Synthesis of ^{i}Pr-BA (**1a**)

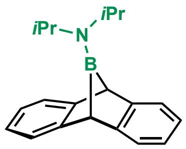

A solution of $^iPr_2NBBr_2$ (0.96 g, 3.54 mmol) in 15 mL of DME was slowly added to an oven-dried 100 mL round-bottom flask containing Mg-anthracene·3THF (1.49 g, 3.54 mmol) in 15 mL of DME. The mixture was stirred at room temperature for 2 h. The crude mixture was filtered and washed thrice with 5 mL of pentane. Volatile materials were removed under reduced pressure. The residue was dissolved in 20 mL of *n*-pentane and filtered, and the volatile components were slowly removed under reduced pressure to obtain the solid crude mixture. The clean solid product was obtained by washing the solid with cold pentane, 0.58 g (2.00 mmol, 56%).

^{1}H-NMR (300 MHz, benzene-*d*$_6$) δ (ppm): 7.34 (dd, *J* = 5.3, 3.2 Hz, 4H), 7.03 (dd, *J* = 5.3, 3.2 Hz, 4H), 3.79 (s, 2H), 3.18 – 3.02 (m, 2H), 0.80 (d, *J* = 6.8 Hz, 12H).
^{13}C{^{1}H}-NMR (126 MHz, benzene-*d*$_6$) δ (ppm): 146.7, 125.4, 122.1, 47.4, 45.4, 23.1.
^{11}B-NMR (96 MHz, benzene-*d*$_6$) δ (ppm): 41.51 (s).
MS (TOF) m/z calcd for $C_{20}H_{25}BN$: 290.2002, found: 290.2051.

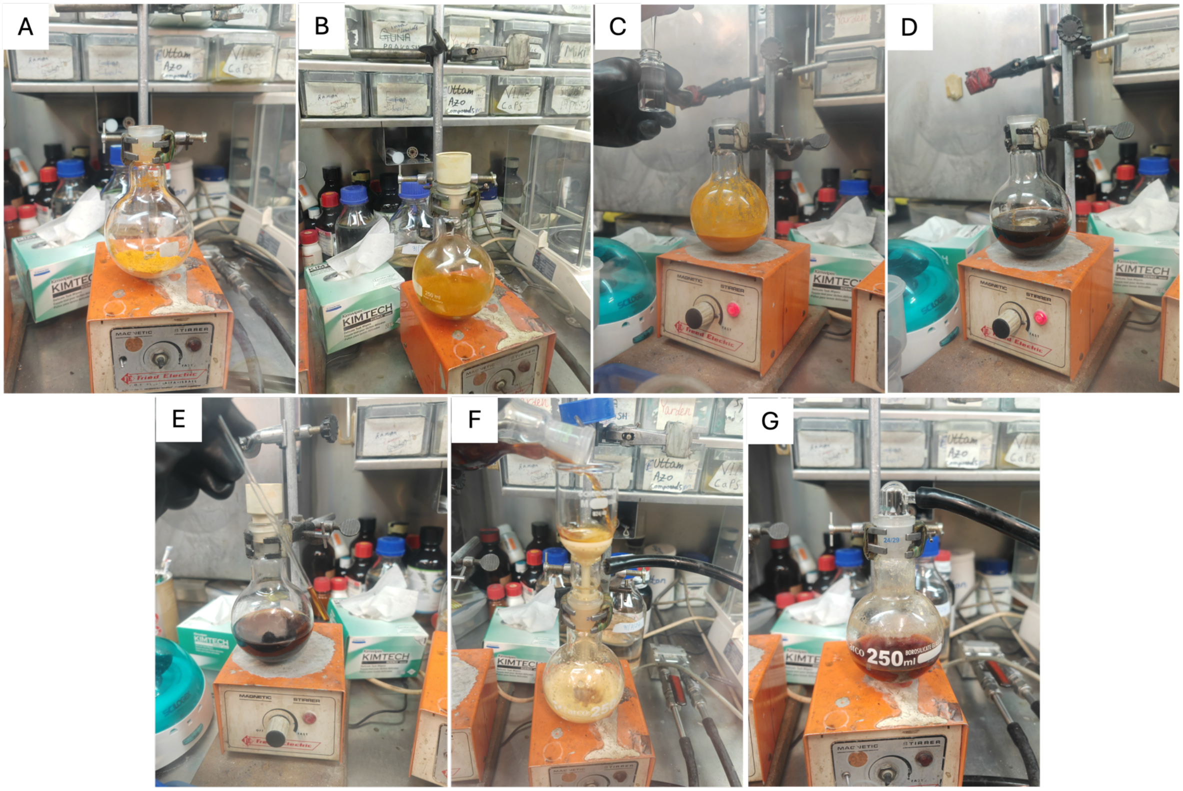

Scheme S1: General scheme of reaction set-up. A) Magnesium anthracene solid in a 100-250 mL flask. B) Dissolved in DME/THF or benzene – low solubility. C) Slow addition of a solution of borane dihalide to the reaction mixture. D) Stir the reaction at room temperature for 2-3 hours. E) Crude NMR analysis of the reaction mixture. F) Filter the residues of magnesium anthracene and other salts. G) Evaporation of solvent to obtain the crude mixture.

Crystallographic data for **1a**

Crystal data: $C_{20}H_{24}BN$, colorless plate, 0.25 x 0.2 x 0.09 mm^3, Monoclinic *P2₁/n*, a=9.4832(4)Å, b=12.5551(5)Å, c=14.4109(5)Å, α=90°, β=92.244(4)°, γ=90°, from 46818 reflections, T=100(2)K, V=1714.48(12)$Å^3$, Z=4, Fw=289.21, Dc=1.120 $Mg{\cdot}m^{-3}$, μ=0.063 mm^{-1}.

Data collection and processing: Rigaku Synergy S dual source diffractometer equipped with Dectris Pilatus3R CdTe 300K detector, MoKα (λ=0.71073Å), -12≤h≤13, -17≤k≤17, -18≤l≤20, frame scan width = 0.25°, scan speed 1.0° per 28 sec, 46818 reflections collected, 5135 independent reflections (R-int =0.0514). The data were processed with $CrysAlis^{PRO}$.

Solution and refinement: Structure solved with the SHELXT program. Full matrix least-squares refinement based on F^2 with SHELXL on 203 parameters with no restraints gave final R_1= 0.0501 (based on F^2) for data with I>2σ(I), wR_2= 0.1131 on 5135 reflections, and final R_1= 0.0754 (based on F^2), wR_2= 0.1221 for all data, goodness-of-fit on F^2 = 1.026 largest electron density peak 0.316 $e{\cdot}Å^{-3}$. Largest hole -0.229 $e{\cdot}Å^{-3}$.

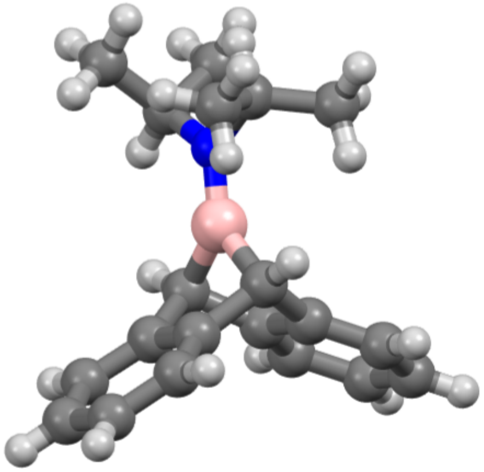

Synthesis of TMP-BA (**1b**)

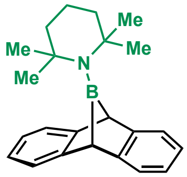

In a nitrogen-filled glovebox, 0.171 g (0.4 mmol, 1 eq) of MgA·3THF was placed into a vial. To this, 2 mL of a DME was added, followed by the addition of 0.1 g (0.45 mmol, 1.1 eq) of TMP-BCl_2. An additional 3 mL of DME was added, causing the reaction mixture to turn green. The reaction was allowed to proceed at room temperature in the glove box. After 2 h, a dark blue liquid with a blue precipitate formed. The DME was evaporated under reduced pressure. The product was then washed with *n*-pentane and filtered through a syringe filter. The final liquid was evaporated under reduced pressure. The remaining solid was washed with diethylether, filtered through a syringe filter and evaporated under reduced pressure. Both fractions gave 0.1711 g of product (50% yield). The *n*-pentane fraction was dissolved in DCM, transferred into a vial with a slightly open cap, and evaporated slowly in the glove box to obtain crystals for X-ray analysis.

^{1}H-NMR (300 MHz, benzene-d_6) δ (ppm): 7.34 (dd, J = 5.3, 3.2 Hz, 4H), 7.06 (dd, J = 5.3, 3.1 Hz, 4H), 3.94 (s, 2H), 1.18 – 1.16 (m, 6H), 1.15 (s, 12H).
^{13}C{^{1}H}-NMR (126 MHz, benzene-d_6) δ (ppm): 145.8, 125.1, 121.7, 53.9, 49.7, 38.2, 32.5, 15.1.
^{11}B-NMR (96 MHz, benzene-d_6) δ (ppm): 43.48 (s).
MS (TOF) m/z calcd for $C_{23}H_{28}BN$: 329.2302, found: 329.2312.

Crystallographic data for **1b**

Crystal data: $C_{23}H_{28}BN$, colorless needle, 0.068 x 0.050 x 0.037 mm^3, Orthorhombic $P2_12_12_1$, a=8.731(1)Å, b=14.239(2)Å, c=14.994(2)Å, α=β=γ=90°, from 6892 reflections, T=100(2)K, V=1864.1(4)$Å^3$, Z=4, Fw=329.27, Dc=1.173 $Mg{\cdot}m^{-3}$, μ=0.494 mm^{-1}

Data collection and processing: Rigaku Synergy R dual source diffractometer equipped with Hypix-Arc 150 detector, CuKα (λ=1.54184Å), -8≤h≤7, -13≤k≤12, -14≤l≤14, frame scan width = 0.5°, scan speed 1.0° per 60 sec, 6892 reflections collected, 1693 independent reflections (R-int =0.1186). The data were processed with $CrysAlis^{PRO}$.

Solution and refinement: Structure solved with the SHELXT program. Full matrix least-squares refinement based on F^2 with SHELXL on 230 parameters with no restraints gave final R_1= 0.0770 (based on F^2) for data with I>2σ(I), wR_2= 0.1753 on 1693 reflections, and final R_1= 0.1170 (based on F^2), wR_2= 0.1991 for all data, goodness-of-fit on F^2 = 0.989 largest electron density peak 0.252 $e{\cdot}Å^{-3}$. Largest hole -0.196 $e{\cdot}Å^{-3}$.

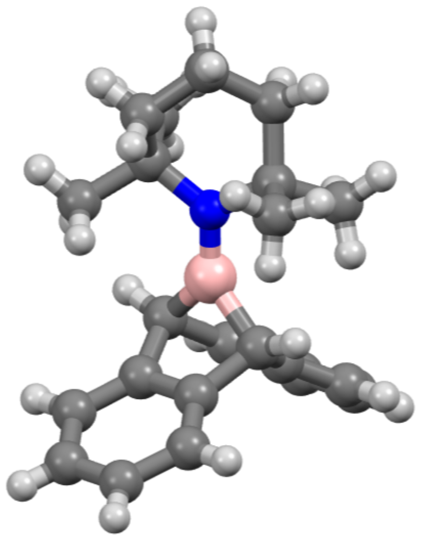

Synthesis of TMS-BA (**1*c***)

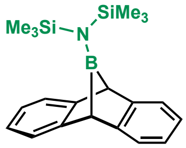

In a nitrogen-filled glovebox, a solution of TMS_2NBCl_2 (1.00 g, 4.1 mmol, 1.0 eq) in 40 mL of dry THF was added dropwise to an oven-dried 100 mL round-bottom flask containing MgA·3THF (1.9 g, 4.5 mmol, 1.1 eq) dissolved in 15 mL of dry THF. The reaction mixture was stirred at room temperature for 3 hours. After completion, the reaction mixture was filtered, and the solid residue was washed with 10 mL of dry *n*-pentane. The filtrate was concentrated under reduced pressure, yielding a dark brown solid, which was subsequently washed with three portions of dry *n*-pentane (3 × 20 mL). The resulting slurry was filtered, affording a yellow liquid, which was further evaporated to obtain the pure product **1c** as a yellow solid (1.06 g, 3.05 mmol, 75% yield).

^{1}H-NMR (300 MHz, benzene-d_6) δ (ppm): 0.08 (s, 16H), 3.89 (s, 2H), 7.04 (dd, J = 5.4, 3.2 Hz, 4H), 7.32 (dd, J = 5.3, 3.2 Hz, 4H).
^{13}C{^{1}H}-NMR (126 MHz, $CDCl_3$) δ (ppm): 144.8, 125.3, 122.2, 47.7, 3.8.
^{11}B-NMR (96 MHz, benzene-d_6) δ (ppm): 45.74 (s).
MS (TOF) m/z calcd for $C_{20}H_{29}BNSi_2$: 350.1853, found: 350.1867.

Crystallographic data for **1c**

Crystal data: $C_{20}H_{28}BNSi_2$, colorless prism, 0.259 x 0.183 x 0.101 mm^3, Orthorhombic $P2_12_12_1$, a=9.25248(18)Å, b=14.5350(3)Å, c=15.1098(3)Å, α=90°, β=90°, γ=90°, from 24960 reflections, T=100(2)K, V=2032.03(7)Å^3, Z=4, Fw=349.42, Dc=1.142 Mg·m^{-3}, μ=0.176 mm^{-1}.

Data collection and processing: Rigaku Synergy R diffractometer equipped with Hypix-Arc 100° detector, MoKα (λ=0.71073Å), -13≤h≤13, -20≤k≤18, -20≤l≤21, frame scan width = 0.5°, scan speed 1.0° per 1.16 sec, 24960 reflections collected, 6208 independent reflections (R-int =0.0352). The data were processed with CrysAlis^{PRO}.

Solution and refinement: Structure solved with the SHELXT program. Full matrix least-squares refinement based on F^2 with SHELXL on 223 parameters with no restraints gave final R_1= 0.0333 (based on F^2) for data with I>2σ(I), wR_2= 0.0831 on 7583 reflections, and final R_1= 0.0388 (based on F^2), wR_2= 0.0858 for all data, goodness-of-fit on F^2 = 1.056 largest electron density peak 0.262 e·Å^{-3}. Largest hole -0.227 e·Å^{-3}.

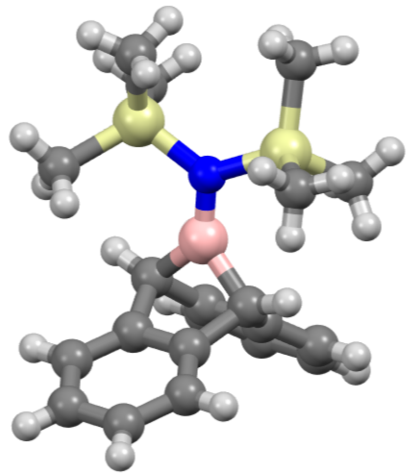

Synthesis of Ph-BA (**1d**)

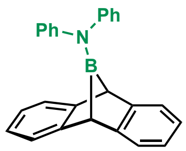

In a nitrogen-filled glovebox, a solution of Ph_2NBCl_2 (0.83 g, 3.3 mmol) in 20 mL of benzene was added dropwise to a suspension of MgA·3THF (1.4 g, 3.3 mmol) in 60 mL of benzene over 10 minutes. The mixture was stirred at room temperature for 2 h, during which the solution turned colorless, and a brown precipitate formed. The solvent was then removed under vacuum, and the remaining solid was extracted three times with 30 mL of *n*-pentane each. The combined *n*-pentane extracts were concentrated under reduced pressure, yielding Ph_2N-BA as a white solid with a 59% yield (0.70 g, 1.96 mmol) and containing 3% anthracene as an impurity.

^{1}H-NMR (300 MHz, benzene-d_6) δ (ppm): 3.83 (s, 2H), 6.83 – 6.77 (m, 4H), 6.91 – 6.8 (m, 2H), 6.98 – 6.93 (m, 4H), 7.00 (dd, 4H, 5.3, 5.2 Hz), 7.26 (dd, 4H, 5.3, 5.2 Hz).
^{13}C{^{1}H}-NMR (126 MHz, benzene-d_6) δ (ppm): 146.6, 146.1, 129.2, 126.0, 126.0, 125.6, 122.8, 45.1 (br).
^{11}B-NMR (96 MHz, benzene-d_6) δ (ppm): 41.3 ppm (br s).
MS analysis could not be obtained due to low stability of the compound.

Insertion of oxoborane into **1c** (product **3c**)

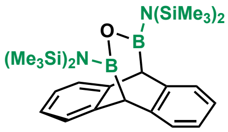

In a nitrogen-filled glovebox, a solution of $(TMS)_2N$-BA (**3*c***) (50 mg, 0.14 mmol) in 0.4 mL of benzene-d_6 and DMSO-d_6 (0.2 mL, 2.8 mmol) was added to an oven-dried NMR tube. After 96 h at room temperature, the volatile components were removed under reduced pressure. The residue was washed with 2x3 mL of *n*-pentane, and the *n*-pentane fractions were collected. The combined pentane fractions were then evaporated under reduced pressure. The residue was dissolved in 0.4 mL of chloroform, and the volatile components were again removed under reduced pressure to afford anthracene crystals. These crystals were gently washed three times with 0.3 mL of cold *n*-pentane. Finally, all volatile materials were removed under reduced pressure, yielding a pale-yellow solid with an 86% NMR yield. The product was crystallized from *n*-pentane at -35 °C.

^{1}H-NMR (300 MHz, benzene-d_6) δ (ppm): 0.14 (s, 36H), 4.17 (s, 2H), 7.04 (dd, J = 5.6, 3.3 Hz, 4H),, 7.26 (dd, J = 5.5, 3.3 Hz, 4H)
^{13}C{^{1}H}-NMR (125 MHz, $CDCl_3$) δ (ppm): 138.0, 126.2, 125.5, 48.4, 3.3.
^{11}B-NMR (96 MHz, benzene-d_6) δ (ppm): 34.75 (s).
MS (TOF) m/z (M^+) calcd for $C_{26}H_{47}B_2N_2OSi_4$: 537.2873, found: 537.2948.

Crystallographic data for **3c**

Crystal data: $C_{26}H_{46}B_2N_2OSi_4$, colourless block, 0.098 × 0.135 × 0.162mm^3, Monoclinic $P2_1$/c a=13.3475(2)Å, b=11.6494(2)Å, c=20.7903(4)Å, α=90°, β=99.908(2)°, γ=90°, from 19313 reflections, T=100.0(2)K, V=3184.5(1)Å^3, Z=4, Fw=536.63, Dc=1.12 Mg·m^{-3}, μ=0.208 mm^{-1}.

Data collection and processing: Rigaku Synergy R diffractometer equipped with HyPix-Arc 100° detector, MoKα (λ=10.71073Å), -20≤h≤20, -18≤k≤18, -29≤l≤30, 46335 reflections collected, 10940 independent reflections (R-int =0.0316). The data were processed with CrysAlis^{PRO}.

Solution and refinement: Structure solved with the SHELXT program. Full matrix least-squares refinement based on F^2 with SHELXL on 328 parameters with 3 restraints gave final R_1= 0.0382 (based on F^2) for data with I>2σ(I), wR_2= 0.0992 on 10940 reflections, and final R_1= 0.0551 (based on F^2), wR_2= 0.1061 for all data, goodness-of-fit on F^2 = 1.056. The largest electron density peak is 0.45 e·Å^{-3}. Largest hole -0.24 e·Å^{-3}.

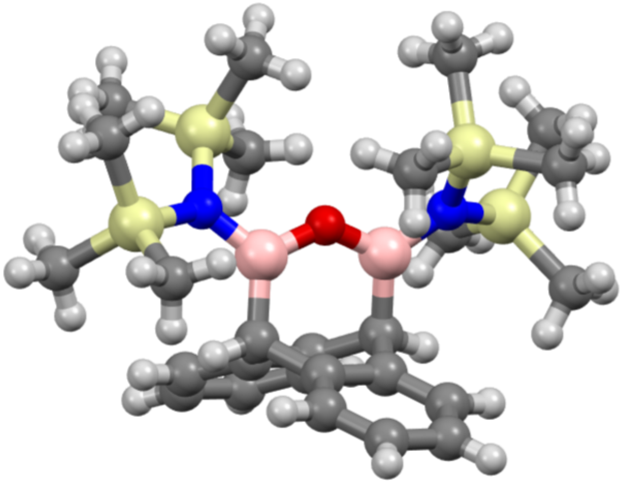

Insertion of oxoborane into **1d** (product **3d**)

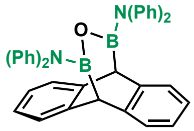

In a nitrogen-filled glovebox, a solution of $(Ph)_2N$-BA (**1d**) (100 mg, 0.28 mmol) in 0.4 mL of benzene-d_6 and DMSO-d_6 (0.2 mL, 2.8 mmol) was added to an oven-dried NMR tube. After 8 hours at room temperature, the volatile components were removed under reduced pressure. The residue was washed with 3 mL of *n*-pentane, and the *n*-pentane fractions were collected. Due to the instability challenges of **3d**, we could not isolate the product. Its formation and yield were confirmed through crude analysis of ^{11}B-NMR and ^{1}H-NMR spectra.

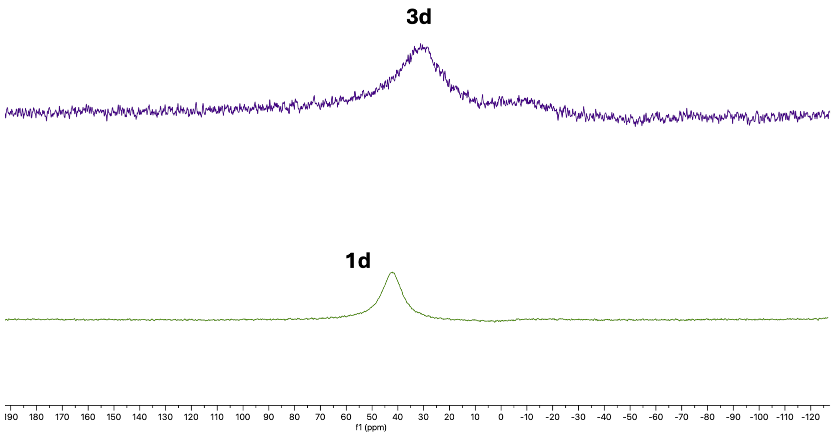

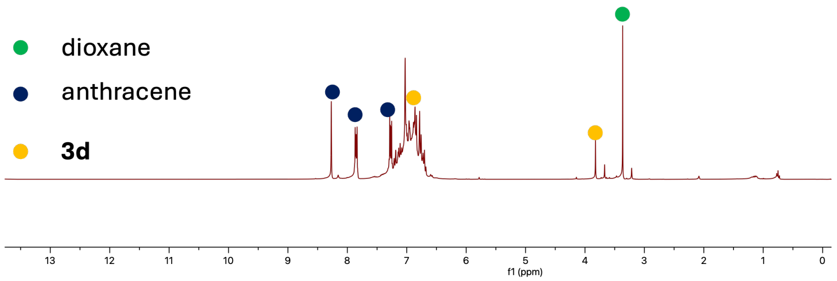

Trapping of TMS-oxoborane by *N*-*tert*-butyl-1-(3-nitrophenyl) nitrone (**5a**)

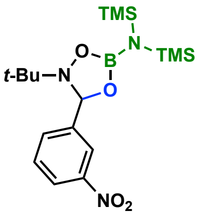

In a nitrogen-filled glovebox, a solution of TMS-BA (**1c**) (100 mg, 0.28 mmol) and *N*-*tert*-butyl-1-(3-nitrophenyl) nitrone (62 mg, 0.28 mmol) in 0.5 mL of benzene-d_6 and 0.25 mL of DMSO-d_6 was added to an oven-dried Young NMR tube. After 3 h at 80 °C, the volatile components were removed under reduced pressure. The work-up involved washing the residue with 3 mL of *n*-pentane twice, collecting the *n*-pentane fraction each time, and then removing the volatile materials under reduced pressure. The residue was dissolved in 2 mL of chloroform, and the volatile components were removed again under reduced pressure to form anthracene crystals. These crystals were gently washed twice with 1 mL of cold *n*-pentane. Finally, all volatile materials were removed under reduced pressure, yielding a yellow oil (70 mg, 0.17 mmol, 60%), which was crystallized at room temperature to form yellow crystals.

^{1}H-NMR (400 MHz, $CDCl_3$) δ (ppm): 8.37 (s, 1H), 8.20 (d, *J* = 8.2 Hz, 1H), 7.84 (d, *J* = 7.6 Hz, 1H), 7.56 (t, *J* = 8.0 Hz, 1H), 5.90 (s, 1H), 1.22 (s, 9H), 0.24 (s, 16H).
^{13}C{^{1}H}-NMR (101 MHz, $CDCl_3$) δ (ppm): 143.8, 132.4, 129.4, 125.3, 123.2, 121.5, 87.8, 59.5, 25.4, 3.4.
^{11}B-NMR (96 MHz, benzene-d_6) δ (ppm): 26.5 ppm (br s).
MS (TOF) m/z calcd for $C_{17}H_{32}BN_3O_4Si_2$: 410.2107, found: 410.1878.

Crystallographic data for **5a**

Crystal data: $C_{17}H_{32}BN_3O_4Si_2$, Yellow plate, 0.104 x 0.038 x 0.023 mm^3, Triclinic *P-1*, a=9.0557(2)Å, b=11.0745(3)Å, c=12.1768(3)Å, α=85.274(2)°, β=79.323(2)°, γ=71.843(2)°, from 47365 reflections, T=100(2)K, V=1139.89(5)$Å^3$, Z=2, Fw=409.44, Dc=1.193 $Mg{\cdot}m^{-3}$, μ=1.626 mm^{-1}.

Data collection and processing: Rigaku Synergy R dual source diffractometer equipped with Hypix-Arc 150 detector, CuKα (λ=1.54184Å), -11≤h≤11, -13≤k≤13, -14≤l≤14, frame scan width = 0.25°, scan speed 1.0° per 0.4 sec for low resolution and 1.60 sec for high resolution, 47365 reflections collected, 4285 independent reflections (R_{int} =0.0574). The data were processed with CrysAlis^{PRO}.

Solution and refinement: Structure solved with the SHELXT program. Full matrix least-squares refinement based on F^2 with SHELXL on 253 parameters with no restraints gave final R_1= 0.0451 (based on F^2) for data with I>2σ(I), wR_2= 0.1143 on 4285 reflections, and final R_1= 0.0639 (based on F^2), wR_2= 0.1244 for all data, goodness-of-fit on F^2 = 1.046 largest electron density peak 0.39 $e{\cdot}Å^{-3}$. Largest hole -0.29 $e{\cdot}Å^{-3}$.

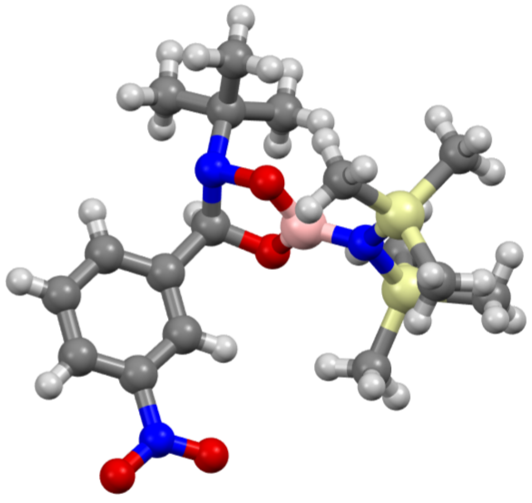

Trapping of TMS-oxoborane by *t*-buty phenyl nitrone (**5b**)

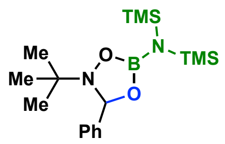

In a nitrogen-filled glovebox, a solution of TMS-BA (**1*c***) (311 mg, 0.89 mmol) and *N-tert*-butyl-α-phenylnitrone (PBN) (155 mg, 0.88 mmol) in 2.4 mL of benzene and 0.6 mL of DMSO was added to an oven-dried vial containing a stir bar. The mixture was stirred at 50 °C for 24 h. Afterward, the volatile components were removed under reduced pressure. The residue was washed with 5 mL of *n*-pentane twice. The combined *n*-pentane fractions were evaporated under reduced pressure. The residue was then dissolved in 2 mL of chloroform, and the volatile materials were removed under reduced pressure to form anthracene crystals. These crystals were gently washed twice with 3 mL of cold *n*-pentane. Finally, all volatile materials were removed under reduced pressure, yielding 206 mg (0.545 mmol, 61%) of a colorless oil.

^{1}H-NMR (300 MHz, $CDCl_3$) δ (ppm): 7.52-7.24 (m, 5H), 5.81 (s, 1H), 1.18 (s, 9H), 0.22 (s, 18H).
^{13}C{^{1}H}-NMR (125 MHz, $CDCl_3$) δ (ppm): 141.7, 128.5, 128.3, 126.4, 89.2, 59.4, 25.6, 3.6.
^{11}B-NMR (96 MHz, benzene-d_6) δ (ppm): 26.6 (s).
MS (TOF) m/z calcd for $C_{17}H_{33}BN_2O_2Si_2$: 365.2174, found: 365.2174.

Trapping of ^{i}Pr-oxoborane by *t*-buty phenyl nitrone (**5c**)

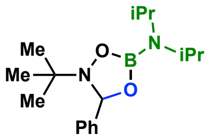

In a nitrogen-filled glovebox, a solution of ^{i}Pr-BA (**1a**) (162 mg, 0.58 mmol) and PBN (103 mg, 0.58 mmol) in 1.2 mL of benzene-d_6 and 0.4 mL of DMSO-d_6 was added to an oven-dried in 4 mL syn-vial. After 4 h at 90 °C, the volatile materials were removed under reduced pressure. At this stage, an NMR yield of 61% was determined using dioxane as an internal standard. The residue was washed twice with 3 mL of *n*-pentane, and the volatile components were removed under reduced pressure. The residue was dissolved in 0.4 mL of $CDCl_3$, and the volatile materials were removed again under reduced pressure to form anthracene crystals and the dissolved product. After filtration, all volatile materials were removed under reduced pressure, yielding a yellow liquid (52 mg, 0.40 mmol, 29%).

^{1}H-NMR (400 MHz, $CDCl_3$) δ (ppm): 7.51 – 7.27 (m, 5H), 5.88 (s, 1H), 3.52 – 3.42 (m, 2H), 1.24 (s, 9H), 1.19 (t, J = 6.7 Hz, 12H).
^{13}C{^{1}H}-NMR (101 MHz, $CDCl_3$) δ (ppm): 130.1, 128.8, 128.4, 128.3, 125.8, 88.6, 59.5, 45.5, 28.4, 25.7 ppm.
^{11}B-NMR (96 MHz, benzene-d_6) δ (ppm): 25.60 (s) ppm.

Trapping of TMS-oxoborane by (*S*)-4-(*tert*-butyl)-5-(3-iodophenyl) nitrone (**S2**)

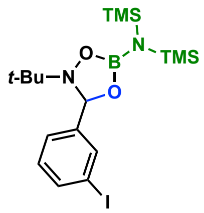

In a nitrogen-filled glovebox, a solution of TMS-BA (**1c**) (106 mg, 0.30 mmol) and 4-(*tert*-butyl)-5-(3-iodophenyl) nitrone (92 mg, 0.30 mmol) in 0.8 mL of benzene and 0.2 mL of DMSO-d_6 was added to an oven-dried Young NMR tube. After 4 h at 70 °C, the volatile components were removed under reduced pressure. The residue was washed with 3 mL of *n*-pentane twice, followed by removing the volatile materials from the combined fractions under reduced pressure. The residue was then dissolved in 2 mL of chloroform, and the volatile components were removed under reduced pressure to form anthracene crystals. These crystals were gently washed twice with 1 mL of cold *n*-pentane. Finally, all volatile materials were removed under reduced pressure, yielding a colorless oil (40 mg, 0.08 mmol, 27%).

^{1}H-NMR (400 MHz, $CDCl_3$) δ (ppm): 7.78 (m, 1H), 7.66 (d, *J* = 7.8 Hz, 1H), 7.44 (d, *J* = 7.7 Hz, 1H), 7.11 (t, *J* = 7.8 Hz, 1H), 5.76 (s, 1H), 1.19 (s, 9H), 0.23 (s, 18H).
^{13}C{^{1}H}-NMR (101 MHz, $CDCl_3$) δ (ppm): 143.8, 137.2, 135.4, 130.1, 125.5, 94.3, 88.1, 59.4, 25.4, 3.4.
^{11}B-NMR (96 MHz, benzene-d_6) δ (ppm): 26.7 ppm (br s).
MS (TOF) m/z calcd $C_{17}H_{32}BIN_2O_2Si_2$: 491.1222, found: 491.0918.

Trapping of TMS-oxoborane by (*E*)-2-(*tert*-butyl)-3-styryl-1,2-oxaziridine (**6a**)

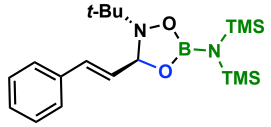

In an N_2-filled glovebox, a screw-capped vial (sealed) was charged with TMS-boranoanthacene **1c** (0.1 mmol, 1 equiv, 35 mg) and (*E*)-2-(*tert*-butyl)-3-styryl-1,2-oxaziridine **6** (0.12 mmol, 1.2 equiv, 24 mg) in 0.3 mL of benzene-d_6. To this solution, 0.1 mL of DMSO-d_6 was added and heated at 80 °C for 3 h till the full consumption of the starting material (monitored by ^{1}H-NMR). After completion of the reaction, the liquid reaction mixture was separated from the solid anthracene by a syringe. The solution was diluted with 3 mL *n*-pentane and was separated from the DMSO layer with a syringe. Next, the *n*-pentane layer was concentrated under reduced pressure to get yellowish oil product **6a** with a 41% NMR yield.

^{1}H-NMR (300 MHz, benzene-d_6) δ (ppm): 7.16 (dd, *J* = 7.8, 1.6 Hz, 2H), 7.0 (m, 3H), 6.66 (br d, *J* = 15.9 Hz, 1H), 6.29 (dd, 5.29 *J* = 6.0, 15.9 Hz, 1H), (dd, *J* = 6.0, 0.7 Hz, 1H), 1.07 (s, 9H), 0.35 (s, 18H).

^{13}C{^{1}H}-NMR (75 MHz, benzene-d_6) δ (ppm): 136.7, 131.8, 129.3, 128.8, 128.1 127.1, 89.4, 59.0, 25.5, 3.7.

^{11}B-NMR (96 MHz, benzene-d_6) δ (ppm): 26.3 ppm (br s).

MS (TOF) m/z calcd $C_{17}H_{32}BN_2O_2Si_2$: 391.2408, found: 391.2408.

Trapping of TMS-oxoborane by azomethine imine (**7a**)

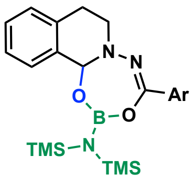

In an N_2-filled glovebox, a screw-capped vial (sealed) was charged with TMS-boranoanthacene **1c** (0.34 mmol, 1 equiv, 120 mg), azomethine imine **7** (0.35 mmol, 1.03 equiv, 132 mg), 1 mL of benzene-d_6, and 0.4 mL DMSO-d_6. This reaction mixture was heated to 50 ^{o}C for 15 h till the full consumption of the starting material (monitored by ^{1}H-NMR). After completion, the liquid mixture was separated from the solid anthracene using a syringe. The liquid crude solution was diluted with 5 mL *n*-pentane and separated from the DMSO layer with a syringe. Next, the *n*-pentane fraction was concentrated under reduced pressure to get the yellowish oil product **7a** in 57% NMR yield (as a mixture with starting material **7**).

^{1}H-NMR (500 MHz, benzene-*d6*) δ (ppm): 7.51 (d, *J* = 7.5 Hz, 1H), 7.14 (s, 1H), 7.07 (m, 1H), 7.02 (m, 1H), 6.83 (d, *J* = 7.5 Hz, 1H), 6.05 (s, 1H), 3.5 (m , 2H, C*H*(CH_3)$_2$ signal overlapping with C*H*$_2$ signal), 3.42 (m, 2H, C*H*$_2$ signal overlapping with C*H*(CH_3)$_2$ signal), 3.30 (dt, *J* = 11.8, 2.5 Hz, 1H), 3.10 (m, 1H), 2.77 (hep, *J* = 6.9 Hz, 1H), 2.34 (m, 1H), 1.36 (d, *J* = 6.9 Hz, 6H), 1.34 (d, *J* = 6.9 Hz, 6H), 1.18 (d, *J* = 6.9 Hz, 6H), 0.19 (s, 18H).
^{13}C{^{1}H}-NMR (125.7 MHz, benzene-*d6*) δ (ppm): 152.2, 150.1, 135.8, 135.6, 130.5, 128.8, 128.6, 128.1, 128.0, 126.7, 126.6, 125.6, 120.9, 91.7, 53.7, 34.8, 31.5, 28.9, 24.7, 24.2, 3.6.
^{11}B-NMR (96 MHz, benzene-d_6) δ (ppm): 26.3 ppm (s).

Crystallographic data for **7a**

Crystal data: $C_{31}H_{51}BN_3O_2Si_2$, light yellow needle, 0.13 x 0.06 x 0.03 mm^3, Orthorhombic *Pbca*, a=12.8020(3)Å, b=11.8710(4)Å, c=44.3475(15)Å, α=90°, β=90°, γ=90°, from 12970 reflections, 2Θ range for data collection 3.987° to 42.074°, T=100(2)K, V=6739.6(4)$Å^3$, Z=8, Fw=564.73, Dc=1.113 $Mg{\cdot}m^{-3}$, μ=1.179 mm^{-1}.

Data collection and processing: Rigaku Synergy R diffractometer equipped with Hypix-Arc 150 detector, CuKα (λ=1.54184Å), -10≤h≤11, -9≤k≤10, -38≤l≤38, frame scan width = 0.5°, scan speed 1.0° per 16 sec, 12970 reflections collected, 2318 independent reflections (R-int =0.0457). The data were processed with $CrysAlis^{PRO}$.

Solution and refinement: Structure solved with SHELXT program. Full matrix least-squares refinement based on F^2 with SHELXL on 340 parameters with no restraints gave final R_1= 0.1036 (based on F^2) for data with I>2σ(I), wR_2= 0.2726 on 2318 reflections, and final R_1= 0.1139 (based on F^2), wR_2= 0.2793 for all data, goodness-of-fit on F^2 = 1.181 largest electron density peak 0.868 $e{\cdot}Å^{-3}$. Largest hole -0.348 $e{\cdot}Å^{-3}$.

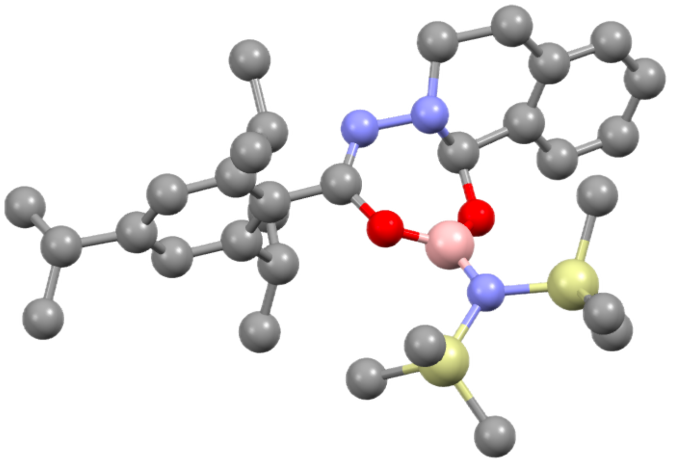

Insertion of 5-*tert*-butoxycarbonyl 5-methyl-1-pyrroline *N*-oxide into TMS-BA (**9**)

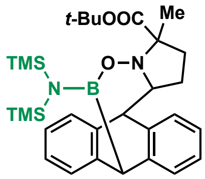

In a nitrogen-filled glovebox, a solution of $(TMS)_2N$-BA (**1c**) (50 mg, 0.14 mmol) and 5-*tert*-butoxycarbonyl-5-methyl-1-pyrroline-*N*-oxide (BMPO) (28 mg, 0.14 mmol) in 0.4 mL was added to an oven-dried NMR tube. After 8 days at 50 ºC, the volatile components were removed under reduced pressure. The work-up involved washing the residue with 3 mL of *n*-pentane three times, collecting the *n*-pentane fraction each time, and removing the volatile materials under reduced pressure. The residue was then dissolved in 0.4 mL of $CDCl_3$, and the volatile components were removed again under reduced pressure to form anthracene crystals. These crystals were gently washed twice with 0.3 mL of cold *n*-pentane. Finally, all volatile materials were removed under reduced pressure, resulting in the product. The product was crystallized in ether at -35 °C.

^{1}H-NMR (500 MHz, $CDCl_3$) δ (ppm): 7.35 (d, *J* = 7.3 Hz, 1H), 7.29 (t, *J* = 6.6 Hz, 2H), 7.23 – 7.09 (m, *J* = 13.6, 6.8 Hz, 5H), 4.08 (s, 1H), 4.05 (s, 1H), 3.99 (dd, *J* = 10.6, 4.3 Hz, 1H), 2.59 – 2.42 (m, 1H), 1.85 – 1.76 (m, 1H), 1.62 – 1.53 (m, 1H), 1.42 (s, 9H), 1.03 – 0.94 (m, 1H), 0.89 (s, 3H), 0.46 (s, 9H), -0.21 (s, 9H).
^{13}C{^{1}H}-NMR (126 MHz, $CDCl_3$) δ (ppm): 173.8, 142.9, 141.1, 138.6, 136.7, 128.3, 128.2, 127.6, 126.3, 126.1, 125.8, 125.7, 125.5, 125.2, 124.5, 80.8, 71.6, 68.5, 54.8, 32.1, 28.4, 28.3, 22.1, 4.4, 2.6.
^{11}B-NMR (96 MHz, benzene-*d*$_6$) δ (ppm): 37.8 (s).

Crystallographic data for **9**

Crystal data: $C_{30}H_{45}BN_2O_3Si_2$, colorless chunk, 0.141 x 0.108 x 0.103 mm^3, Monoclinic *P2₁/c*, a= 17.2706(3)Å, b=15.6132(2)Å, c=11.8743(2)Å, α=90°, β=104.477(2)°, γ=90°, from 69947 reflections, T=100(2)K, V=3100.23(8)Å^3, Z=4, Fw=548.67, Dc=1.176 Mg·m^{-3}, μ=0.147 mm^{-1}.

Data collection and processing: Rigaku Synergy R dual source diffractometer equipped with Hypix-Arc 100 detector, MoKα (λ=0.71073Å), -23≤h≤23, -21≤k≤21, -16≤l≤16, frame scan width = 0.5°, scan speed 1.0° per 17.6 sec, 69947 reflections collected, 8340 independent reflections (R-int =0.0327). The data were processed with CrysAlis^{PRO}.

Solution and refinement: Structure solved with the SHELXT program. Full matrix least-squares refinement based on F^2 with SHELXL on 353 parameters with no restraints gave final R_1= 0.0397 (based on F^2) for data with I>2σ(I), wR_2= 0.0940 on 8340 reflections, and final R_1= 0.0488 (based on F^2), wR_2= 0.0979 for all data, goodness-of-fit on F^2 = 1.054 largest electron density peak 0.434 e·Å^{-3}. Largest hole -0.239 e·Å^{-3}.

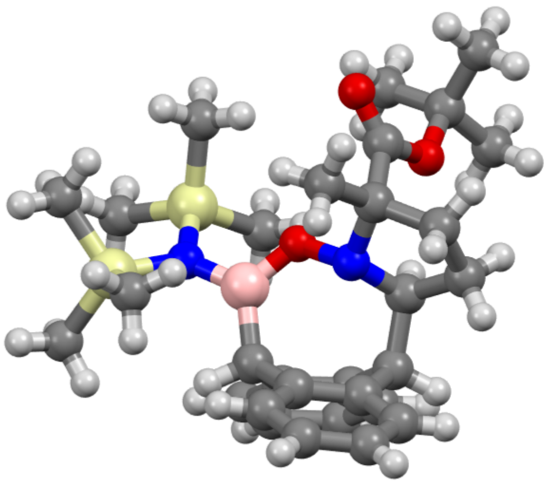

2. X-ray structure data

Table S1.

CIF file name	Compound 1d	Compound 1b	Compound 1c
**CCDC**	2389624	2389622	2389617
**Crystal description**	Yellow prism	Colorless needle	Colorless prism
**Source**	Rigaku Synergy R	Rigaku Synergy R	Rigaku Synergy R
**Empirical formula**	$C_{31}H_{25}BN_2$, 0.9[$CHCl_3$]	$C_{23}H_{28}BN$	$C_{20}H_{28}BNSi_2$
**Formula weight (g/mol)**	543.821	329.27	349.42
**Temperature (K)**	100(2)	100(2)	100(2)
**Wavelength (Å)**	0.71073	1.54184	0.71073
**Crystal system**	triclinic	orthorhombic	orthorhombic
**Space group**	*P-1*	$P2_12_12_1$	$P2_12_12_1$
**a (Å)**	9.2048(1)	8.7311(10)	9.25248(18)
**b (Å)**	10.6139(2)	14.2388(18)	14.5350(3)
**c (Å)**	14.5012(2)	14.994(2)	15.1098(3)
**α°**	69.968(1)	90	90
**β°**	88.080(1)	90	90
**γ°**	85.277(1)	90	90
**Volume (Å^3)**	1326.50(4)	1864.1(4)	2032.03(7)
**Z**	2	4	4
**Density calculated (Mg/m^3)**	1.092	1.173	1.142
**Absorption coefficient (mm^{-1})**	0.063	0.494	0.176
**F(000)**	565.456	712.0	752.0
**Theta range for data collection (°)**	2.67 to 30.51	4.28 to 47.22	2.58 to 30.50
**Reflection collected (Unique)**	65045(8070)	6892(1693)	24960(6208)
**R int**	0.0213	0.1156	0.0353
**Completeness %**	99.8	99.8	100.0
**Data\restraints\parameters**	8070 /50/ 307	1200 /0/230	6208/0/223
**Goodness-of-fit on F^2**	1.011	0.989	1.056
**Final R [I>2σ(I)]**	R1=0.0433 wR2= 0.1202	R1=0.0770 wR2= 0.1753	R1=0.0333 wR2= 0.0831
**R (all data)**	R1=0.0520 wR2=0.1268	R1=0.1170 wR2=0.1991	R1=0.0388 wR2=0.0858
**Largest diff. peak and hole (e$^-$ Å^{-3})**	0.394 and -0.240	0.252 and -0.196	0.262 and -0.227

Table S2.

CIF file name	Compound 5a	Compound 9	Compound 3c
CCDC	2389625	2389623	2389621
Crystal description	Yellow plate	Colorless prism	Colorless block
Source	Rigaku Synergy R	Rigaku Synergy R	Rigaku Synergy R
Empirical formula	$C_{17}H_{32}BN_3O_4Si_2$	$C_{25}H_{33}BN_2OSi_2$	$C_{26}H_{46}B_2N_2OSi_4$
Formula weight (g/mol)	409.44	444.52	536.63
Temperature (K)	100(2))	100(2)	100(2)
Wavelength (Å)	1.54184	0.71073	0.71073
Crystal system	triclinic	monoclinic	monoclinic
Space group	*P*-1	*P*2$_1$/n	*P*2$_1$/c
a (Å)	9.0557(2)	12.8462(2)	13.3475(2)
b (Å)	11.0745(3)	13.1890(2)	11.6494(2)
c (Å)	12.1768(3)	15.2059(3)	20.7903(4)
α°	85.274(2)	90	90
β°	79.323(2)	107.2736(18)	99.9075(17)
γ°	71.843(2)	90	90
Volume ($Å^3$)	1139.89(5)	2460.13(7)	3184.50(9)
Z	2	4	4
Density calculated (Mg/m^3)	1.193	1.176	1.119
Absorption coefficient (mm^{-1})	1.162	0.147	0.208
F(000)	440.0	1184.0	1160.0
Theta range for data collection (°)	3.69 to 70.07	2.47 to 29.13	2.64 to 33.77
Reflection collected (Unique)	47365(4285)	108030(6615)	46335(10940)
R int	0.0343	0.0188	0.0316
Completeness %	99.2	99.9	85.7
Data\restraints\parameters	4285 /0/ 253	6615 /0/ 289	10940 /3/328
Goodness-of-fit on F^2	1.046	1.039	1.056
Final R [I>2σ(I)]	R1=0.0451 wR2= 0.1143	R1=0.0343 wR2= 0.0898	R1=0.0382 wR2= 0.0992
R (all data)	R1=0.0639 wR2=0.1244	R1=0.0440 wR2=0.0956	R1=0.0551 wR2=0.1061
Largest diff. peak and hole ($e^- Å^{-3}$)	0.387 and -0.290	0.418 and -0.181	0.452 and -0.243

Table S3.

CIF file name	Compound 1a	Compound 7	Compound 7a
**CCDC**	2389619	2422685	2422684
**Crystal description**	Colorless plate	Light yellow prism	light yellow needle
**Source**	Rigaku Synergy S	Rigaku Synergy R	Rigaku Synergy R
**Empirical formula**	$C_{20}H_{24}BN$	$C_{25}H_{32}N_2O$	$C_{31}H_{51}BN_3O_2Si_2$
**Formula weight (g/mol)**	289.21	376.52	564.73
**Temperature (K)**	99.98(10)	100.02(10)	100.00(10)
**Wavelength (Å)**	0.71073	0.71073	1.54184
**Crystal system**	monoclinic	monoclinic	orthorhombic
**Space group**	$P2_1/n$	$P2_1/n$	*Pbca*
**a (Å)**	9.4832(4)	10.6136(2)	12.8020(3)
**b (Å)**	12.5551(5)	13.4079(2)	11.8710(4)
**c (Å)**	14.4109(5)	15.3481(3)	44.3475(15)
**α°**	90	90	90
**β°**	92.244(4)	99.604(2)	90
**γ°**	90	90	90
**Volume (Å^3)**	1714.48(12)	2153.52(8)	6739.6(4)
**Z**	4	4	8
**Density calculated (Mg/m^3)**	1.120	1.161	1.113
**Absorption coefficient (mm^{-1})**	0.063	0.070	1.179
**F(000)**	624	816	2456
**Theta range for data collection (°)**	2.15 to 31.61	5.304 to 61.794	3.987 to 42.074
**Reflection collected (Unique)**	46818(5135)	41969(6011)	12970(2318)
**R int**	0.0501	0.0380	0.0457
**Completeness %**	89.3	99.9	99.9
**Data\restraints\parameters**	5135/0/ 203	6011/0/259	2318/0/340
**Goodness-of-fit on F^2**	1.026	1.034	1.181
**Final R [I>2σ(I)]**	R1=0.0501 wR2= 0.1131	R1=0.0496 wR2= 0.1349	R1=0.1036 wR2= 0.2726
**R (all data)**	R1=0.0754 wR2=0.1221	R1=0.0598 wR2=0.1427	R1=0.1139 wR2=0.2793
**Largest diff. peak and hole (e$^-$ Å^{-3})**	0.316 and -0.229	0.316 and -0.229	0.868 and -0.348

3. Additional data

Potential by-products obtained during boranoanthracene synthesis:

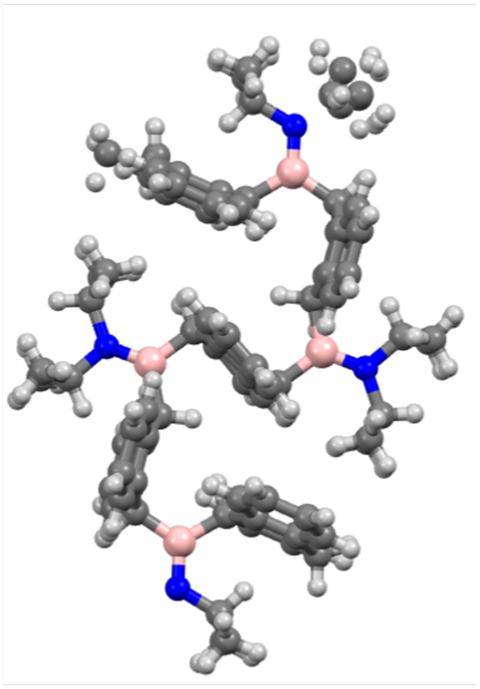

Figure S1: By-products of magnesium anthracene and dihaloboranes – oligomer of diborane-dihydroanthracene.

Crystallographic data for a cyclic boroxine trimer

Crystal data: $C_{36}H_{24}B_3N_9O_{15}$, yellow block, 0.152 x 0.073 x 0.047 mm^3, Monoclinic *C2/c*, a=11.4132(7)Å, b=26.1651(11)Å, c=12.4918(8)Å, α=90°, β=106.545(7)°, γ=90°, from 41795 reflections, T=100(2)K, V=3575.9(4)Å^3, Z=4, Fw=855.07, Dc=1.588 Mg·m^{-3}, μ=1.062 mm^{-1}.

Data collection and processing: Rigaku Synergy R dual source diffractometer equipped with Hypix-Arc 150 detector, CuKα (λ=1.54184Å), -14≤h≤14, -32≤k≤32, -15≤l≤15, frame scan width = 0.25°, scan speed 1.0° per 0.4 sec for low resolution and 1.40 sec for high

resolution, 41795 reflections collected, 3671 independent reflections (R_{int} =0.0445). The data were processed with CrysAlis^{PRO}.

Solution and refinement: Structure solved with the SHELXT program. Full matrix least-squares refinement based on F^2 with SHELXL on 296 parameters with 1 restraint gave final R_1= 0.0723 (based on F^2) for data with I>2σ(I), wR_2= 0.2176 on 3671 reflections, and final R_1= 0.0885 (based on F^2), wR_2= 0.2353 for all data, goodness-of-fit on F^2 = 1.025 largest electron density peak 0.77 e·Å^{-3}. Largest hole -0.51 e·Å^{-3}.

<u>Crystallographic data diborane-dihydroanthracene oligomer</u>

Crystal data: $C_{94.65}H_{108.65}B_4N_4$, colorless needle, 0.188 x 0.041 x 0.036 mm^3, Tetragonal $I4_1cd$, a=b=21.32398(17)Å, c=33.9955(5)Å, α=β=γ=90°, from 33565 reflections, T=100(2)K, V=15458.2(3)Å^3, Z=8, Fw=1345.59, Dc=1.156 Mg·m^{-3}, μ=0.488 mm^{-1}.

Data collection and processing: Rigaku Synergy R dual source diffractometer equipped with Hypix-Arc 150 detector, CuKα (λ=1.54184Å), -24≤h≤25, -18≤k≤21, -28≤l≤40, frame scan width = 0.25°, scan speed 1.0° per 52.0 sec for low resolution and 80.0 sec for high resolution, 33565 reflections collected, 6339 independent reflections (R-int =0.0214). The data were processed with CrysAlis^{PRO}.

Solution and refinement: Structure solved with the SHELXT program. Full matrix least-squares refinement based on F^2 with SHELXL on 482 parameters with 12 restraints gave final R_1= 0.0772 (based on F^2) for data with I>2σ(I), wR_2= 0.1897 on 6339 reflections, and final R_1= 0.0836 (based on F^2), wR_2= 0.1979 for all data, goodness-of-fit on F^2 = 1.056 largest electron density peak 0.636 e·Å^{-3}. Largest hole -0.346 e·Å^{-3}.

Stability study of **1a** under thermal conditions (70 °C) without DMSO:

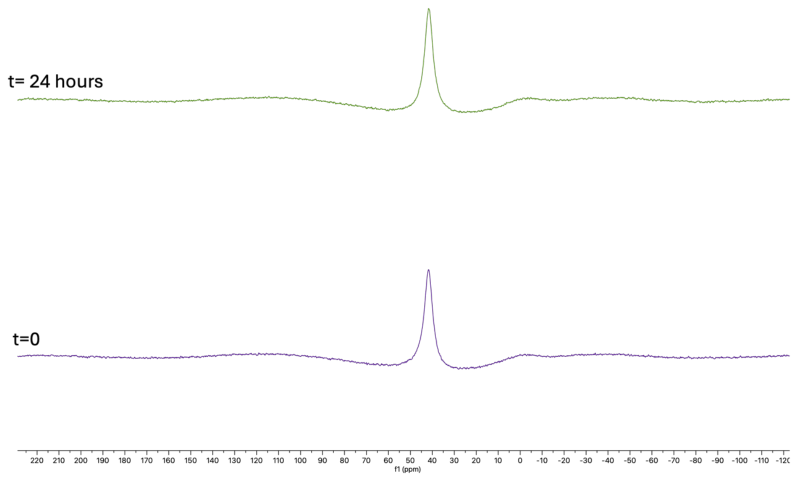

Figure S2: ^{11}B-NMR spectra of ^{i}Pr-BA **1a** (30 mg, 0.12 mmol) reaction in benzene (0.5 mL) at 70 °C.

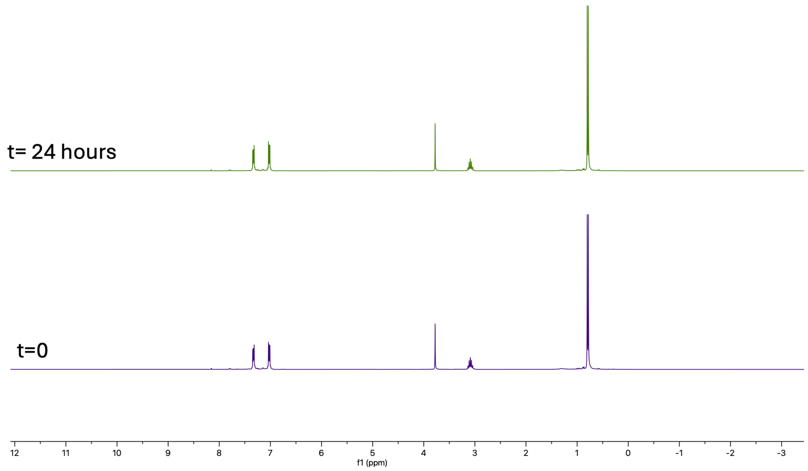

Figure S3: ^{1}H-NMR spectra of ^{i}Pr-BA **1a** (30 mg, 0.12 mmol) reaction in benzene (0.5 mL) at 70 °C.

<u>Reaction of **1a** with 20 eq of DMSO at 70 °C:</u>

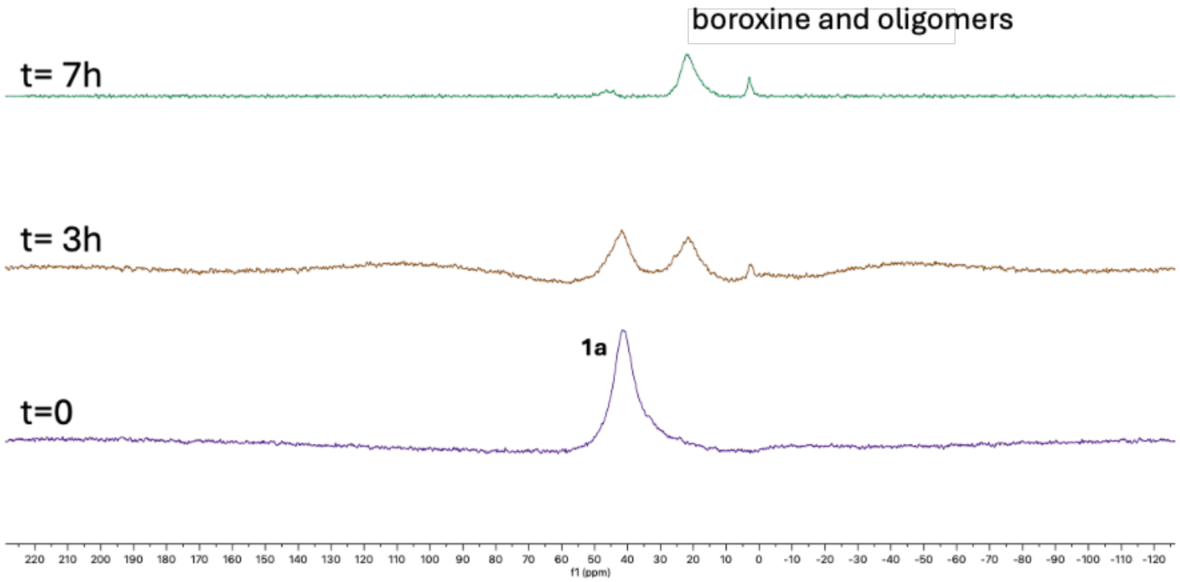

Figure S4: ^{11}B-NMR spectra of ^{i}Pr-BA **1a** (50 mg, 0.2 mmol) reaction with DMSO (20 eq.) in benzene (0.5 mL).

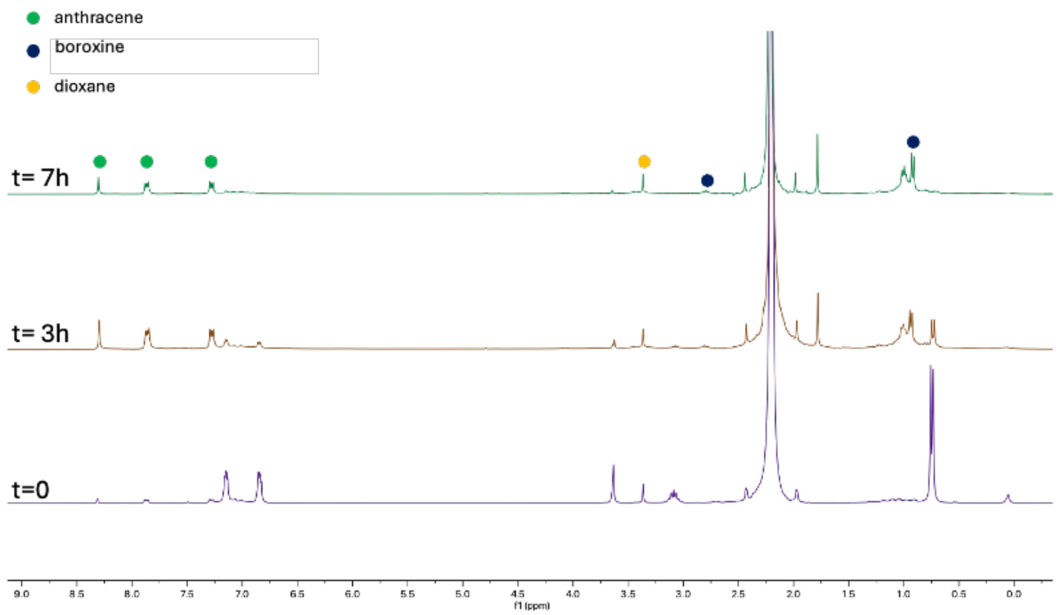

Figure S5: ^{1}H-NMR spectra of ^{i}Pr-BA **1a** (50 mg, 0.2 mmol) reaction with DMSO (20 eq.) in benzene (0.5 mL) and dioxane as internal standard.

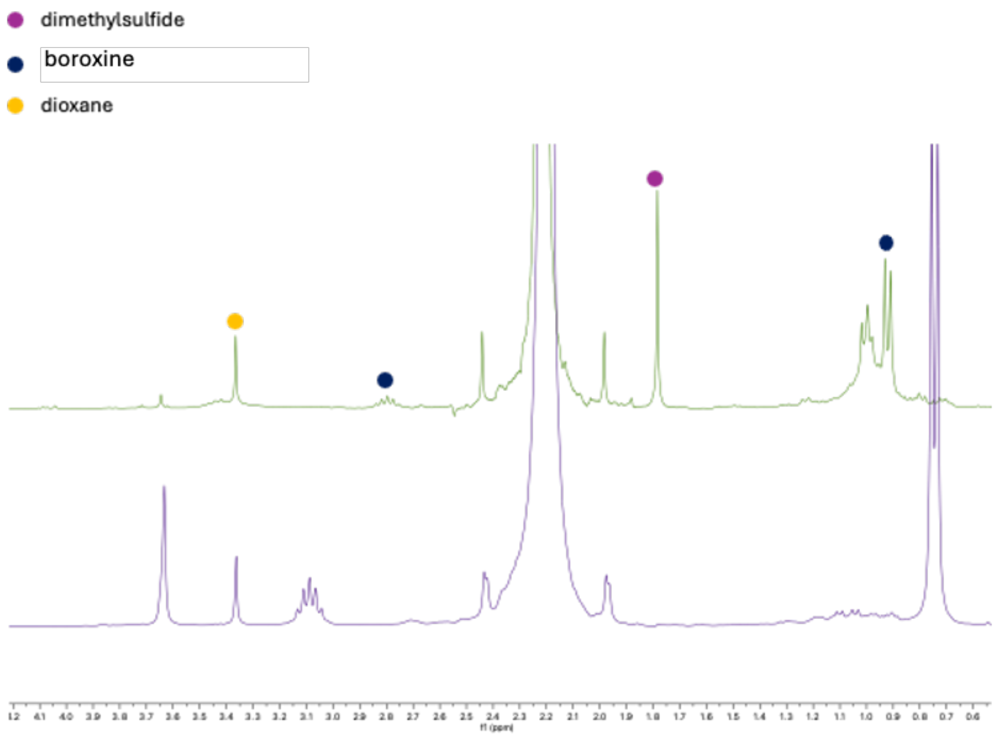

Figure S6: ^{1}H-NMR spectra of ^{i}Pr-BA **1a** (50 mg, 0.2 mmol) reaction with DMSO (20 eq.) in benzene (0.5 mL).

Reaction of **1b** with 20 eq of DMSO at 80 °C:

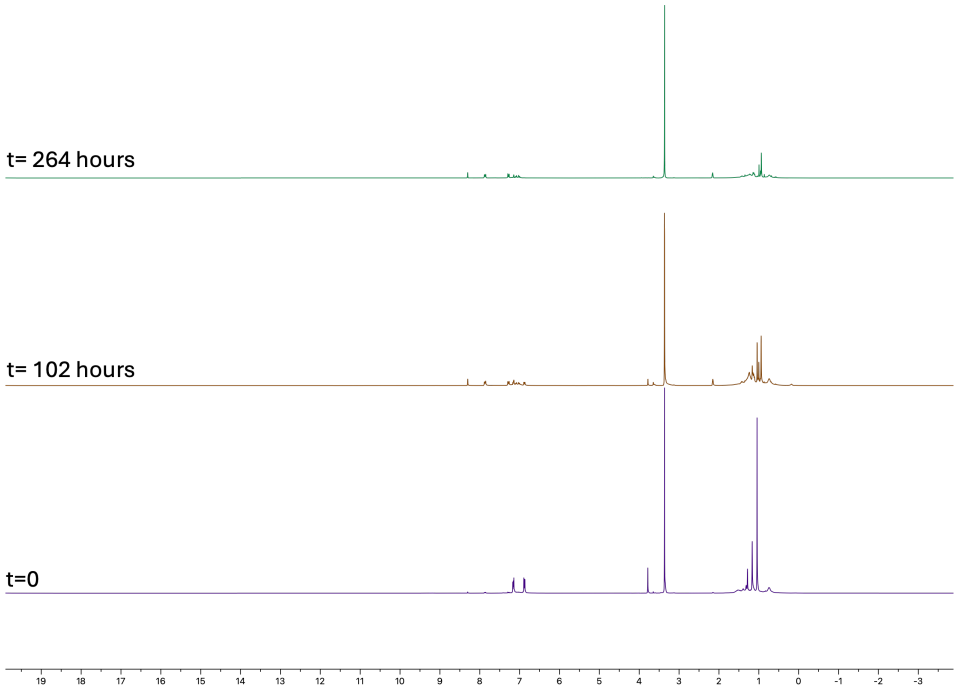

Figure S7: ^{1}H-NMR spectra of TMP-BA **1b** (50 mg, 0.2 mmol) reaction with DMSO (20 eq.) in benzene (0.5 mL).

Reaction of **1c** with 20 eq of DMSO at room temperature:

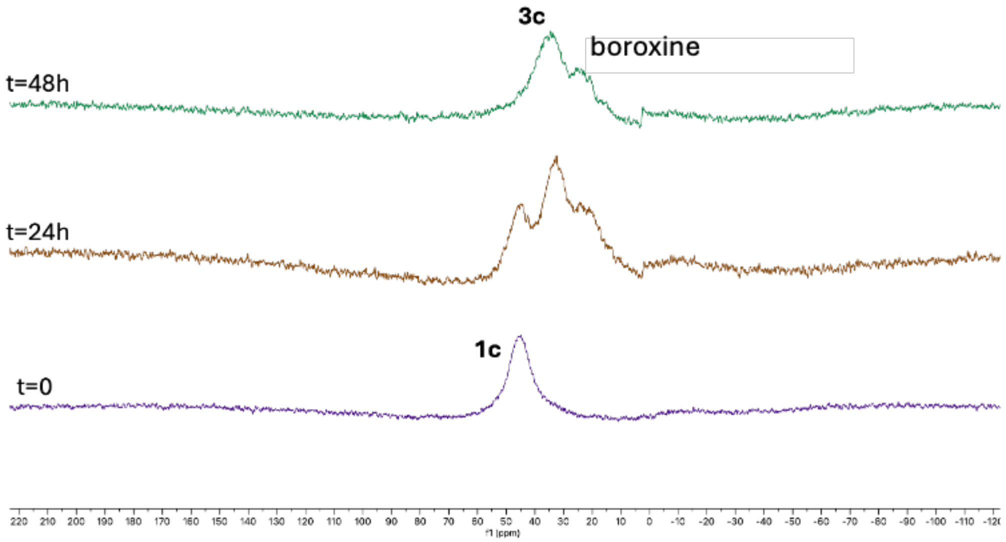

Figure S8: ^{11}B-NMR spectra of TMS-BA **1c** (50 mg, 0.2 mmol) reaction with DMSO (20 eq.) in benzene (0.5 mL).

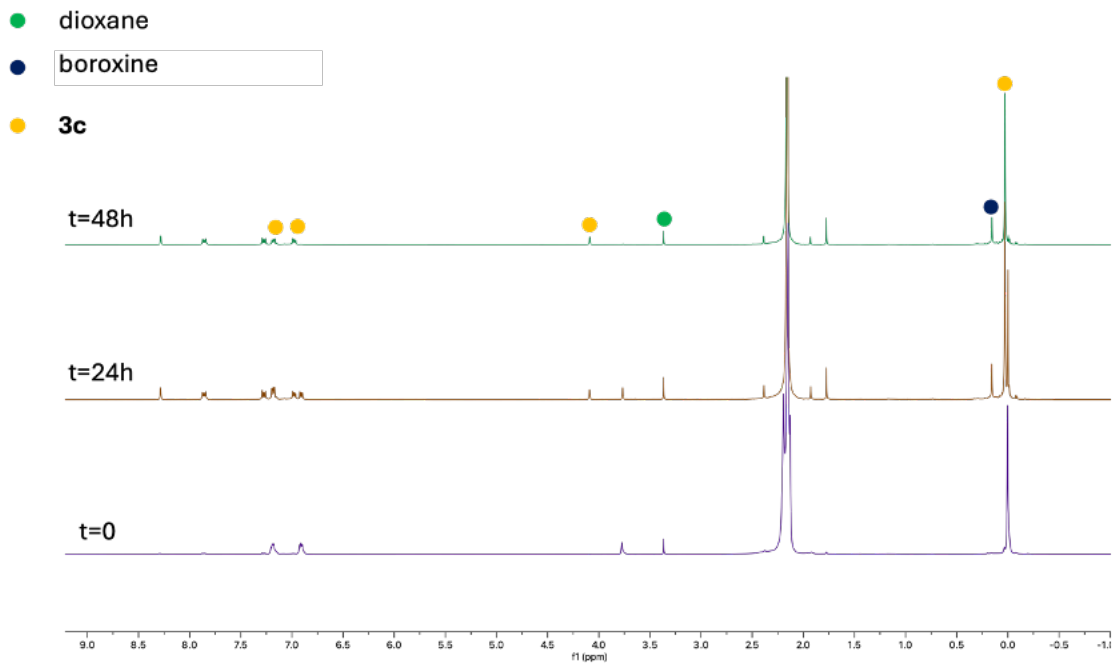

Figure S9: ^{1}H-NMR spectra of TMS-BA **1c** (50 mg, 0.2 mmol) reaction with DMSO (20 eq.) in benzene (0.5 mL).

Reaction of **1d** with 20 eq of DMSO at 70 °C:

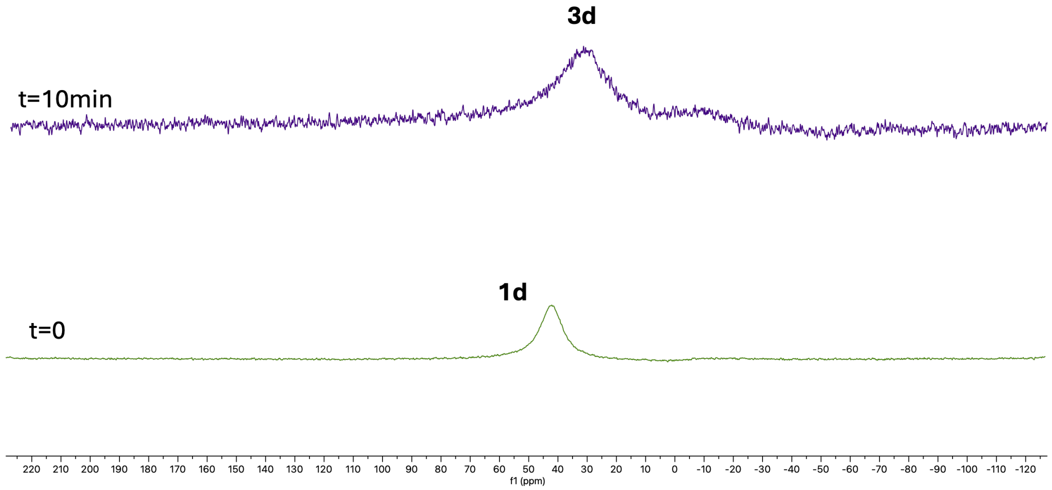

Figure S10: ^{11}B-NMR spectra of Ph-BA **1d** (50 mg, 0.2 mmol) reaction with DMSO (20 eq.) in benzene (0.5 mL).

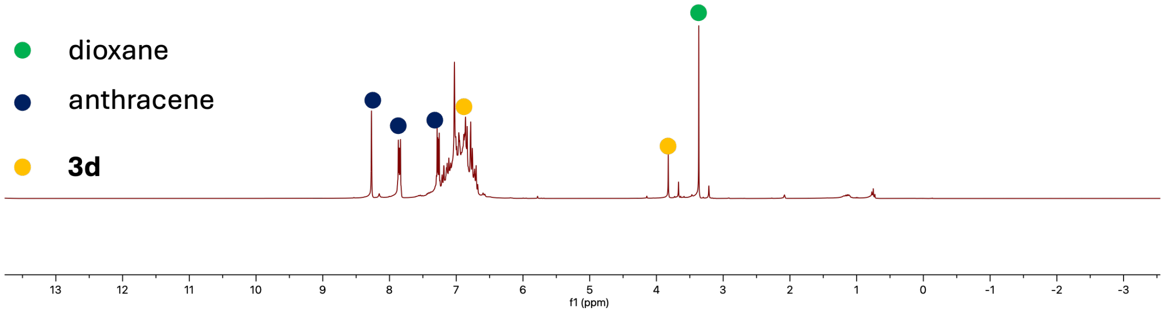

Figure S11: ^{1}H-NMR spectra of Ph-BA **1d** (50 mg, 0.2 mmol) reaction with DMSO (20 eq.) in benzene (0.5 mL).

In the cases of **1c** and **1d**, the faster fragmentation compared to **1a** can be attributed to the increased Lewis acidity of boron, which facilitates the chelation of DMSO. This effect presumably arises from the weaker π-back donation of the amine lone pair to the *p*-orbital of boron due to lower availability, as also demonstrated by the comparison of the B-N bond lengths in **1c** (1.395 Å) and **1a** (1.374 Å), as seen in the X-ray structures (Table S1-S3). Conversely, although the electronic characteristics of **1b** are presumably similar to those of **1a**, the prolonged reaction time of **1b** may be explained by the sterically crowded environment of TMP, which hinders DMSO coordination. Further study is required to assess how steric factors affect DMSO coordination efficiency.

Titration experiment of DMSO with Ph-BA (**1d**)

We performed a titration experiment by varying the amount of DMSO (5–100 eq) added to Ph-BA (Figure S12). In this experiment, the ^{11}B-NMR spectrum exhibited a significant shift, ranging from 40 ppm to 22 ppm, depending on the concentration of DMSO. This shift is attributed to the formation of varying amounts of the DMSO-coordinated adduct in the solution.

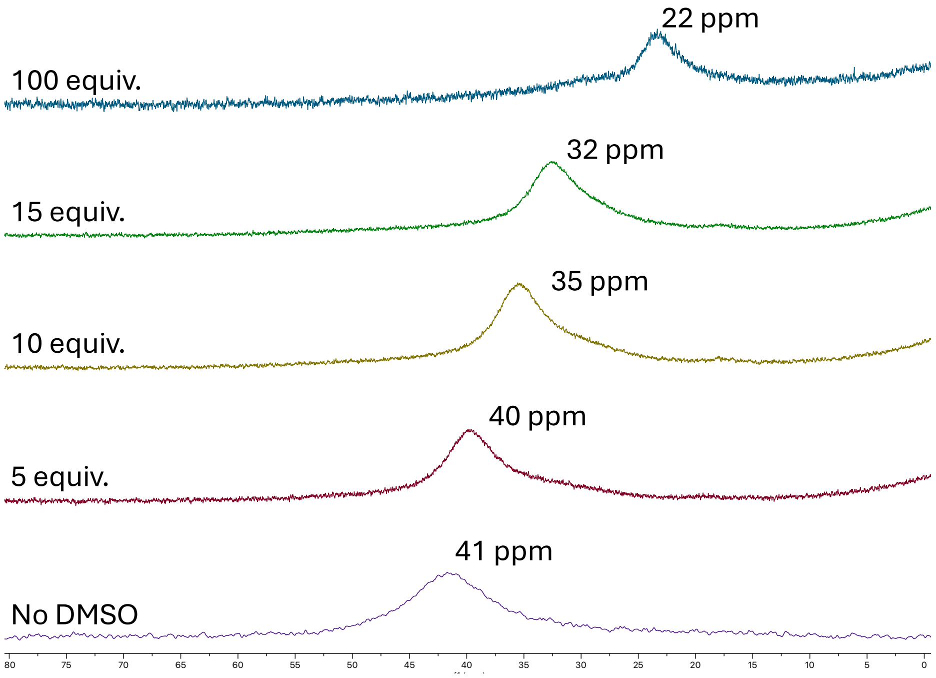

Figure S12: ^{11}B-NMR (128 MHz) titration experiment of DMSO (5-100 equiv.) to Ph-BA **1d** (15mg, 0.04 mmol), in 0.3 mL toluene-d_8 .

Diffusion NMR experiment:

The ^{1}H diffusion NMR experiments were recorded on an 11.75T (500.08 MHz) Bruker AVANCE III HD spectrometer equipped with a 50 gauss/cm Z gradient system. The measurements were performed at 298K using the bipolar stimulated echo pulse sequence (BPSTE) with smoothed square (SMSQ.10.100) gradients. The gradients were incremented from 2% to 98% in 10 linear steps, and 16 scans were acquired for each gradient. The gradient duration was 2 ms, and the diffusion time was 60 ms. Each diffusion measurement was performed 3 times consecutively, and an average with standard deviation was calculated and reported. To avoid the influence of temperature/viscosity/calibration of gradients, the diffusion coefficient of the benzene signal was measured and compared to its literature value at 298K. The differences between the measured value and the literature value were used as a factor, which was implemented in the calculation of the reported diffusion coefficients.

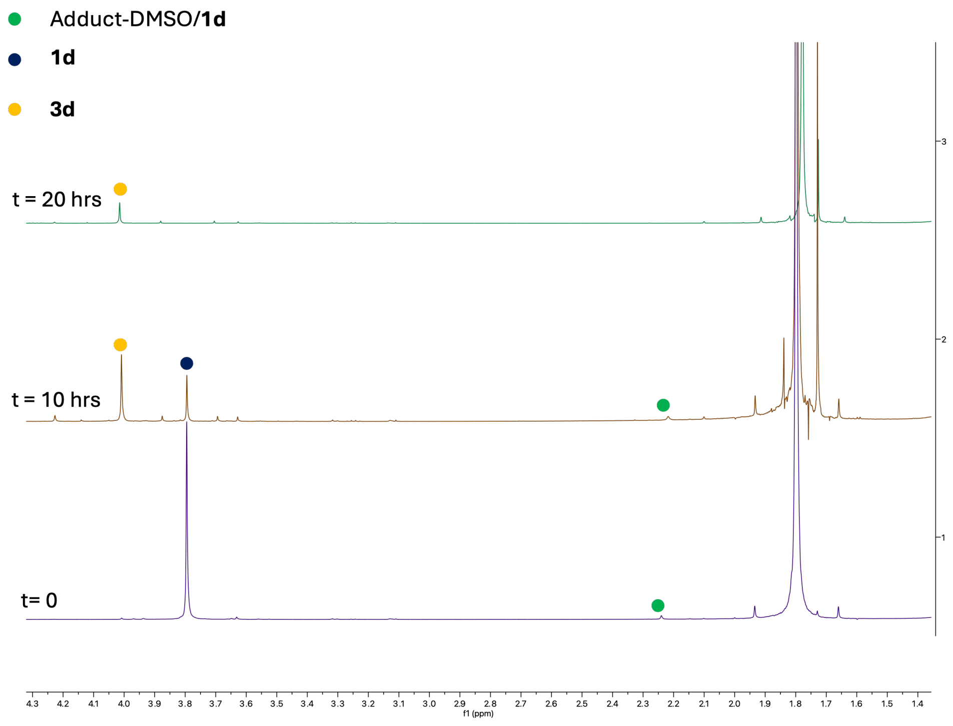

Figure S13: ^{1}H-NMR analysis of Ph-BA (**1d**) with 5 eq. of DMSO from t = 0 min (blue) to near full consumption t = 24 h (pink).

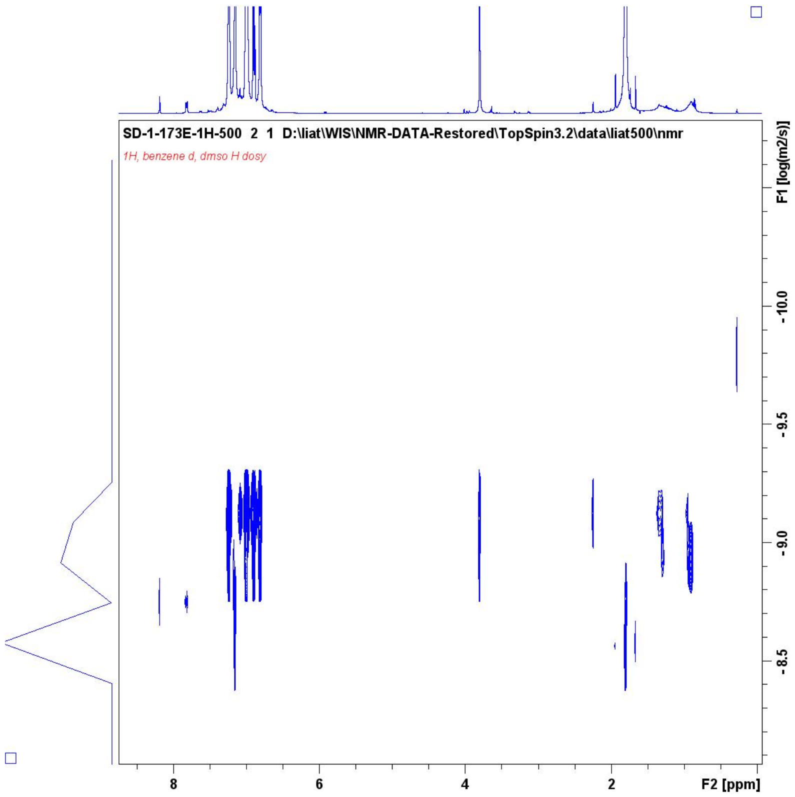

Figure S14: DOSY map of **1d**-DMSO adduct.

NOE experiment:

1-D selective ^{1}H NOE NMR (400.35MHz, 288K) spectrum was acquired with the standard Bruker pulse program, Selnogp, employing four gradients with 1 ms duration. To selectively irradiate the signal at 2.2 ppm, a Gauss1_180r.1000-shaped pulse was used and the SPOFFS was set to -714.15Hz. The mixing time was set to 400 ms, and a relaxation delay of 3 seconds with ns of 176 was employed. It is important to note that dipolar coupling may be due to NOE or chemical exchange. For small molecules, (positive NOE) opposite phases are observed, as expected, and the same phase is observed in the case of exchange.

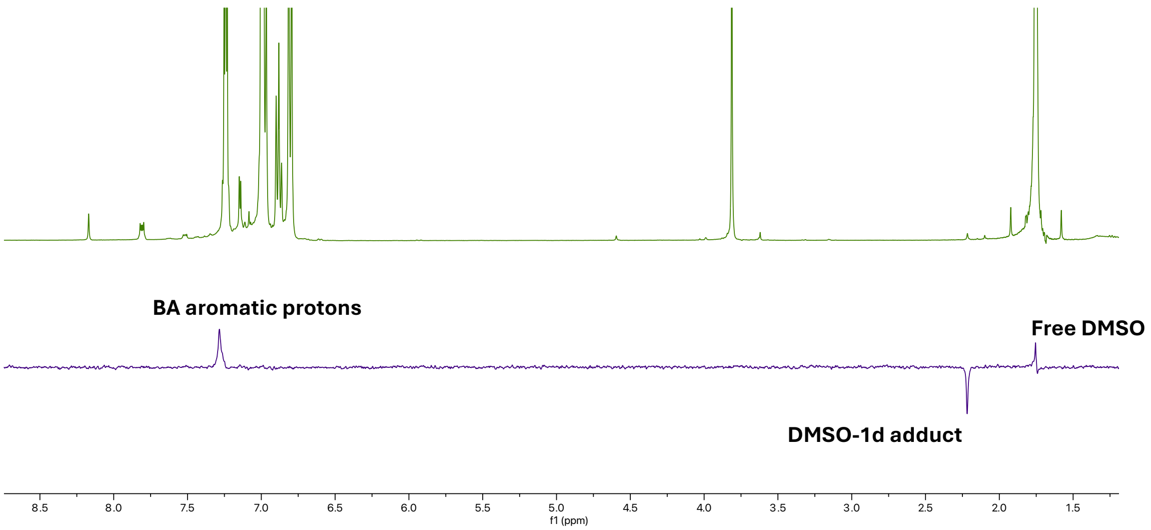

Figure S15: NOE experiment of **1d**-DMSO adduct. Conditions: Ph-BA (**1d**), DMSO (excess) in 0.4 mL of benzene-d_6, (288 K) 400 MHz NMR.

Control experiment of $PhSiH_3$ as an additive to **1c** and DMSO reaction:

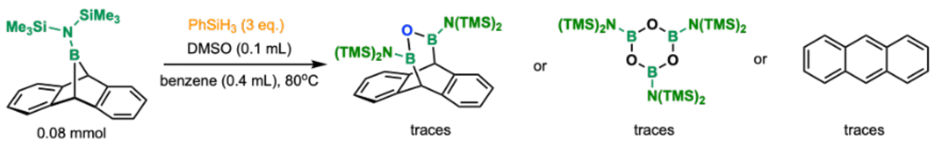

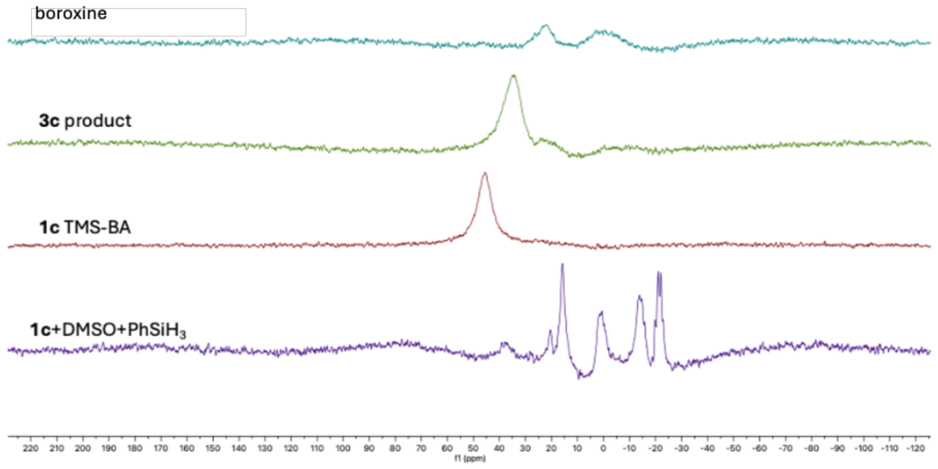

Figure S16: ^{11}B-NMR analysis of phenyl-silane as an additive to the **1c** reaction with DMSO.

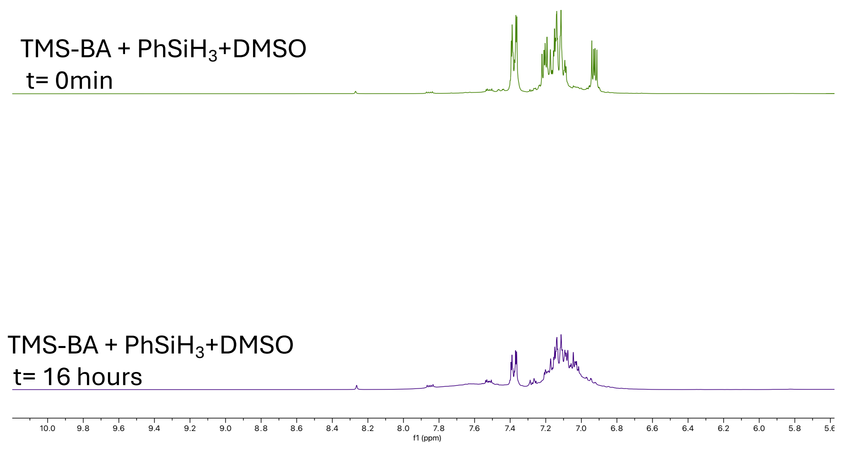

Figure S17: Reaction of TMS-BA with $PhSiH_3$ and DMSO.

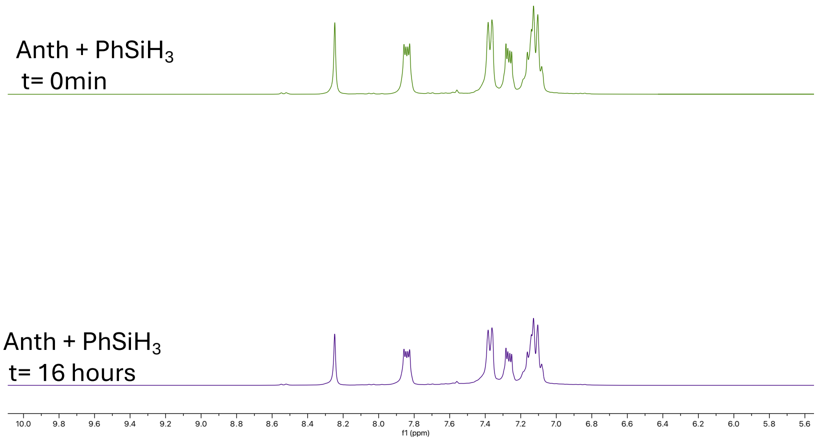

Figure S18: Reaction of Anthracene with $PhSiH_3$ and DMSO.

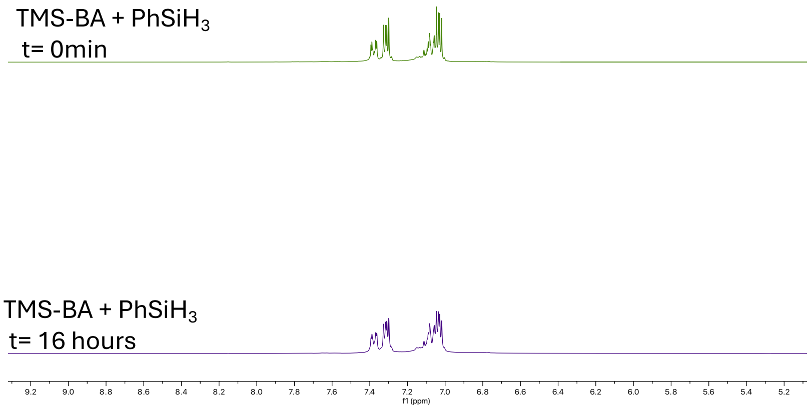

Figure S19: Reaction of TMS-BA with $PhSiH_3$ without DMSO.

4. Computational details

Method description:

Preliminary calculations used GAUSSIAN16 REV. C.01,[9] but the rest of the study relied on ORCA 5.0.4[10,11] (calculations on the reaction of the R_2NBO product with the nitrone added in review used ORCA 6.0.1). In this study, Adamo and Barone's hybrid version[12] of the Perdew–Burke–Ernzerhof exchange–correlation function[13,14] with Grimme's third version of his empirical dispersion correction[15] with Becke–Johnson dampening[16] (*i.e.,* PBE0-D3BJ) was used. With it, the augmented triple-ζ member of Weigend and Ahlrichs's "def2" basis sets (*i.e.,* def2-TZVPD) was used.[17,18] Density-fitting within the resolution of the identify–chain of spheres exchange (*i.e.*, RIJCOSX) was used to increase the computational efficiency of the calculations; this entailed the use of Weigend *et al.*'s def2/J[19] and Hellweg *et al.*'s def2-TZVPPD/C[20,21] auxiliary basis sets.

Solvation effects were implicitly modelled using a conductor-like polarizable continuum model (CPCM)[22,23] with benzene as the solvent.

Transition states were found either by optimizing a reasonable guess geometry or by using the nudged elastic band (NEB) method as described by Ásgeirsson et al.[24]; by default, ORCA starts a transition state geometry optimization starting from the NEB transition state. Minimum energy crossing points (MECP) were found using the method of Harvey et al.[25] as implemented in ORCA. As noted in the discussion of our DFT results, the MECP connecting **II** and **III** could not be found, likely due to the proximity of the corresponding MECP loss of **IV** and DMS; MECP(**III-IV**) results in a very low barrier. In this case, we resorted to a quasi-MECP approach where we performed a relaxed scan of the B–O bond length on both the singlet and triplet surfaces and crudely approximated the quasi-MECP point as the point where the energies of the two surfaces cross. The relaxed scan was done using Grimme and coworkers' GFN2-xTB[26] semiempirical method as implemented in their XTB code (version 6.6.1)[27]; during the relaxed scans (except for the **1c** singlet scan), a constraint was added to the second B–C bond (to prevent the initial step jumping to **II**) and to the B–O_{DMSO} bond (to prevent loss of DMSO). Accurate energies, including frequency calculations (despite not being stationary points), were then done using ORCA at the standard level of theory. The electronic energy of the quasi-MECP is taken as the average of the singlet and triplet energies

at the point where the two cross; because frequencies are calculated for nonstationary species and because the changes in entropy are likely to be small, the Gibbs free energy correction for the quasi-MECP is taken as the change in enthalpy between the quasi-MECP and the preceding point (*i.e.*, **II**). The singlet structures are used in the analysis herein. The resulting surfaces are shown in Figure S26-S28.

Analysis of the electronic structures of selected systems was done using natural bond orbitals (NBO)[28] using Weinhold and coworkers' NBO7 (version 7.0.10) program[29] called from within ORCA. In particular, the Wiberg bond orders,[30] second-order perturbative estimates of the NBO interactions (E(2)),[28] and the natural resonance theory (NRT) analysis were considered.[31-36]

By default, thermochemical corrections to the electronic energy are calculated at 298.15 K and a pressure of 1 atm; this state will be denoted as ΔG^{o}_{298}. However, this generally does not correspond to the reaction conditions (which will be denoted as $\Delta G'_{298}$). The relation between the two is:

$$\Delta G'_{298} = \Delta G^{0}_{298} + R \cdot T \cdot \ln\left(\frac{Q'}{Q^{o}}\right)$$

Where R is the universal gas constant (0.001987 kcal/mol·K = 0.082057 L·atm/mol·K) and $Q = \frac{\prod_i [product\ i]}{\prod_i [reactant\ i]}$ (where $[a]$ is the concentration of species a) is the reaction quotient. If we assume an ideal gas, then $pV = nRT$ or $[a] = \frac{n}{V} = \frac{p}{RT}$ or, at 298.15 K and 1 atm, $[a] = \frac{1}{24.5}$ M.[37] If we consider the reaction BA + DMSO → BA·DMSO (a typical A + B → C reaction), at full conversion [BA·DMSO] = [BA] and $Q^0 = 24.5\ \mathrm{M}^{-1}$ and $Q' = \frac{1}{[\mathrm{DMSO}]}$. If we assume that in a typical experiment, [BA] = 0.3 M (BA = boranoanthracene) and 1-100 equivalents of DMSO are used, then one obtains the corrections to the free energy in Table S1. There is much discussion in the literature as to the appropriateness for complexation free energies of the standard assumptions typically used (i.e., the ideal gas–rigid rotor–harmonic oscillator or IGRRHO), but Besora *et al.* demonstrated that the use of a dispersion-correction DFT functional (such as the PBE0-D3BJ used in this study) is actually the recommended approach.[38]

Table S1. Corrections to the free energy of formation of the boranoanthracene·DMSO complex (I) as a function of DMSO concentration.

[DMSO] (M)	$\cdot T \cdot \ln\left(\frac{Q'}{Q^o}\right)$
0.3	-1.18
1.0	-1.89
3.0	-2.55
6.0	-2.96
10.0	-3.26
30.0	-3.91

This same concentration correction can be applied to the rest of the reaction profile. For each step where there is an unequal number of reactants and products (in our case, either an A+B→C or A→B+C reaction), such a correction can be applied; note that the corrections in Table S1 are for the former reaction, and the correction for the latter would be the negative of these corrections. For steps downstream of the formation of the DMSO adduct **I**, all species concentrations are the same as [BA]. Thus, for each step, the concentration correction would be the sum of the corrections from previous steps plus the correction for the specific step. All free energies (ΔG_{298}) are for [BA] = 0.3 M and [DMSO] = 6.0 M.

Complete active space self-consistent field (CASSCF)[39-45] calculations and the corresponding complete active space second-order Møller–Plesset (CASPT2)[46] calculations were run using ORCA 6.0.1. In these calculations, the def2-SVPD basis set was used. The active space was determined using Grimme and Hansen's fractional occupation number (FOD) method[47] following the recommendation by Bauer *et al.* to use occupied and virtual orbitals with FOD occupations significantly different from 0 or 2 (Table S2).[48] Four roots were considered: the lowest triplet state and the three lowest singlet states.

Discussion:

The reaction of the boranoanthracene with DMSO was shown to follow a two-step reactivity. The open-shell singlet (biradical) was not considered because DFT is a single-reference method that cannot properly describe such a system. In order to understand the importance of the open-shell singlet, CASSCF/CASPT2 calculations were performed. The active space

for these calculations was chosen based on Grimme and Hansen's fractional occupation number (FOD) method,[47] specifically the recommendation by Bauer *et al.* to use occupied and virtual orbitals with FOD occupations significantly different from 0 or 2.[48] Based on the FOD occupancies of the intermediate **II** for TMS-BA and ^{i}Pr-BA (Table S) an active space of 4 electrons in 3 orbitals (*i.e.*, a CAS(4,3) active space) was chosen. In both cases, the lowest open-shell singlet and the triplet are very close in energy, while the closed-shell singlet is dramatically higher in energy. Thus, it is reasonable to consider where the closed-shell singlet and triplet states cross (*i.e.*, either the MECP or qMECP) to get insight into the reaction mechanism, and the small difference in energy between the triplet and open-shell singlet states is not going to have any meaningful impact on the conclusions of this study.

Table S2. FOD occupations of the MOs around the HOMO–LUMO gap for II for the TMS_2,$^{i}Pr_2$ and Ph_2 systems and III for the Ph_2 system.

**Molecular Orbital**	**TMS**	**^{i}Pr**	**Ph(II)**	**Ph(III)**
HOMO-3	1.9942	1.9953	1.9935	1.9880
HOMO-2	1.9846	1.9857	1.9862	1.9826
HOMO-1	1.9694	1.9221	1.9027	1.8763
HOMO	1.2448	1.3309	1.3194	1.5119
LUMO	0.8031	0.7587	0.7803	0.6588
LUMO+1	0.0069	0.0064	0.0082	0.0047

Table S2. CASSCF and CASPT2 results, using the def2-SVPD basis set, for **II** for the TMS_2, $^{i}Pr_2$ and Ph_2 systems and **III** of the Ph_2 system, including the weight of the primary configuration and the relative energies (ΔE, kcal/mol) of the triplet and two singlet states.

**Root**	**Weight of Primary Configuration**				**ΔE_{CASSCF}**				**ΔE_{CASPT2}**			
	**TMS**	**^{i}Pr**	**Ph(II)**	**Ph(III)**	**TMS**	**^{i}Pr**	**Ph(II)**	**Ph(III)**	**TMS**	**^{i}Pr**	**Ph(II)**	**Ph(III)**
trip	1.000	1.000	0.987	0.986	0.11	0.88	1.22	0.00	0.53		1.73	0.91
sing(OS1)	0.993	0.982	0.949	0.951	0.00	0.00	0.00	0.04	0.00		0.00	0.00
sing(OS2)	0.830	1.000	—[a]	—[a]	83.93	46.75	—[a]	—[a]	65.27		—[a]	—[a]
sing(CS)	0.824	0.982	0.868	0.863	69.08	100.08	50.89	9.37	48.08		49.22	8.11

[a] Only 2 singlet roots were considered for the Ph_2 system.

DMSO coordination discussion:

In the reaction of the Ph-BA **1d** with DMSO, the free energy (ΔG^{o}_{298}) of formation of the initial complex **I** is positive, 5.3 kcal/mol (Table S5), meaning the formation of the DMSO-adduct is energetically unfavorable. When considering the reaction pathway, this is not a problem; **I** is lower in energy than the subsequent qMECP. *Prima facie*, it would appear, however, that the formation of **I** (**1d**-DMSO adduct) is unfavorable and that it should not be observed in the NMR studies reported here. There are two factors that need to be considered: corrections for concentration and equilibrium.

The free energy of formation of the DMSO-adduct of Ph-BA **1d** (*i.e.*, **I**) is 5.3 kcal/mol. As discussed in the "Computational Methods Section" of the SI, the default conditions used in computing the corrections for free energy is 1 atm of pressure at 298.15 K. The corresponding free energy can be corrected to any concentration as described in the "Computational Methods Section". If one applies the corrections in Table S1, the free energy of formation falls to 2.4 kcal/mol if one has 20 equivalents of DMSO (which is the conditions used in the NMR experiments) and even further if higher DMSO concentrations are used. This makes it more reasonable given both the inherent error associated with DFT methods and the fact that the complexation reaction is an equilibrium and not a full conversion of the boranoanthracene to the DMSO complex. If one considers the ^{1}H-NMR spectra (Figure 7), one notes that the primary signal is from the Ph-BA (**1d**) and not the DMSO complex. Therefore, one would expect a small but positive free energy of formation relative to free Ph-BA (**1d**) and DMSO. Integration of the ^{1}H NMR signals of the complexed DMSO methyl groups and the C9/C10 hydrogen atoms (Figure 7) gives an approximate free to complexed boranoanthracene ratio of ~10:1. In such circumstances, a free energy of complexation of 2.4 kcal/mol is reasonable.

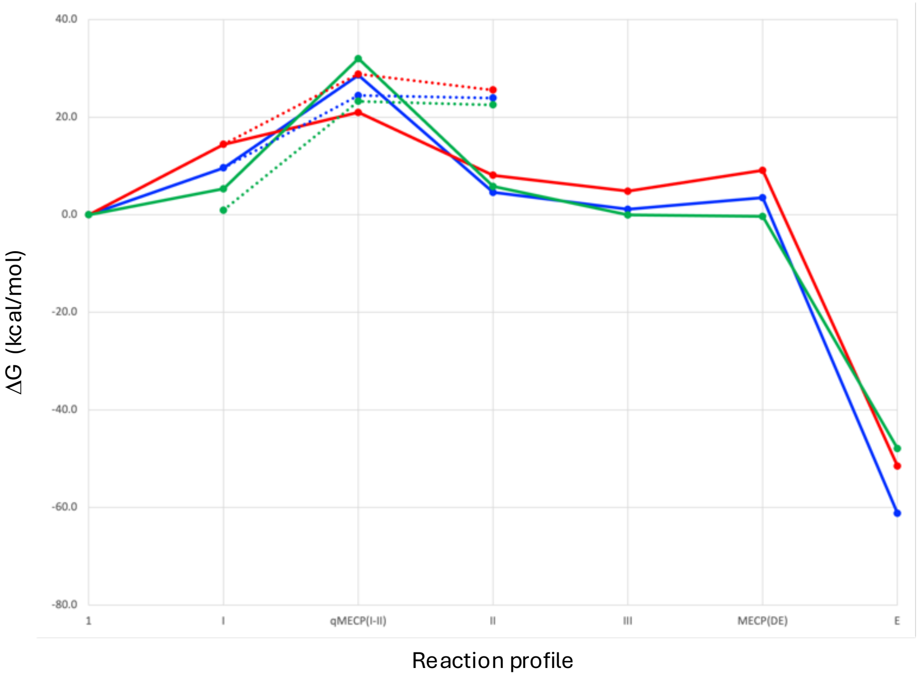

Figure S20: Reaction profile (ΔG_{298} of singlet – solid lines, and triplet – dashed lines) for the decomposition of the boranoanthracene adducts for R = TMS (blue), Ph (green) and ^{i}Pr (red) via singlet-triplet two-state reactivity.

Table S4. Selected bond distances (Å).

	^{i}Pr				TMS				Ph			
	B–C	B···C	B···O	O···S	B–C	B···C	B···O	O···S	B–C	B···C	B···O	O···S
1 (s)	1.624	1.625	—	—	1.636	1.636	—	—	1.612	1.612	—	—
1 (t)									1.689	1.696	—	—
I (s)	1.638	1.640	2.472	1.497	1.689	1.696	1.618	1.546	1.669	1.692	1.573	1.551
qMECP$_{I\text{-}II}$	1.873	1.973	1.602	1.525	1.626	2.958	1.376	1.639	1.641	2.565	1.411	1.591
TS$_{I\text{-}II}$ (s)	1.633	2.429	1.478	1.570	1.622	2.631	1.458	1.591	1.607	2.666	1.437	1.589
II (t)	1.624	4.203	1.349	2.299	1.636	3.921	1.330	2.314	1.620	4.122	1.331	2.271
II (s)	1.614	3.163	1.434	1.598	1.612	2.981	1.437	1.606	1.599	2.938	1.423	1.599
III (t)	1.648	3.955	1.335	—	1.606	3.745	1.311	—	1.635	3.783	1.265	—
MECP$_{III\text{-}IV}$ (t→s)	1.599	4.319	1.367	—	1.608	3.745	1.311	—	1.617	3.704	1.264	—
IV (s)	—	—	1.221	—	—	—	1.221	—	—	—	1.214	—

Table S5. Reaction enthalpies (ΔH_{298}, kcal/mol) and Gibbs' free energies (at standard state ΔG^{0}_{298} and corrected for concentration ΔG_{298}, kcal/mol).

	ΔH_{298}			ΔG^{0}_{298}			ΔG_{298}		
	TMS	^{i}Pr	Ph	TMS	^{i}Pr	Ph	TMS	^{i}Pr	Ph
1 (s)	0.0	0.0	0.0	0.0	0.0	0.0	0.0	0.0	0.0
1(t)			76.9			75.1			75.1
I (s)	-4.2	0.9	-9.2	9.6	14.4	5.3	6.7	11.5	2.4
qMECP$_{I\text{-}II}$	14.9[a]	7.5a	17.5	28.6a	21.0a	32.0	25.7a	18.0a	29.0[a]
TS$_{I\text{-}II}$ (s)	10.6	14.4	8.3	24.5	28.8	23.3	21.5	25.9	20.3
II (t)	-5.0	-2.4	-6.9	4.6	8.1	5.8	1.6	5.1	2.9
II (s)	10.2	11.7	8.7	23.9	25.6	22.5	21.0	22.6	19.6
III (t)	4.0	6.9	-0.4	1.1	4.8	0.0	-0.6	3.1	-1.8
MECP$_{III\text{-}IV}$ (t→s)	3.4	8.4	-1.0	3.5	9.1	-0.3	1.7	7.3	-2.1
IV (s)	-45.1	-37.2	-33.6	-61.2	-51.5	-47.9	-61.8	-52.1	-48.5

[a] Quasi-MECP point; see the Computational Methods section of the SI for details.

Table S6. Optimized geometries for iPr-BA **1a** and TMS-BA **1c** intermediates and transition states. (Color scheme: H – white; C – gray; O – red; N – blue; B – pink; S – yellow; Si – teal.)

	**^{i}Pr**	**TMS**	**Ph**
1(s)	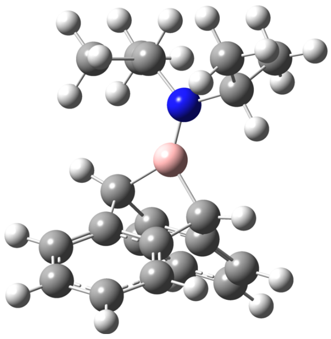	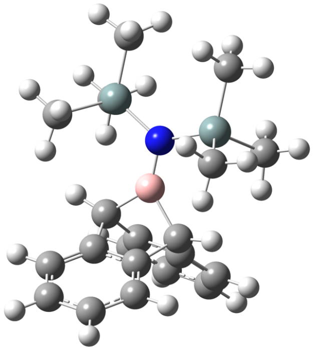	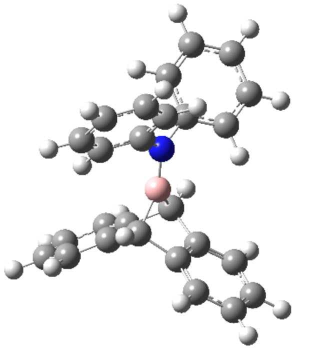
1(t)			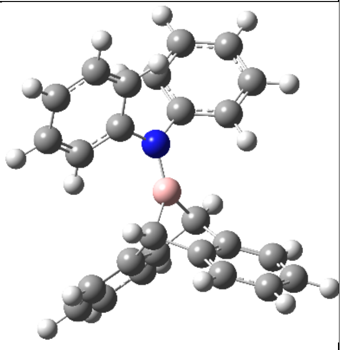
I (s)	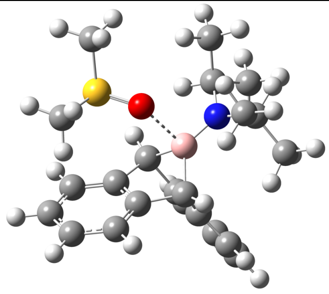	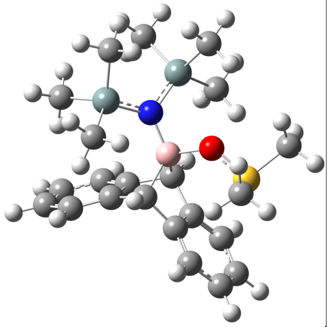	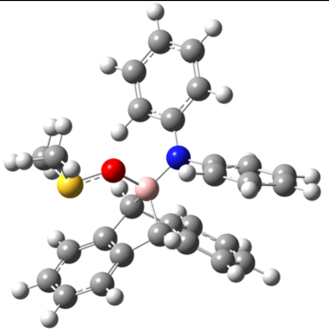
qMECP$_{I-II}$ (s→t)	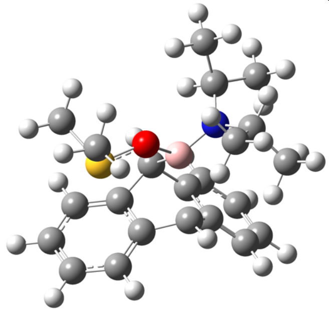	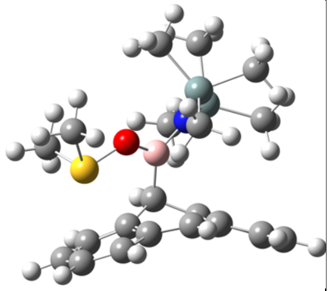	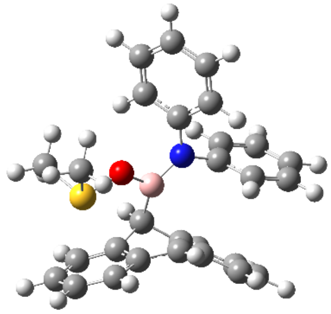

TS_{I-II} (s)	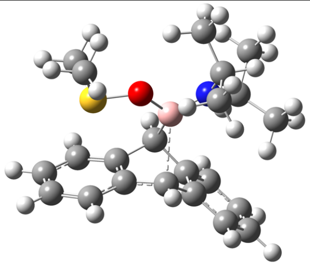	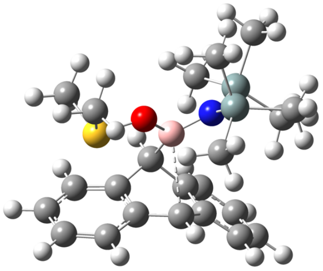	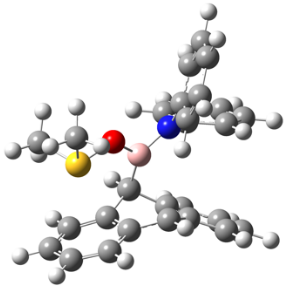
II (t)	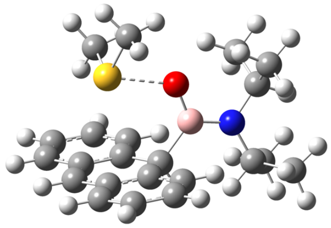	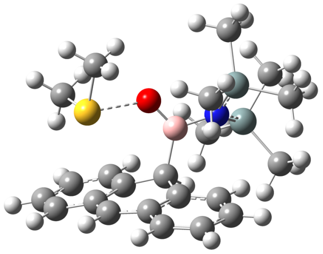	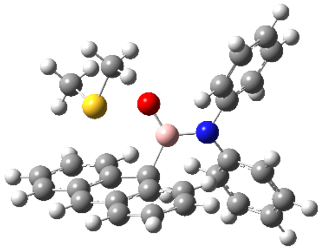
II (s)	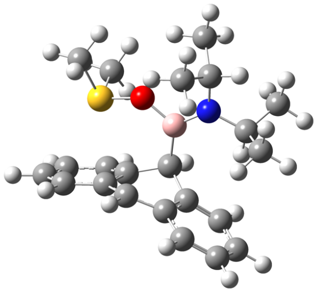	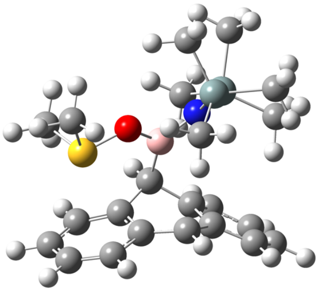	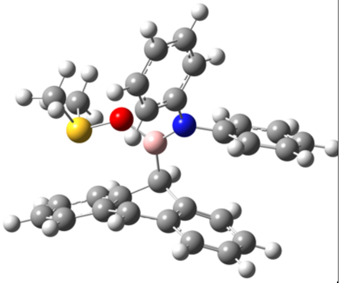
III (t)	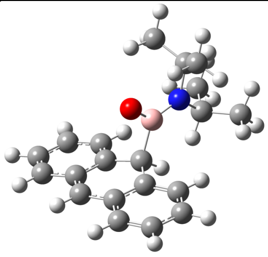	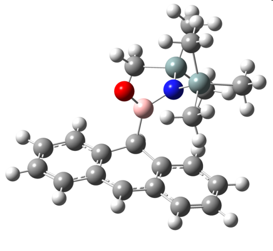	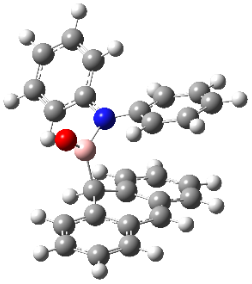
$MECP_{III-IV}$ (t→s)	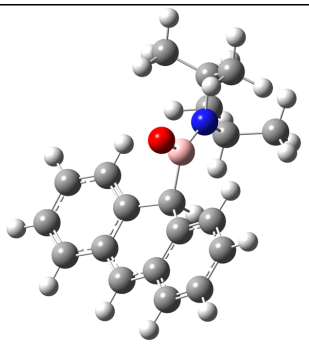	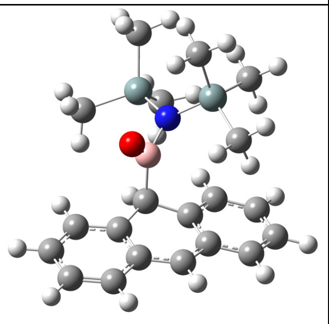	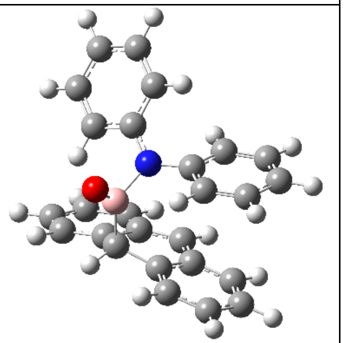

IV (s)	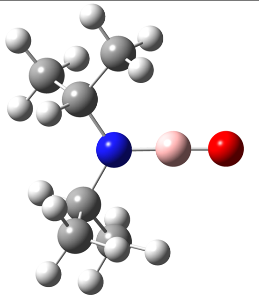	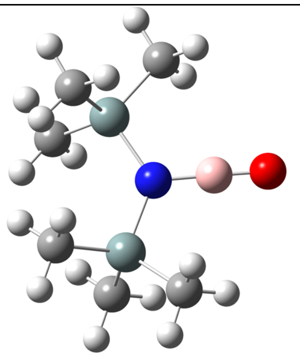	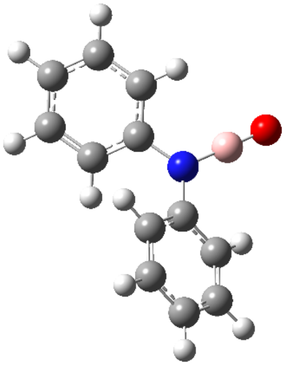

Table S7. Bond indices in ^{i}Pr-oxoborane.

**Bond**	**Wiberg**	**NBI**	**NRT**		
			**Total**	**Covalent**	**ionic**
B–N	1.0055	1.0027	1.4077	0.5305	0.8772
B–O	1.7435	1.3204	2.5812	0.9647	1.6165
N–C	0.9397	0.9694	0.9762	0.7260	0.2502
	0.9424	0.9708	0.9847	0.7277	0.2569
∑B	2.8384				
∑N	3.0072				
∑O	1.8428				

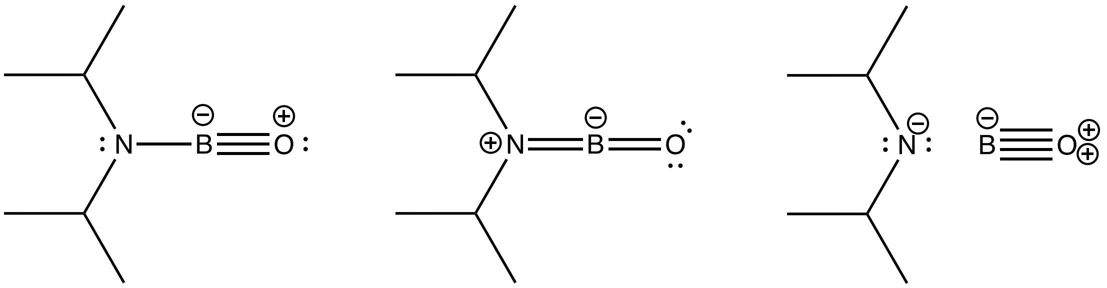

26.87% 24.96% 4.04%

Figure S21. Lead resonance structures in the NRT analysis of ^{i}Pr-aminoxoborane.

Table S8. Primary donor-acceptor stabilizations from an E(2) analysis iPr-BA (**1a**).

Donor	Acceptor	E(2) (kcal/mol)
LP(1) N	BD*(2) B–O	29.97
	BD*(2) C–C	8.88
LP(1) O	BD*(1) B–N	6.25
	Ry(1) B	12.58

Table S9. Bond indices in TMS-oxoborane.

Bond	Wiberg	NBI	NRT		
			**Total**	**Covalent**	**ionic**
B–N	1.0258	1.0128	1.3769	0.5043	0.8726
B–O	1.7408	1.3194	2.5815	0.9564	1.6252
N–Si	0.5846<br>0.5831	0.7646<br>0.7636	1.0416<br>1.0141	0.5043<br>0.3080	0.8726<br>0.7061
∑B	2.8295				
∑N	2.3669				
∑O	1.8370				
∑Si	3.1362<br>3.1384				

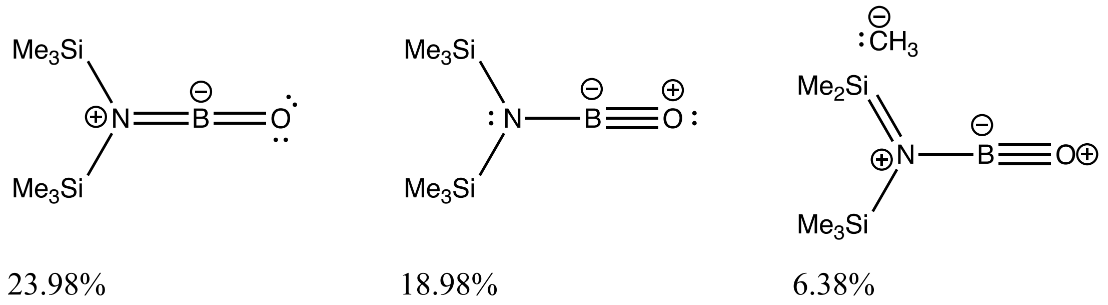

Figure S22. Lead resonance structures in the NRT analysis of TMS-aminoxoborane.

Table S10. Primary donor-acceptor stabilizations from an E(2) analysis for TMS-BA **1c**.

**Donor**	**Acceptor**	**E(2) (kcal/mol)**
LP(1) N	BD*(1) B–O	49.69
LP(1) O	Ry(1) B	13.01

Table S11. Bond indices in Ph_2-oxoborane.

Bond	Wiberg	NBI	NRT		
			**Total**	**Covalent**	**ionic**
B–N	0.9317	0.9653	1.2524	0.4846	0.7678
B–O	1.7981	1.3409	2.6448	1.0019	1.6429
N–C	0.9973<br>0.9871	0.9987<br>0.9935	0.9868<br>0.9644	0.7179<br>0.7132	0.2689<br>0.2511
∑B	2.8280				
∑N	3.1645				
∑O	1.8895				

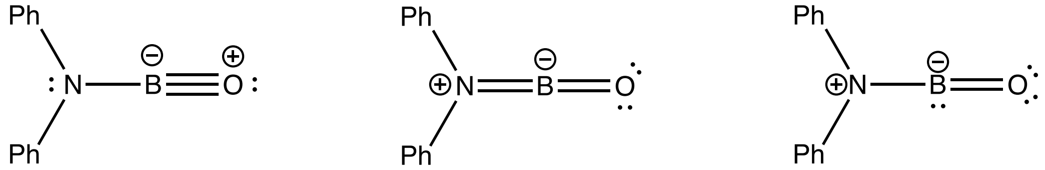

18.86%[a] 17.10%[a] 3.53%[b]

Figure S23. Lead resonance structures in the NRT analysis of Ph-aminoxoborane.

Table S12. Primary donor-acceptor stabilizations from an E(2) analysis for Ph-BA **1d** excluding those within the phenyl rings.

**Donor**	**Acceptor**	**E(2) (kcal/mol)**
LP(1) N	LV(1) B	70.21
LP(1) N	BD*(2) C_i–C_m (Ph1)	22.80
LP(1) N	BD*(2) C_i–C_m (Ph2)	17.07
LP(2) O	LV(1) B	136.07

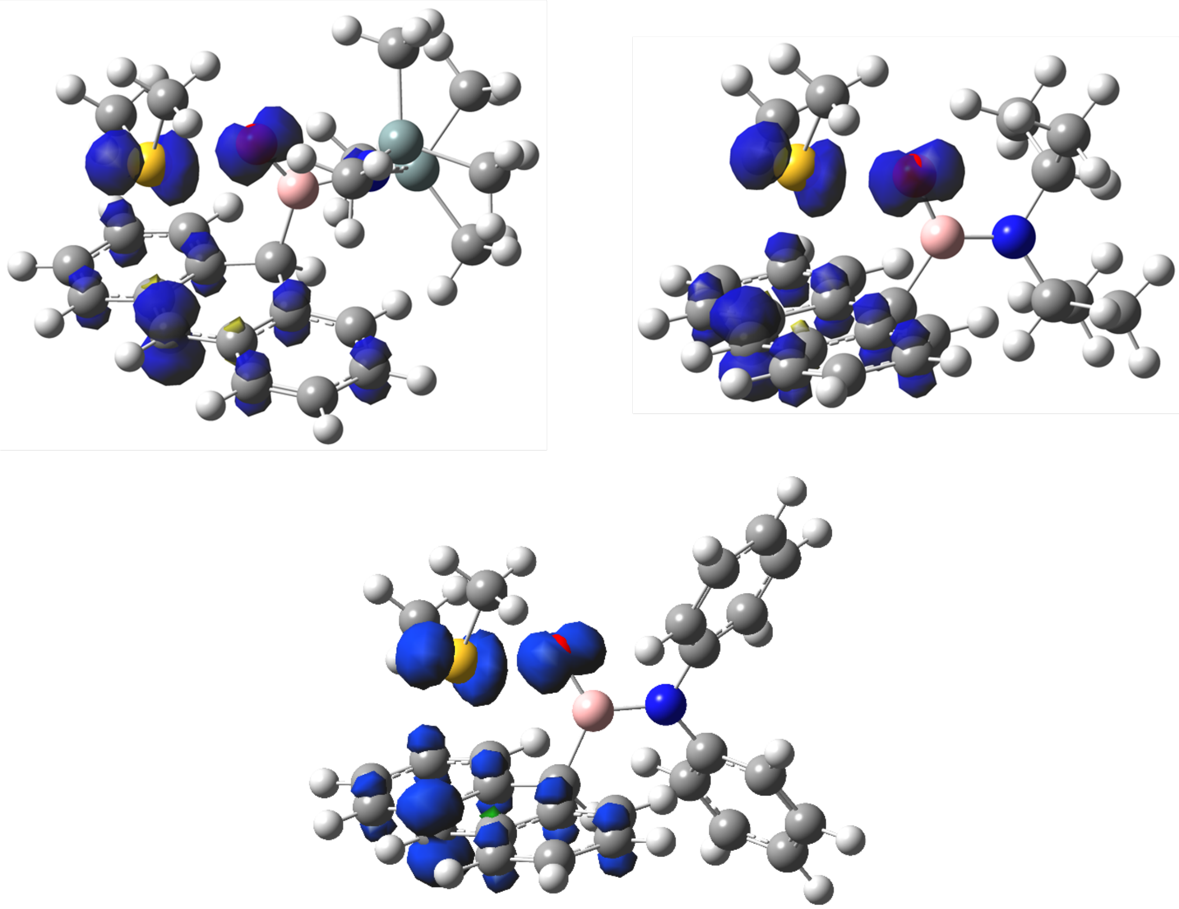

Figure S24. Electronic spin density ($\boldsymbol{\rho_\sigma}$) plots (0.01 [units e^{-}/Å^{2}] isosurface) of the triplet intermediate before loss of Me_2S for (intermediate **II**) R = TMS (top left), R = ^{i}Pr (top right) and R = Ph (bottom).

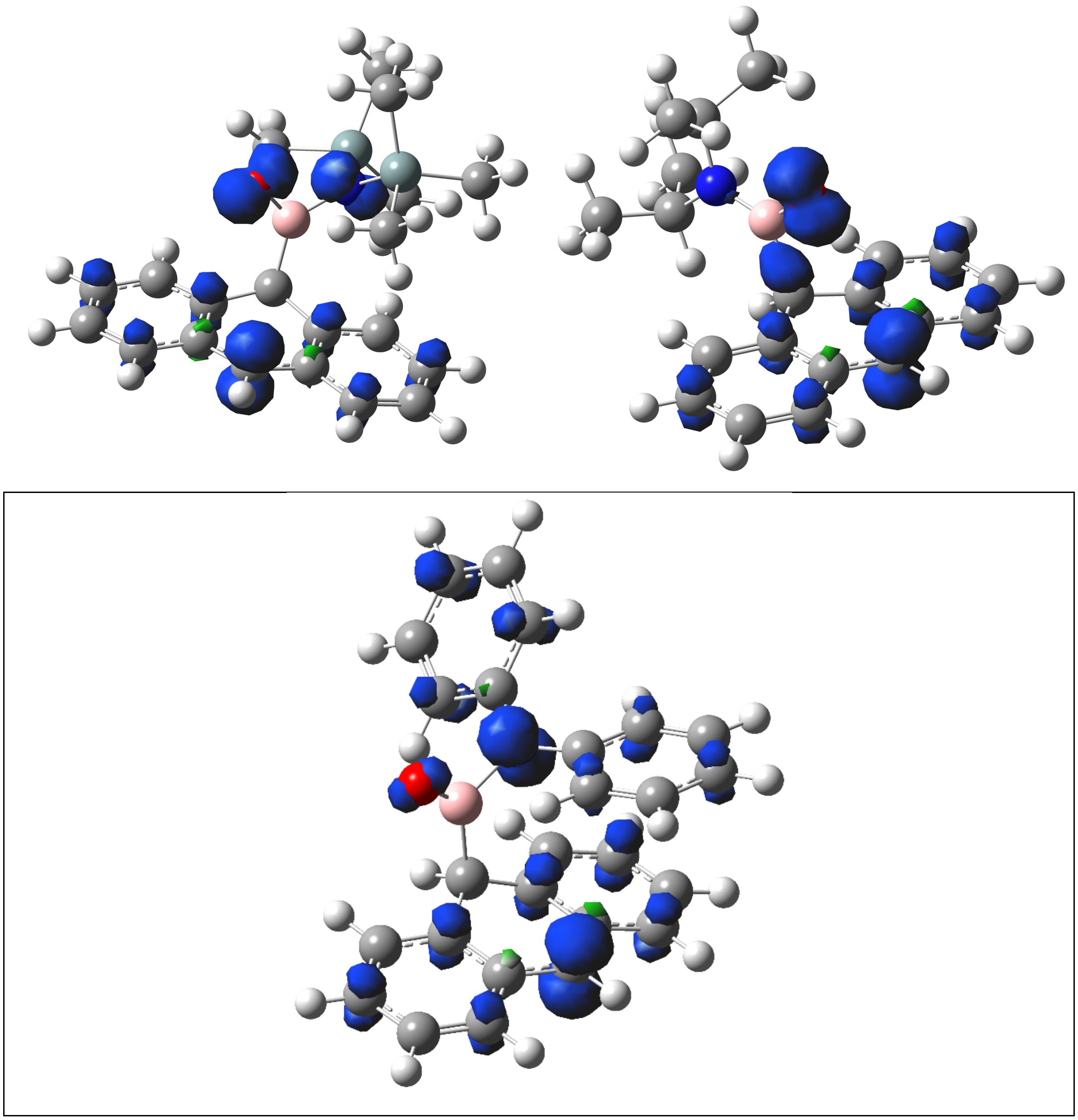

Figure S25. Electronic spin density ($\boldsymbol{\rho_\sigma}$) plots (0.01 [units e^{-}/Å^{2}] isosurface) of the triplet intermediate after loss of Me_2S (intermediate **III**) for R = TMS (top left), R = ^{i}Pr (top right) and R = Ph (bottom).

Table S13. Natural Population Analysis (NPA) Partial Atomic Spin Densities and Partial Atomic Charges from the Natural Resonance Theory (NRT) Analysis on the Borylene–Anthracene Intermediates (**III**).

**Atom Centre**	**Partial Spin Density**		**Partial Charges**	
	**R = ^{i}Pr**	**R = TMS**	**R = ^{i}Pr**	**R = TMS**
B	-0.0324	-0.0381	-1.0210	-0.8592
N	0.0288	0.3342	0.6937	0.5510
O	0.8085	0.6770	0.4306	0.4007
C10	0.5684	0.5561	-0.0257	-0.0446
C9	0.1428	0.0160	0.0875	0.0308
C(H)	0.0043<br>0.0010	—	-0.0151<br>-0.0073	—
Si		-0.0102<br>-0.0101		0.0085<br>-0.0131

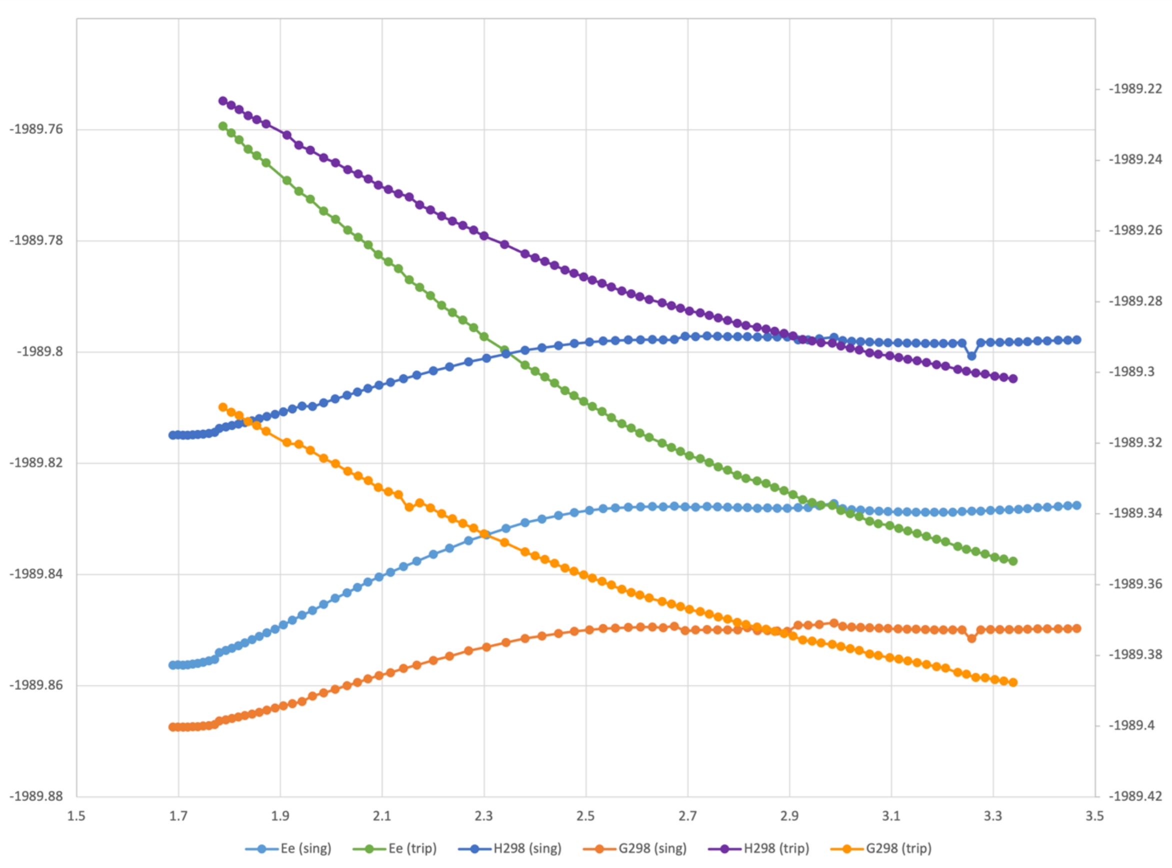

Figure S26. Relaxed scans of the B–C bond length in **1c**. The electronic energy (E_e, in Hartrees) is on the left axis, while the enthalpy (H_{298}, in Hartrees) and Gibbs free energy (G_{298}, in Hartree) are on the right axis.

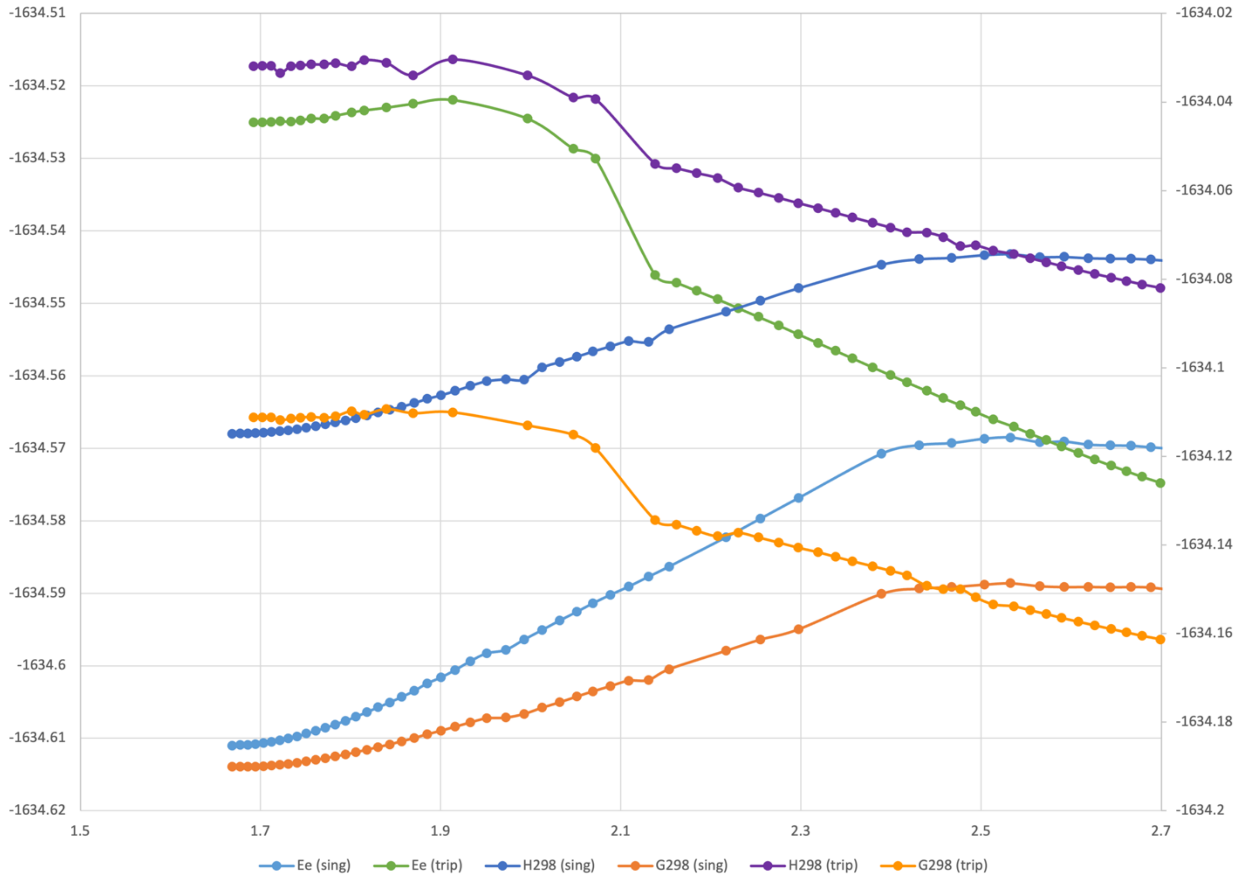

Figure S27. Relaxed scans of the B–C bond length in **1d**. The electronic energy (E_e, in Hartrees) is on the left axis, while the enthalpy (H_{298}, in Hartrees) and Gibbs free energy (G_{298}, in Hartree) are on the right axis.

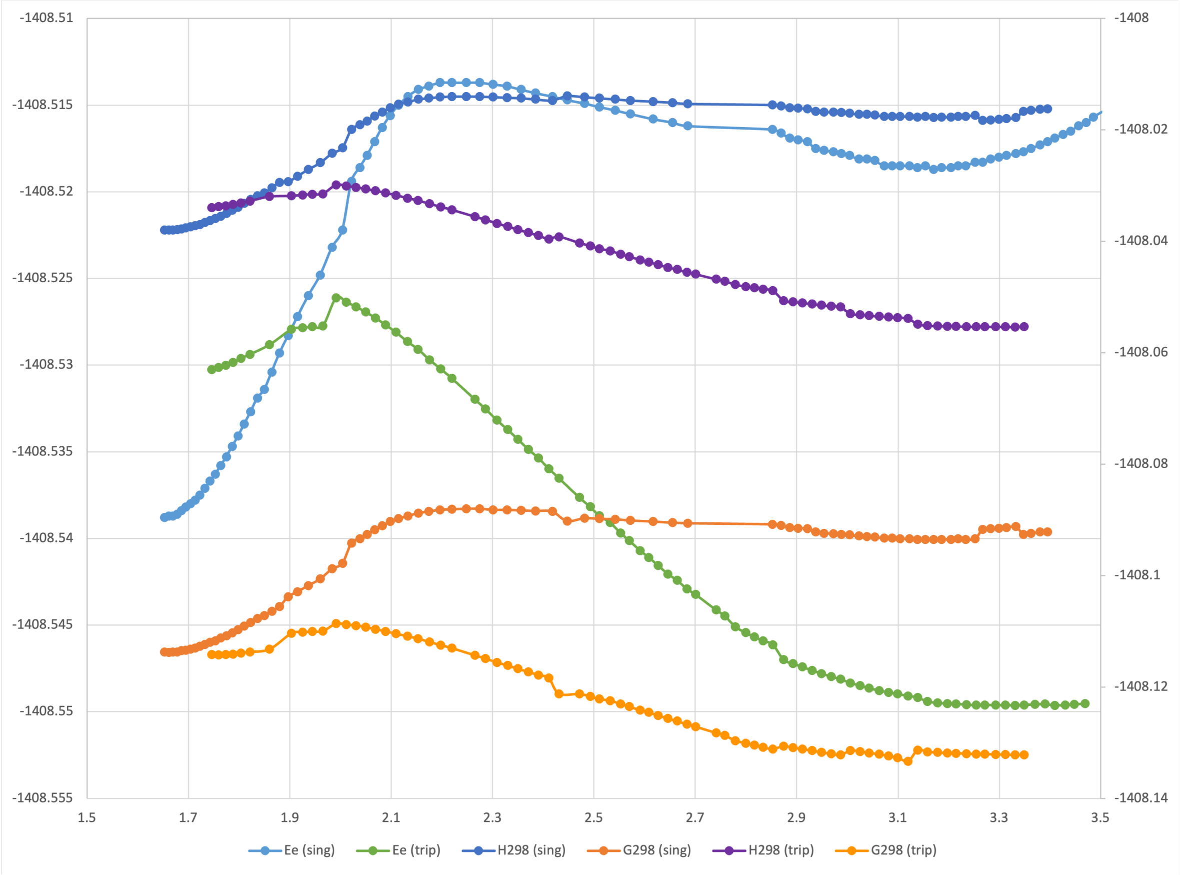

Figure S28. Relaxed scans of the B–C bond length in **1a**. The electronic energy (E_e, in Hartrees) is on the left axis, while the enthalpy (H_{298}, in Hartrees) and Gibbs free energy (G_{298}, in Hartree) are on the right axis.

Table S14. Bond indices in MeBO.

Bond	Wiberg	NBI	NRT		
			Total	Covalent	Ionic
B–C	0.9927	0.9963	1.0419	0.6531	0.3888
B–O	1.8364	1.3551	2.9581	1.1041	1.8540
∑B	2.8631				
∑O	1.8831				

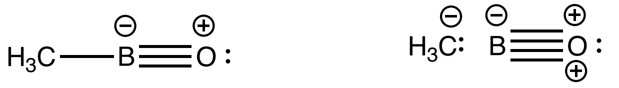

86.91%   4.45%

Figure S29. Lead resonance structures in the NRT analysis of MeBO.

Table S15. Primary donor-acceptor stabilizations from an E(2) analysis for MeBO.

Donor	Acceptor	E(2) (kcal/mol)
LP(1) O	Ry(1) B	12.30

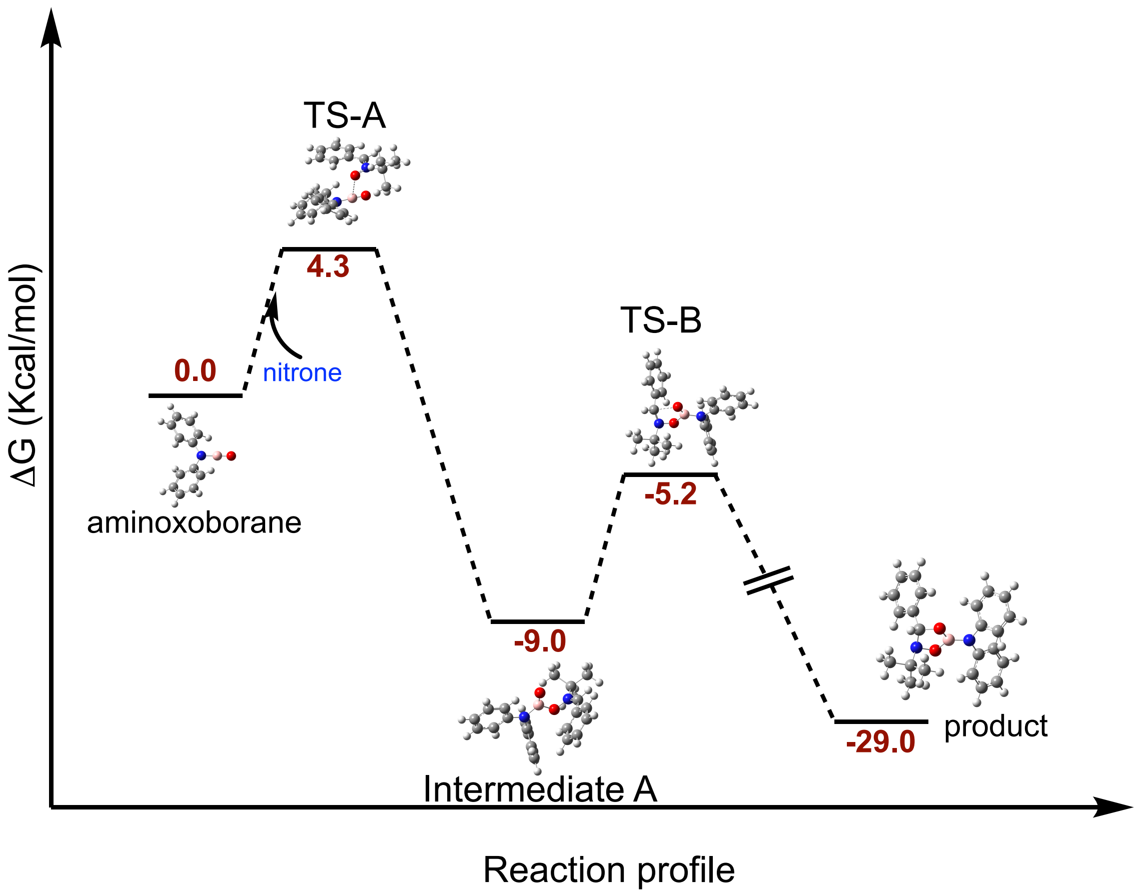

Figure S30: Reaction pathway of the oxoborane with nitrone using DFT computations.

Note added in revision:

Stepwise polar mechanism:

First, we considered the comment of the reviewer regarding the stepwise polar mechanism (zwitterionic intermediate). Using the R = Ph system, we ran further calculations on the reaction mechanism considering S–O bond cleavage in the initial DMSO–boranthracene adduct; the resulting reaction profile is shown below (Figure S31). Loss of dimethylsulfide (DMS) from this initial complex results in a second intermediate that is at ΔG_{298} = 22.5 kcal/mol (all free energies in this discussion are corrected for concentration). As the reviewer noted in his model system, here too there is an N→O dative bond forming an N–B–O three-membered heterocycle. From this intermediate, a transition state for B–C bond cleavage was found, but the resulting barrier is quite significant ($\Delta G^{\ddagger}_{298}$ = 52.4 kcal/mol); moreover, the corresponding product would be the ring-opened system (intermediate III – a zwitterionic closed-shell system). Accordingly, It would seem that closed-shell pathways are not viable.

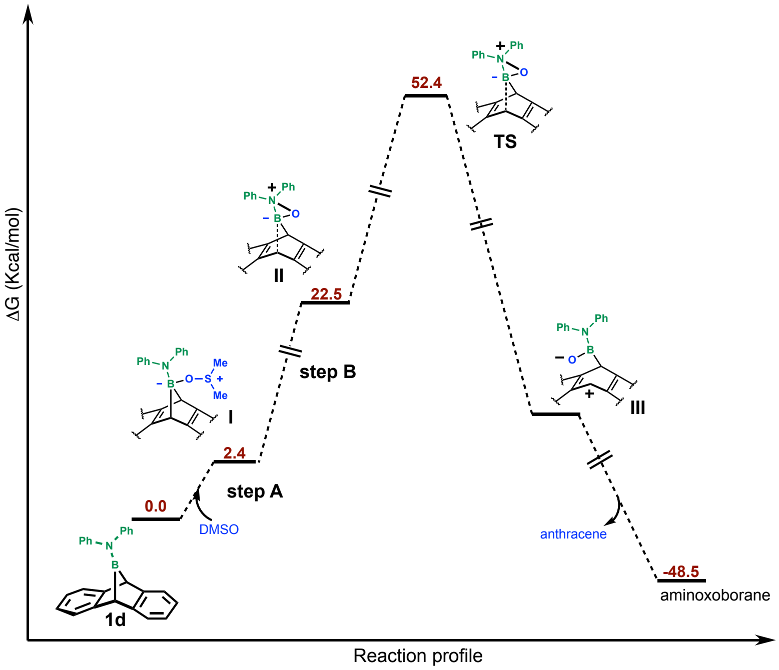

Figure S31: Energy profile for the stepwise polar dissociation of BA **1d**.

Concerted mechanism:

We also considered the R = H system that the reviewer considered, both with the bridged benzene and anthracene systems. In addition to being smaller, the simpler substituent means that it is easier to enforce symmetry (specifically C_S). This allowed us to find transition states for the concerted loss of H_2NBO.

Table S16: The relative free energies for the benzene and anthracene systems.

	**Benzene**	**Anthracene**
Arene-BNH_2 (*compound A*)	0.0	0.0
Arene-BNH_2•••DMSO (*intermediate B*)	19.8	9.4
Arene-$OBNH_2$ + DMS (*intermediate C*)	27.4	26.1
TS for concerted H_2NBO dissociation (N.B. 2nd order saddle point) (*TS D*)	79.2	91.6
Arene + H_2NBO	-80.6	-59.6

First of all, DMSO coordination is quite unfavorable, and the resulting Arene-$OBNH_2$ species (*intermediate C*) are even higher in energy. This would generally indicate that such a pathway is unlikely.

Furthermore, by enforcing C_S symmetry in looking for the concerted extrusion of oxoborane and arene formation (two B–C bonds cleaved), transition states (*TS D*) were found. For both systems, the resulting barriers are quite high in energy (ΔG_{298} = 79.2 and 91.6 kcal/mol for benzene and anthracene, respectively). In addition, the transition states are actually second-order saddle points with a second imaginary frequency corresponding to symmetry-breaking shifting the H_2NBO fragment towards the C_9 position, meaning the reaction follows a two-step mechanism, as originally proposed.

This new data further supports our originally proposed reaction pathway; with a two-step mechanism via a biradical species.

^{1}H-NMR, ^{13}C-NMR, and ^{11}B-NMR spectra

^{1}**H-NMR** (300 MHz, benzene-d_6) of **2a**

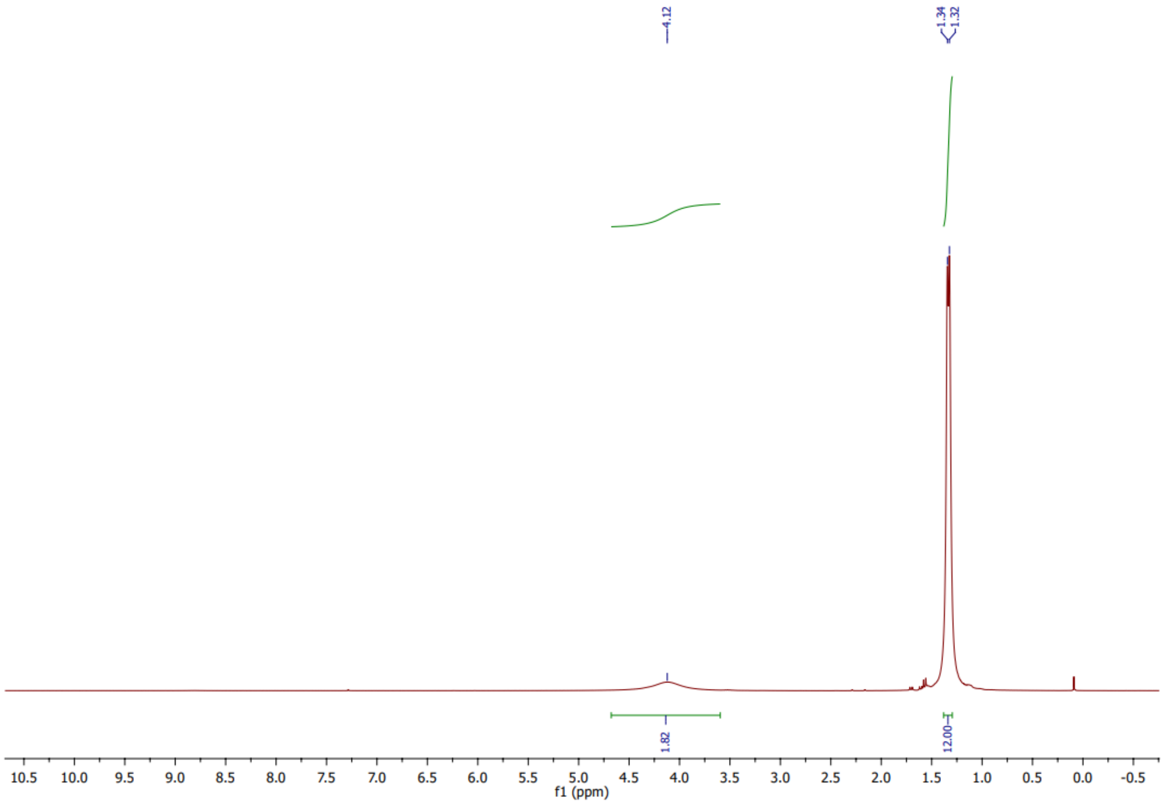

^{11}B-NMR (96 MHz, benzene-d_6) of **2a**

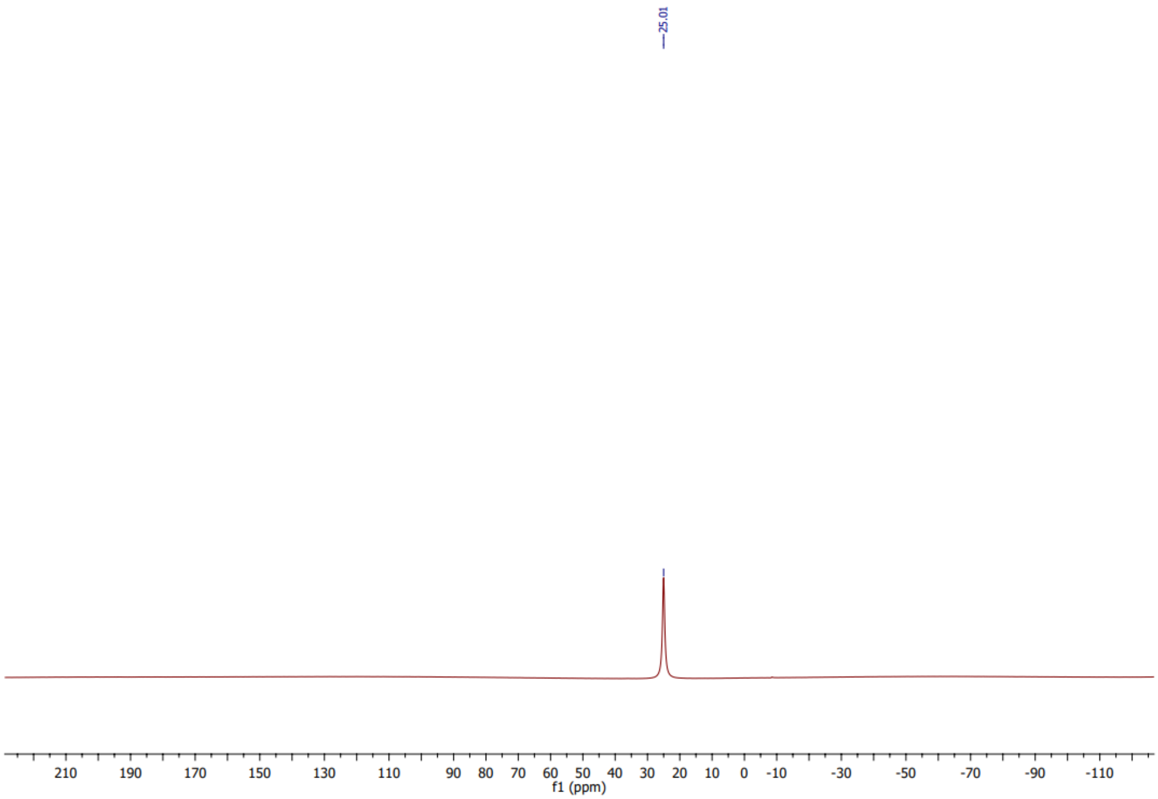

^{1}H-NMR (300 MHz, benzene-d_6) of **2c**

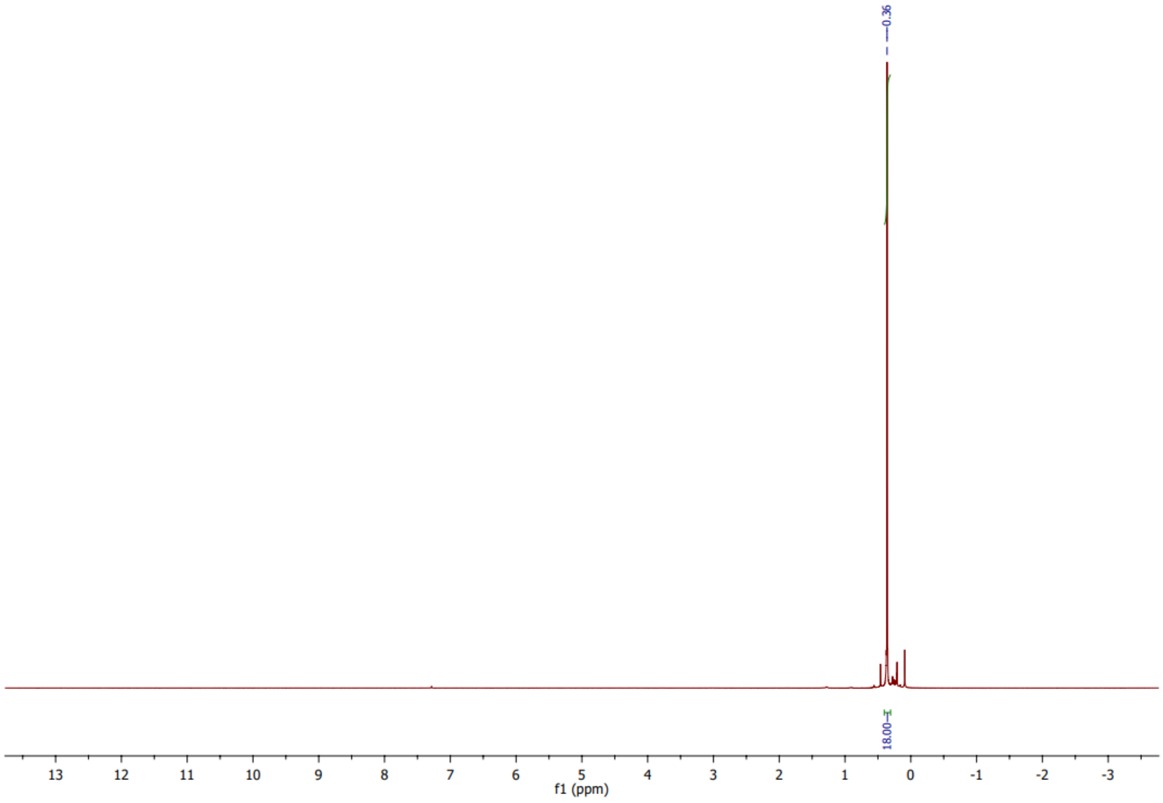

^{11}**B-NMR** (96 MHz, benzene-d_6) of **2c**

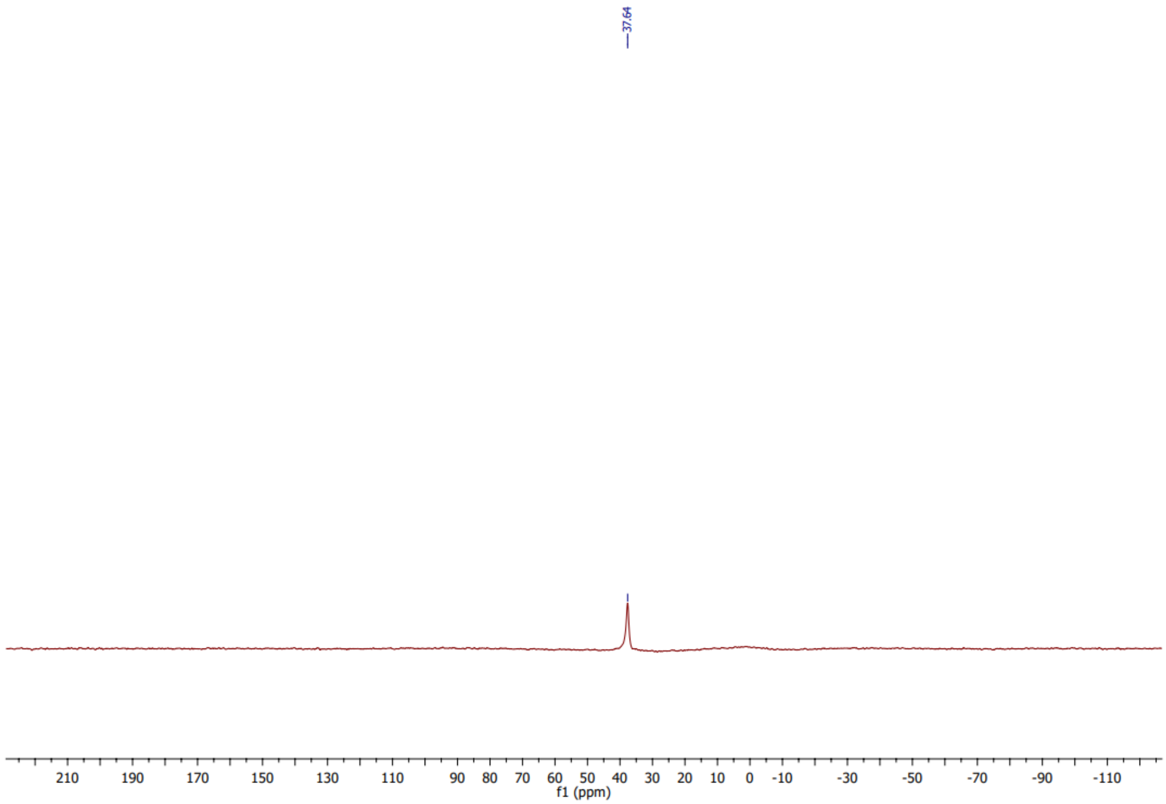

^{1}H-NMR (300 MHz, $CDCl_3$) of **2d**

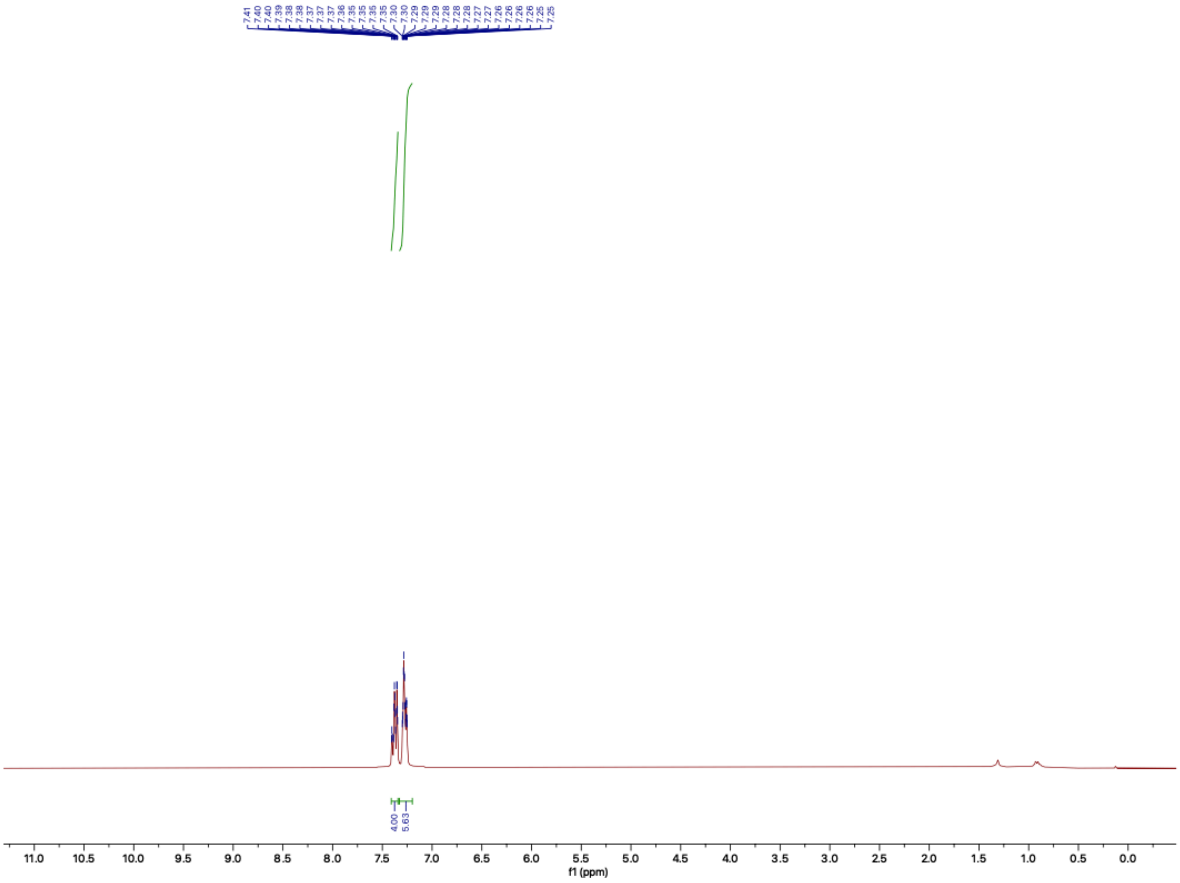

^{11}**B-NMR** (96 MHz, benzene-d_6) of **2d**

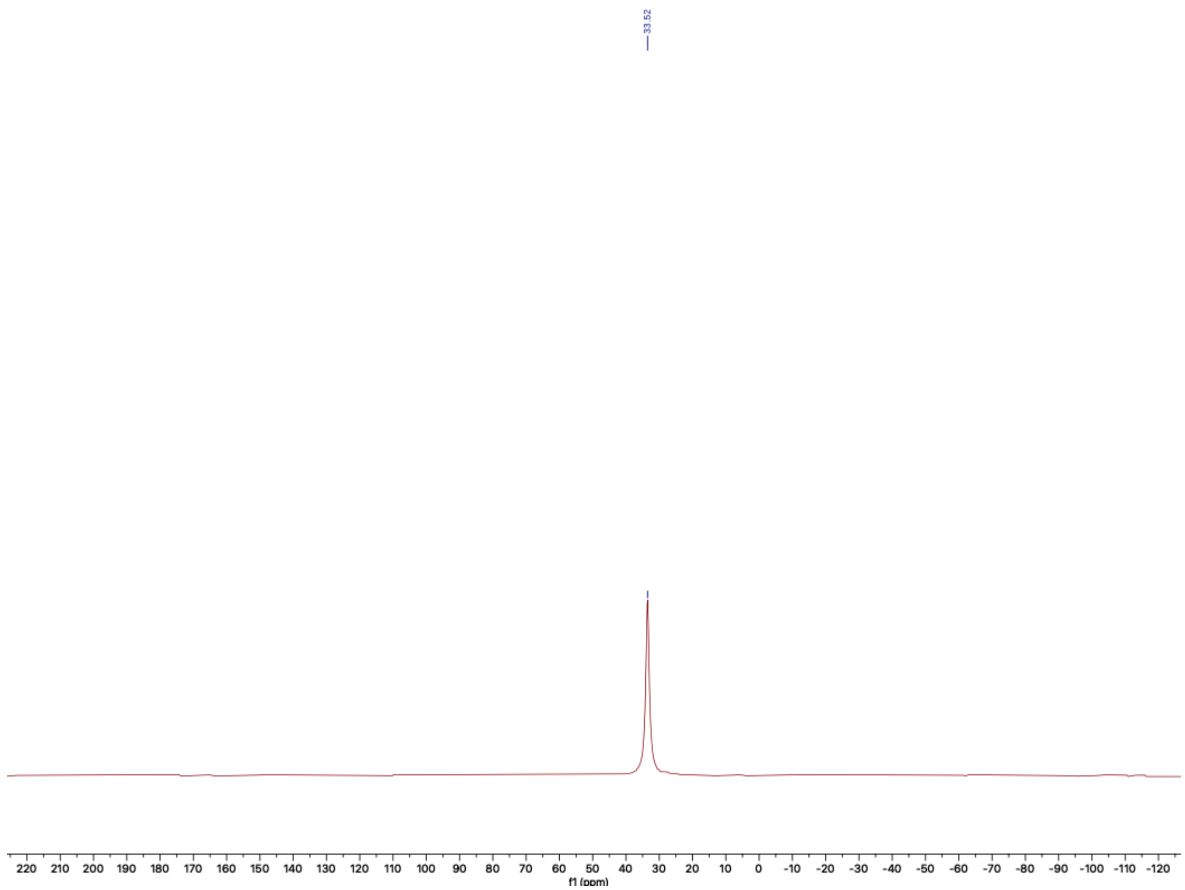

^{1}H-NMR (300 MHz, benzene-d_6) of **2b**

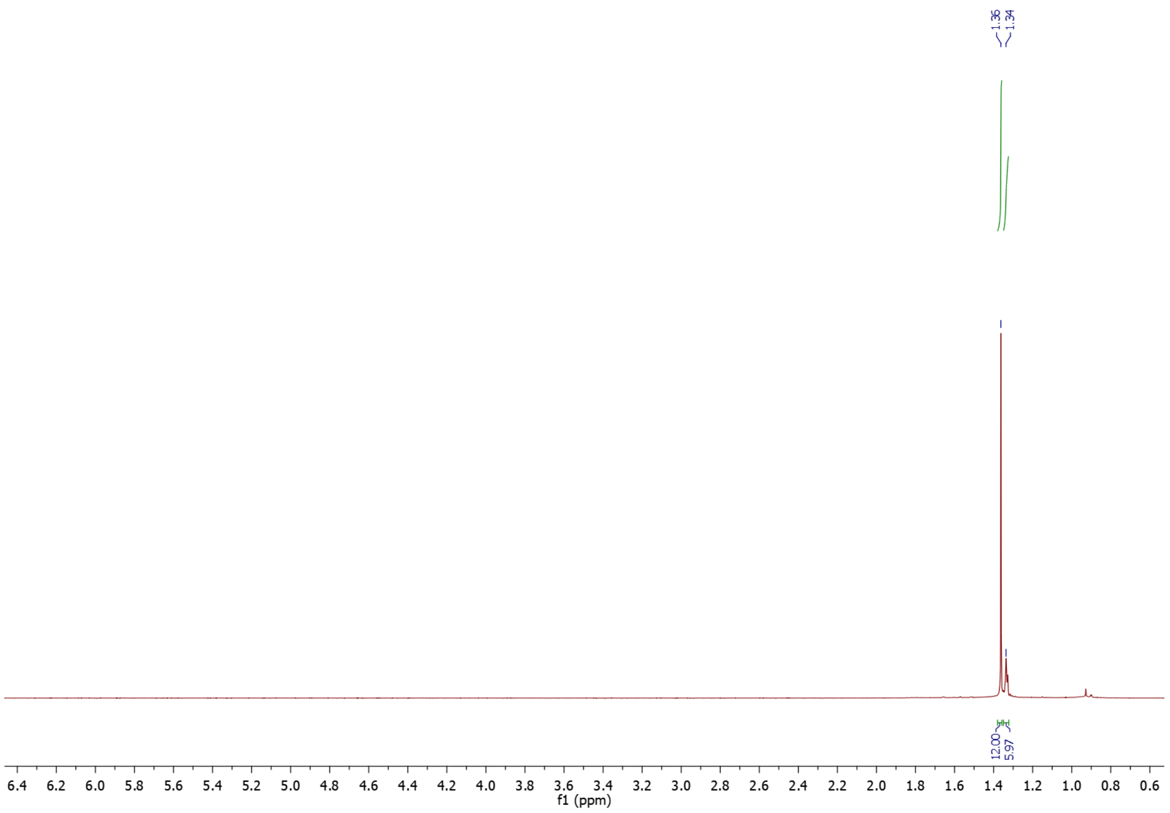

$^{13}C\{^{1}H\}$-NMR (126 MHz, benzene-d_6) of **2b**

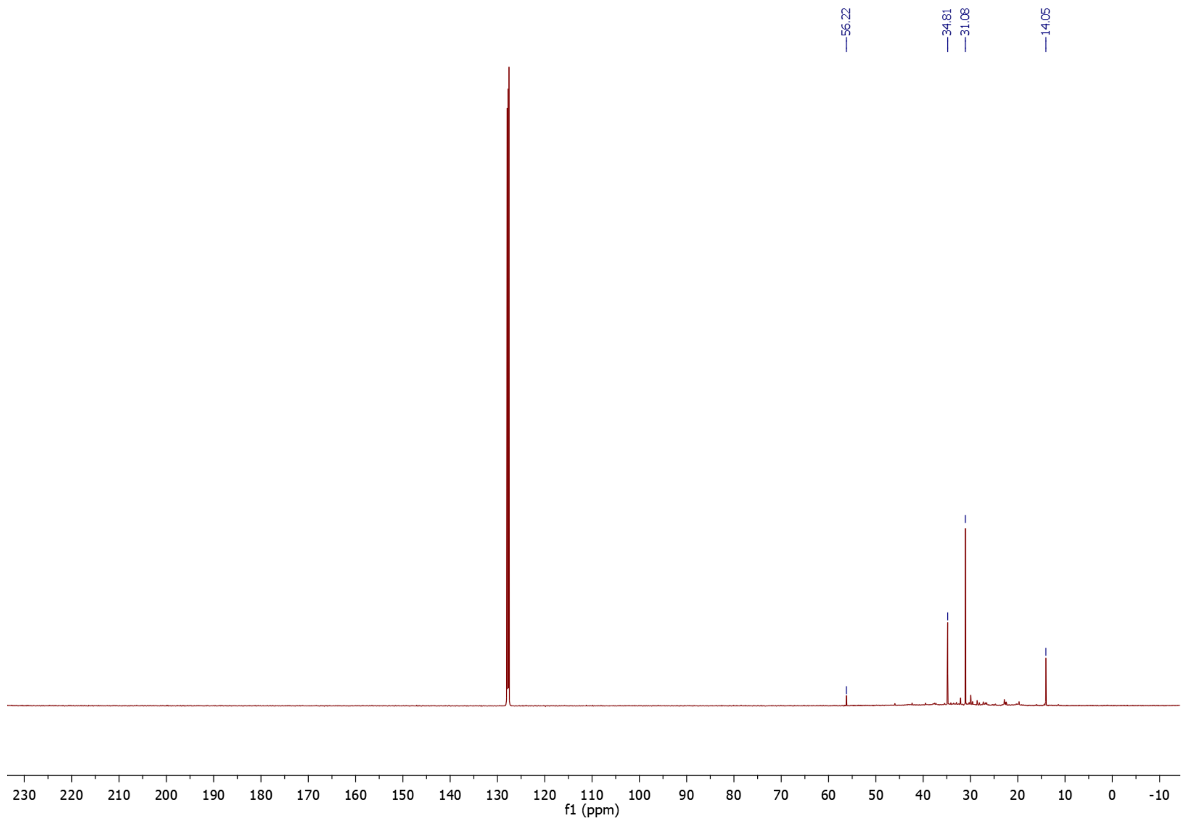

^{11}B-NMR (96 MHz, benzene-d_6) of **2b**

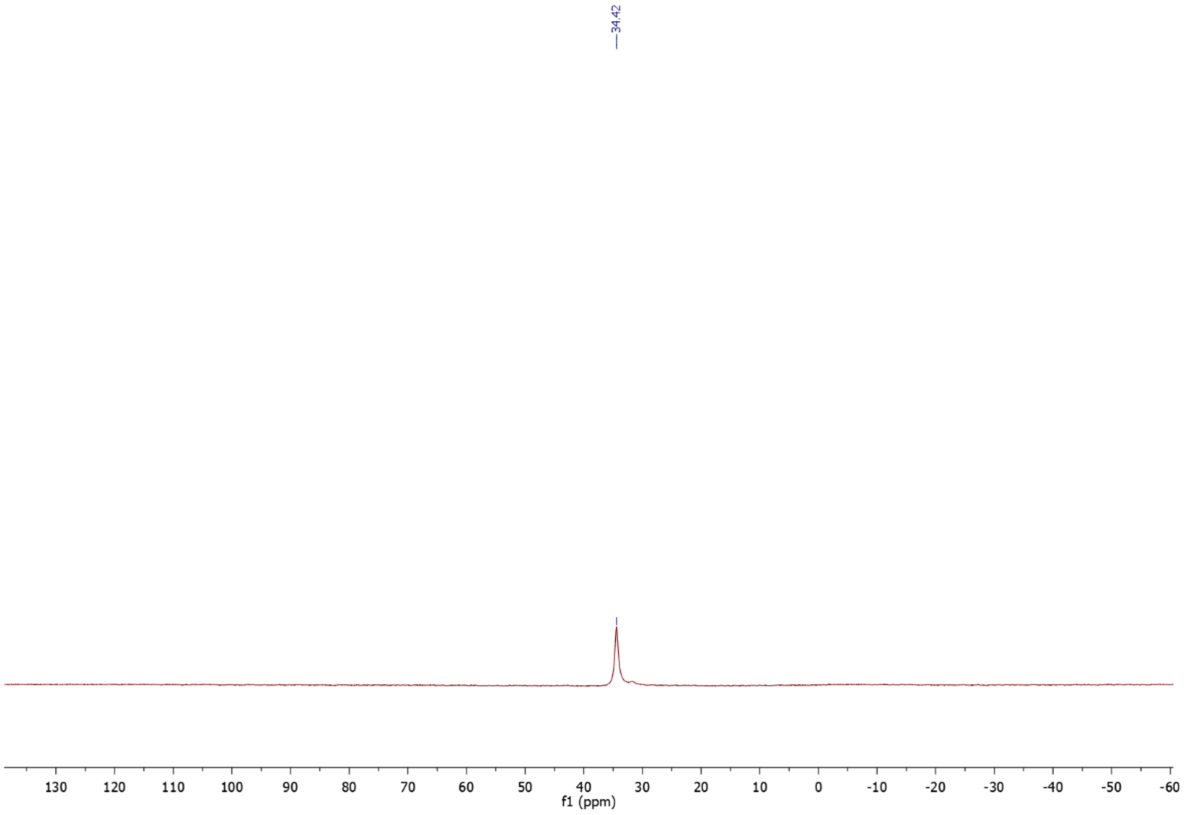

^{1}H-NMR (300 MHz, $CDCl_3$) of **4**

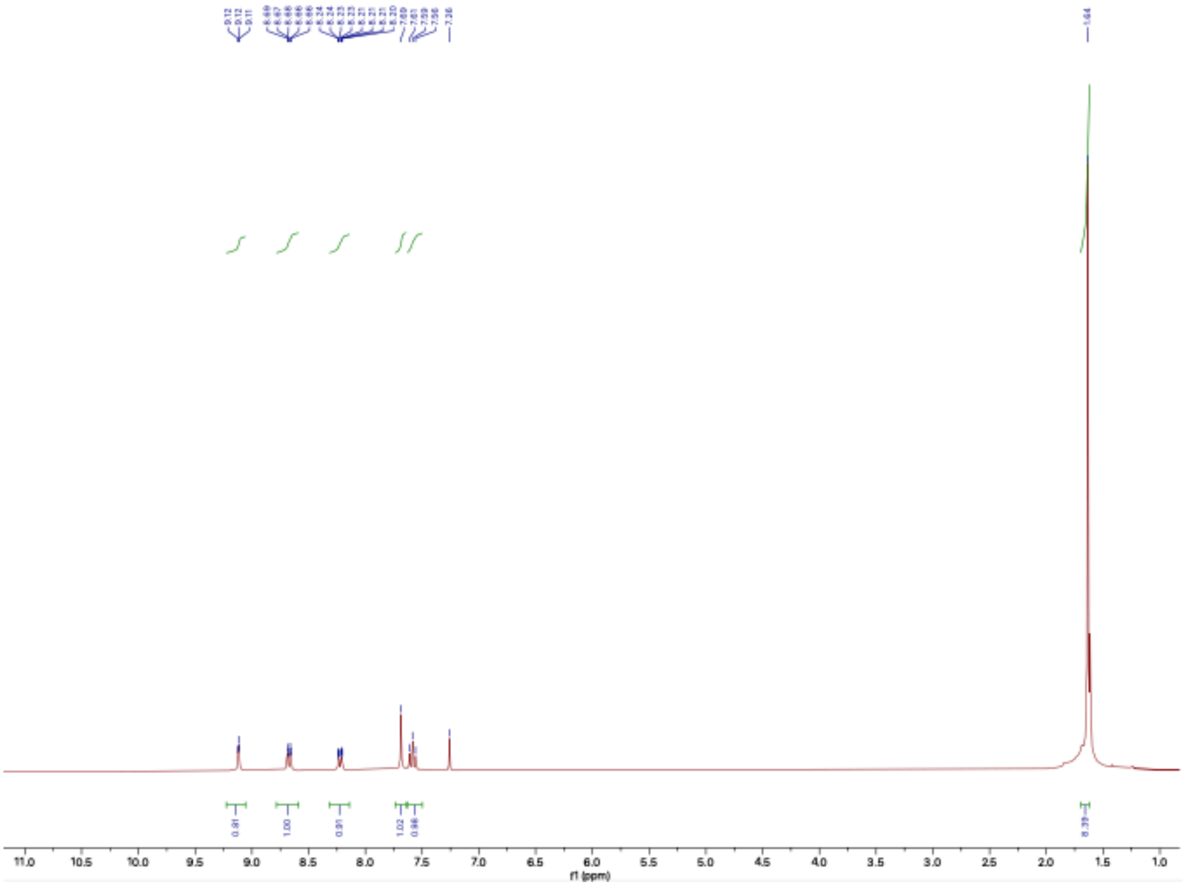

^{1}H-NMR (400 MHz, $CDCl_3$) of **S1**

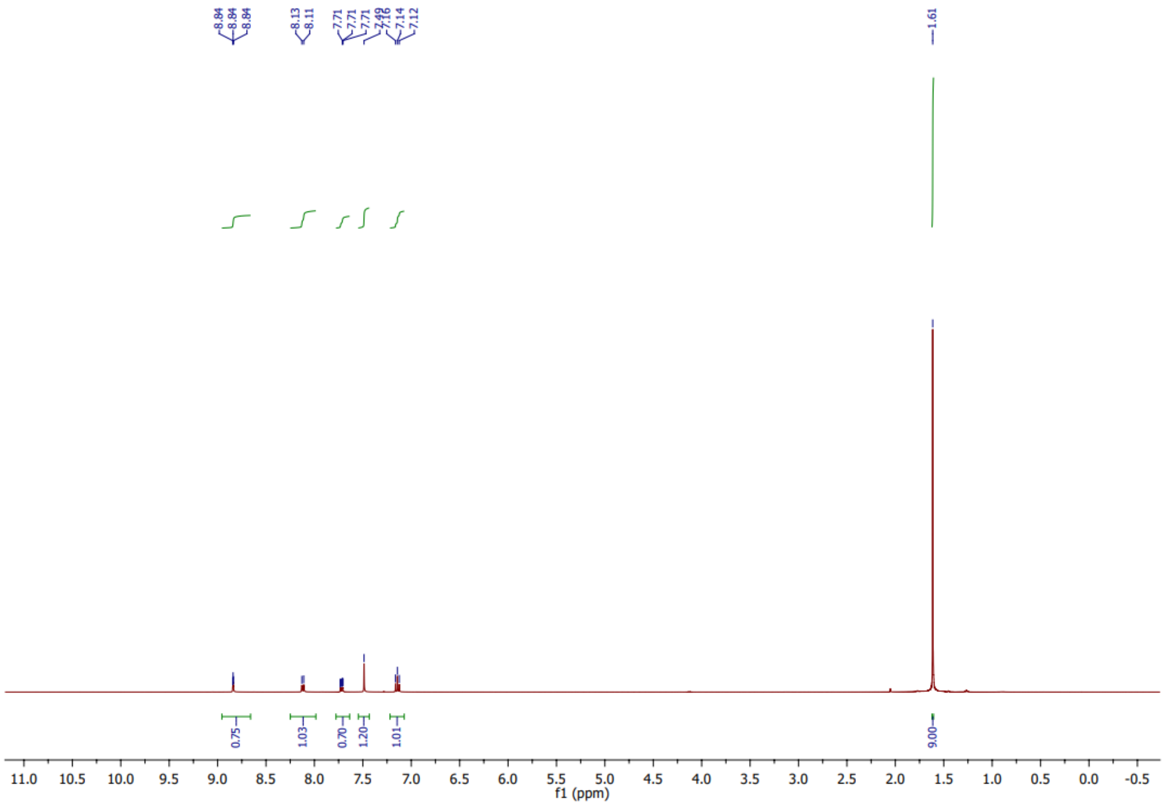

$^{13}C\{^{1}H\}$**-NMR** (101 MHz, $CDCl_3$) of **S1**

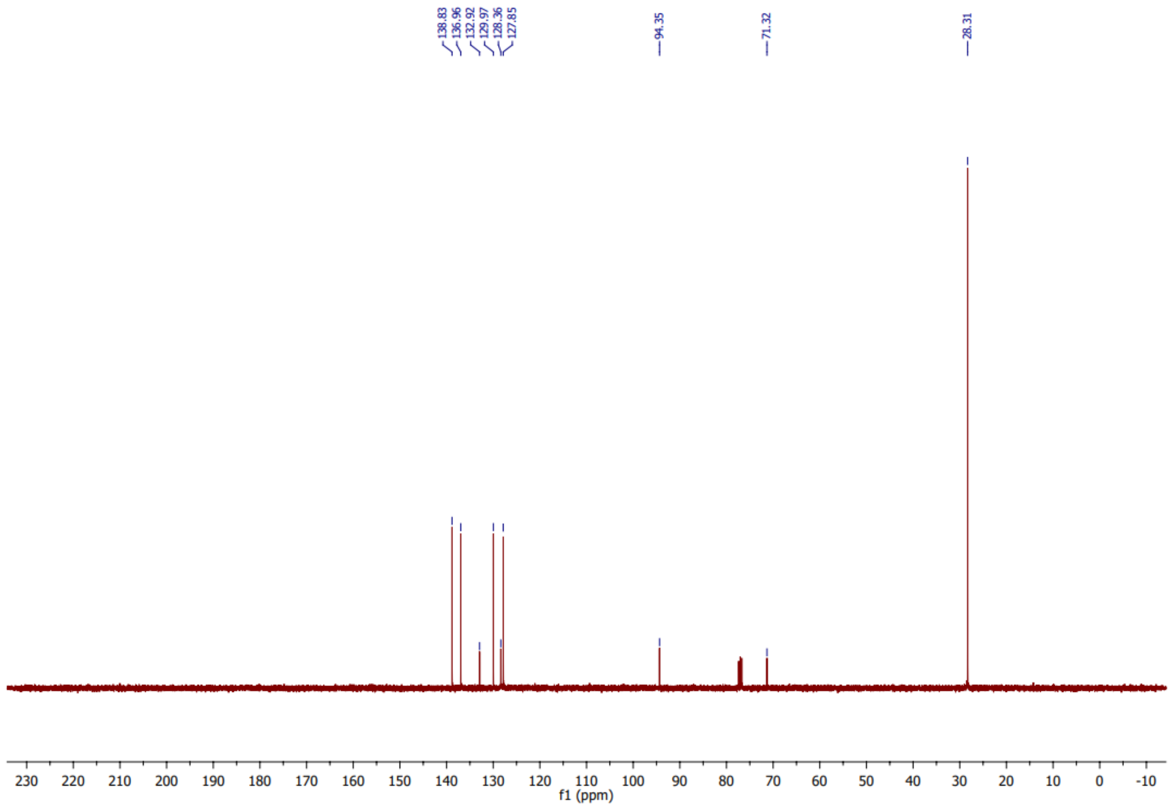

^{1}H-NMR (300 MHz, benzene-d_6) of **1a**

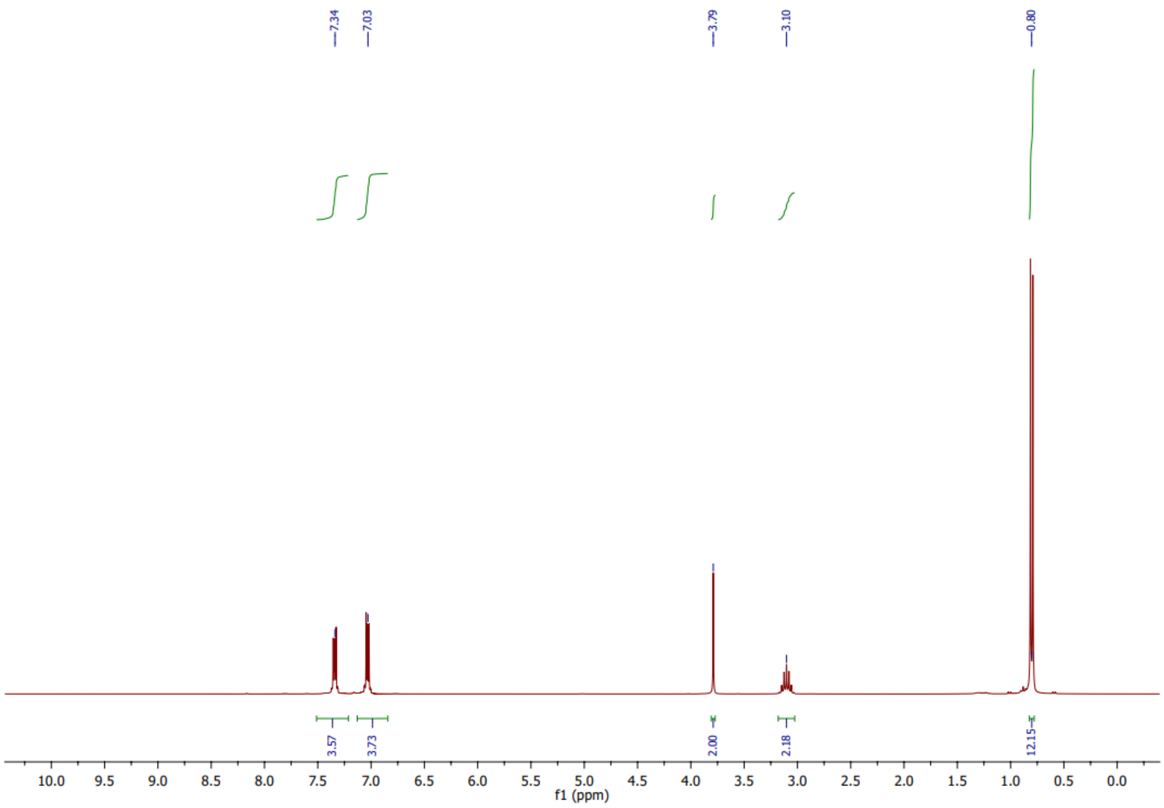

^{13}C{^{1}H}-NMR (126 MHz, benzene-d_6) of **1a**

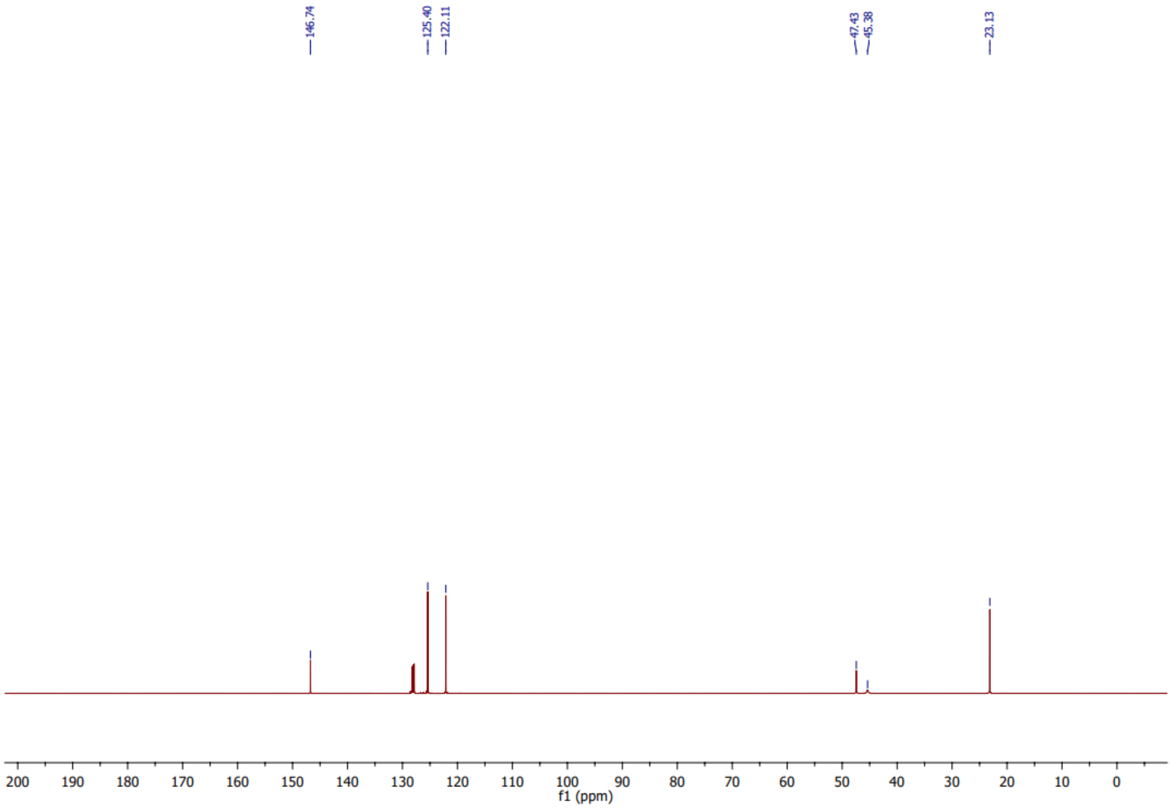

^{11}**B-NMR** (96 MHz, benzene-d_6) of **1a**

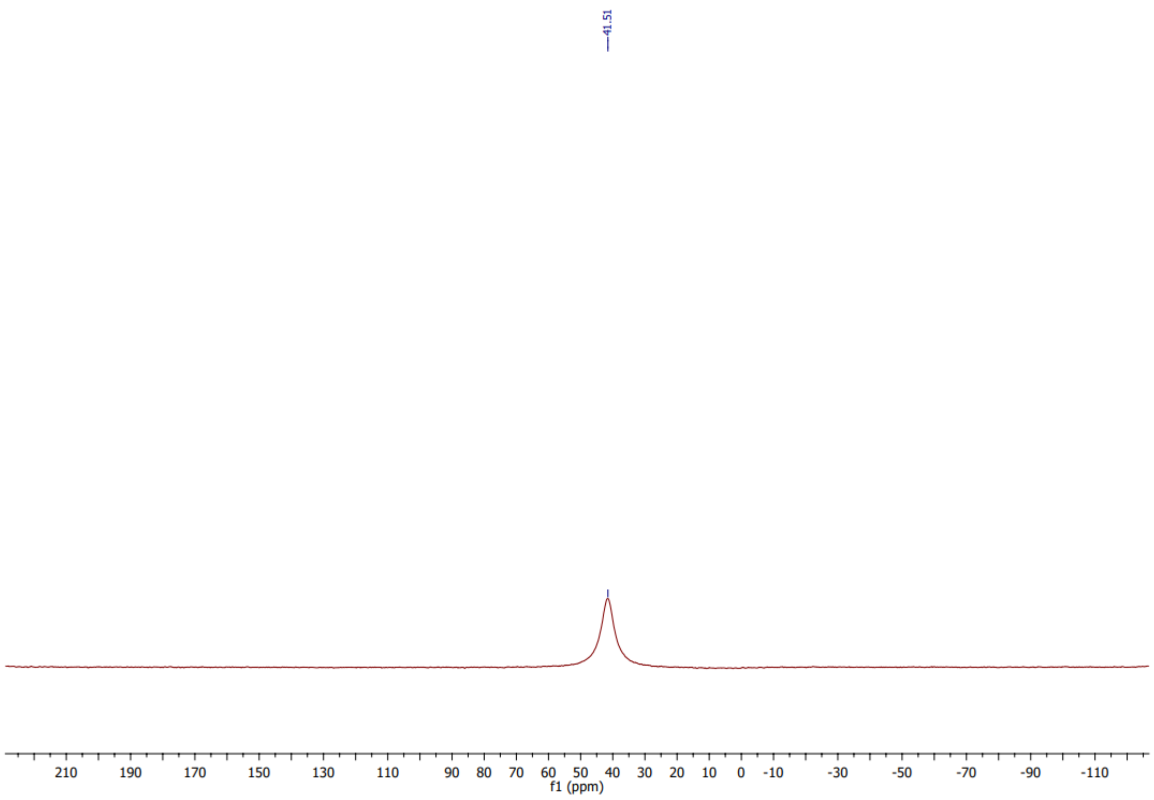

^{1}H-NMR (300 MHz, benzene-d_6) of **1b**

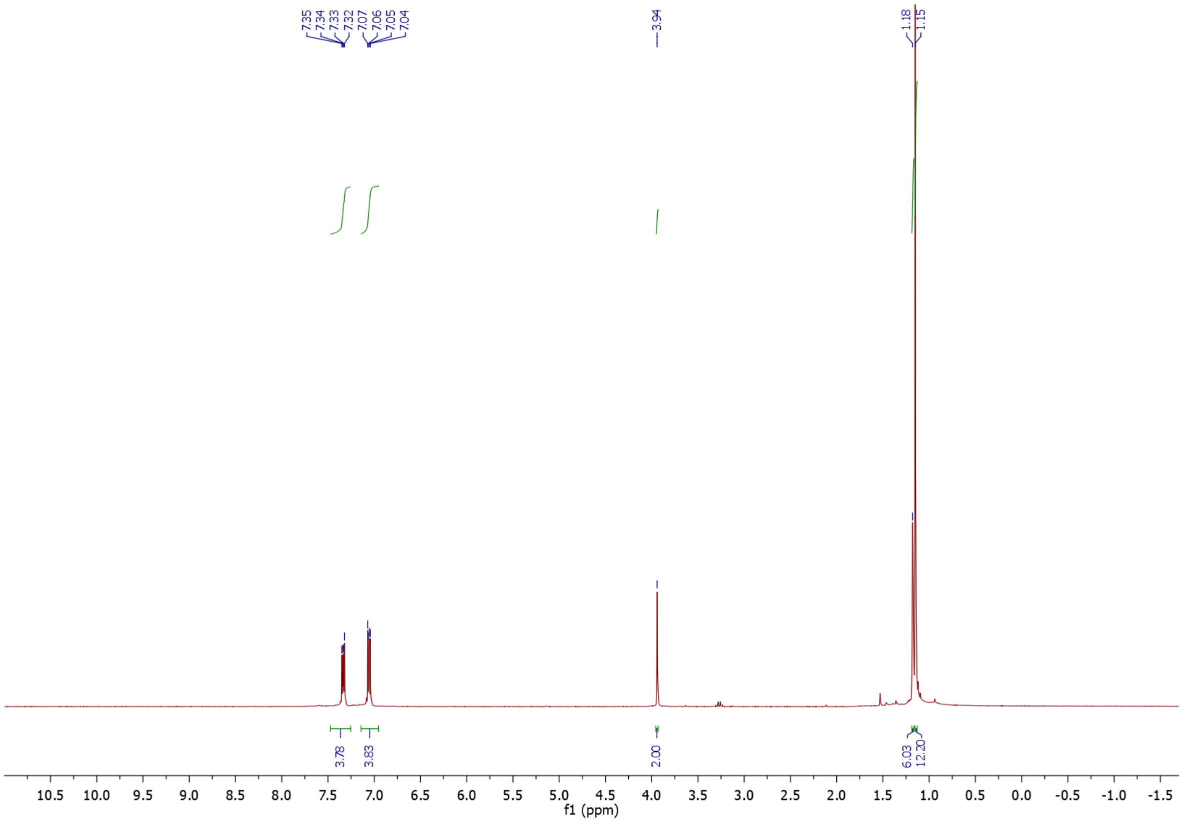

$^{13}C\{^{1}H\}$-NMR (126 MHz, benzene-d_6) of **1b**

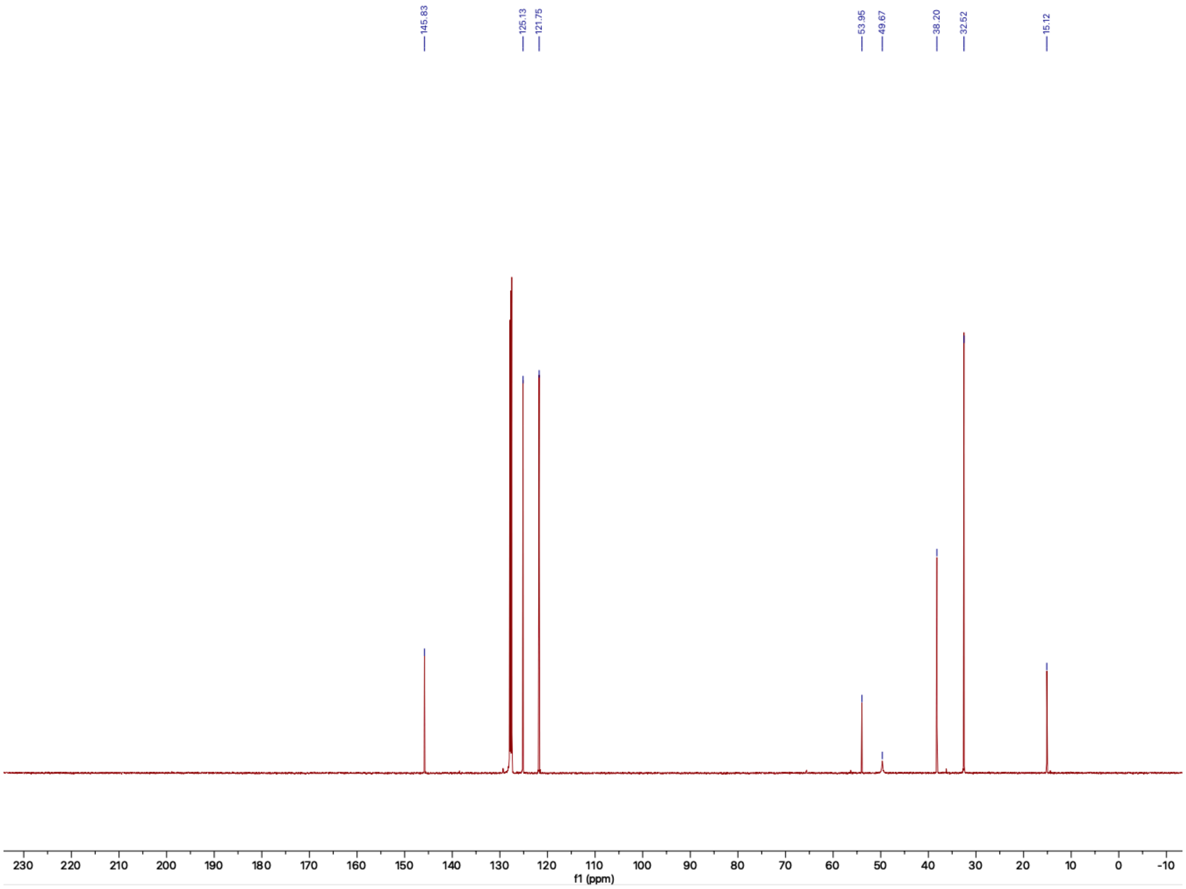

^{11}B-NMR (96 MHz, benzene-d_6) of **1b**

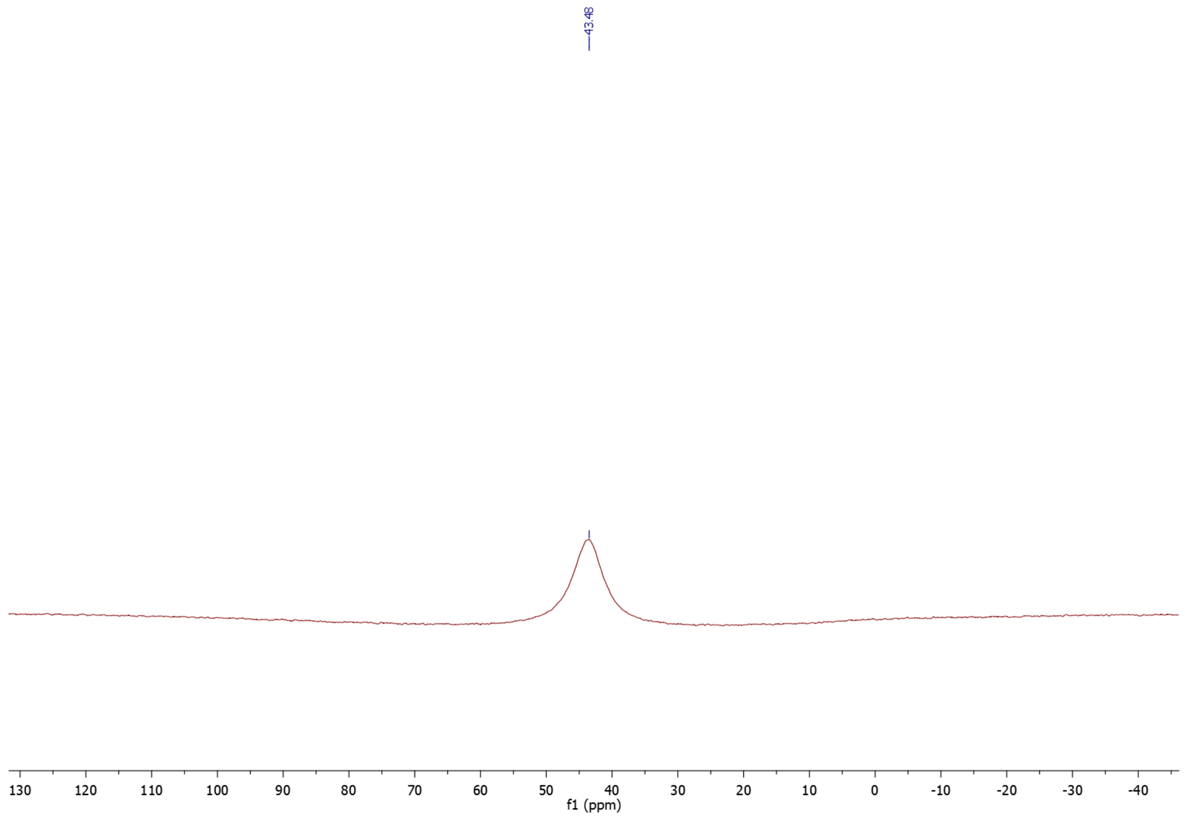

^{1}H-NMR (300 MHz, benzene-d_6) of **1c**

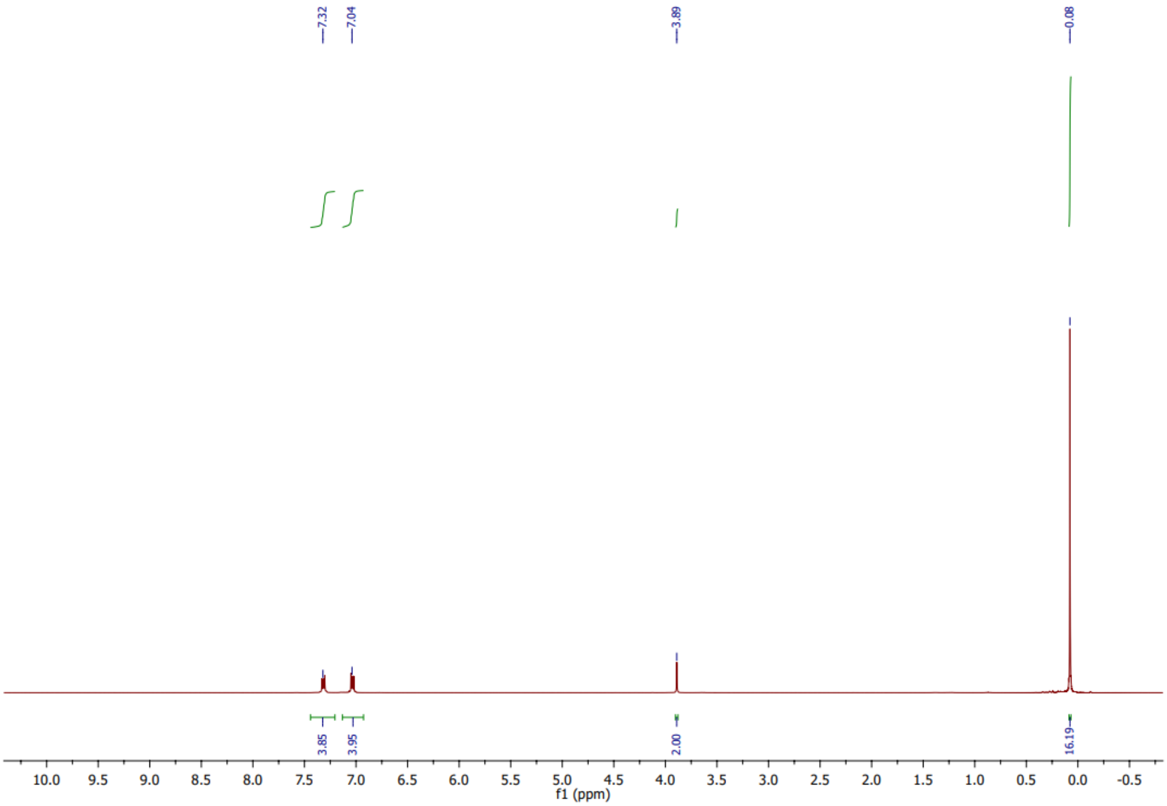

$^{13}C\{^{1}H\}$-NMR (126 MHz, $CDCl_3$) of **1c**

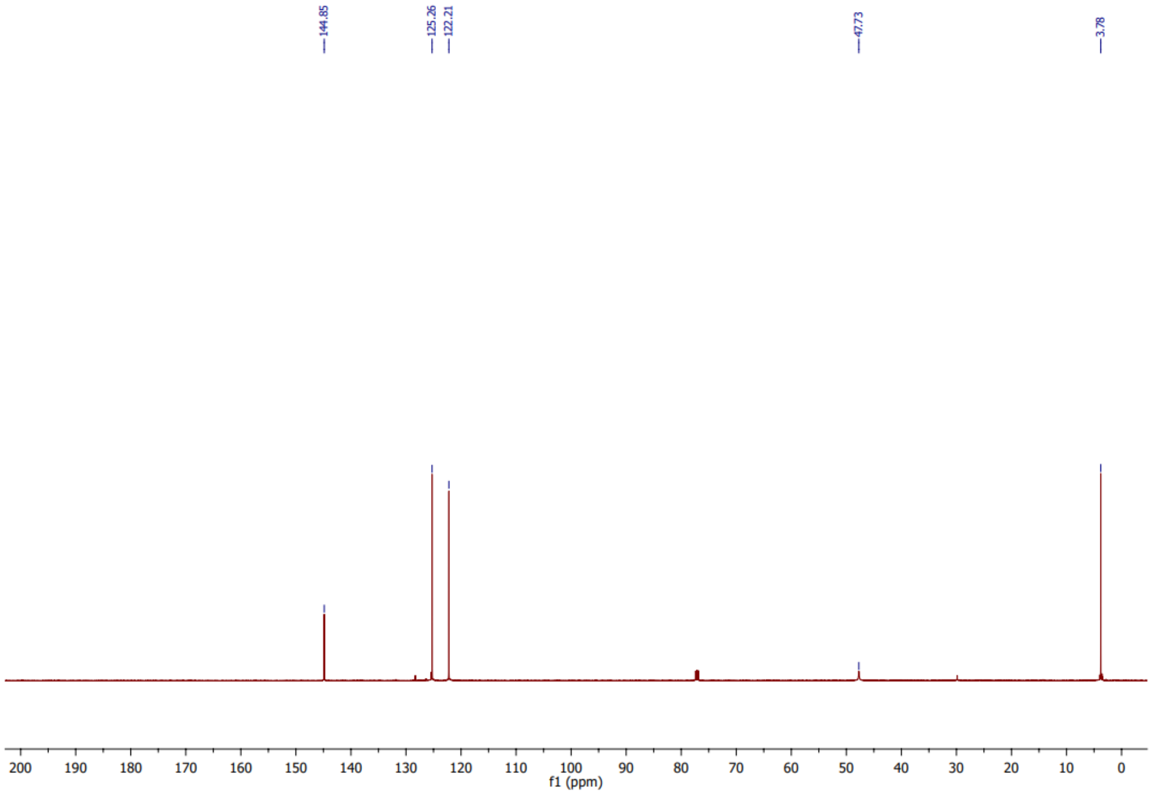

^{11}B-NMR (96 MHz, benzene-d_6) of **1c**

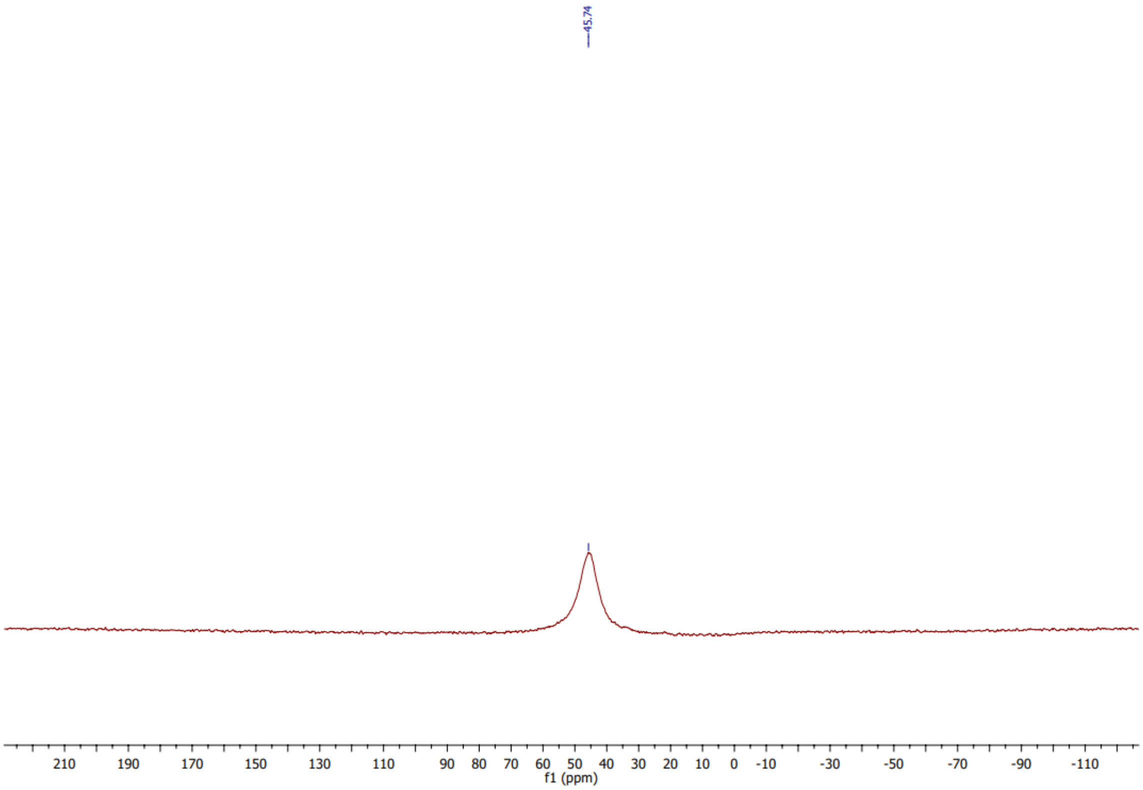

^{1}H-NMR (300 MHz, benzene-d_6) of **1d**

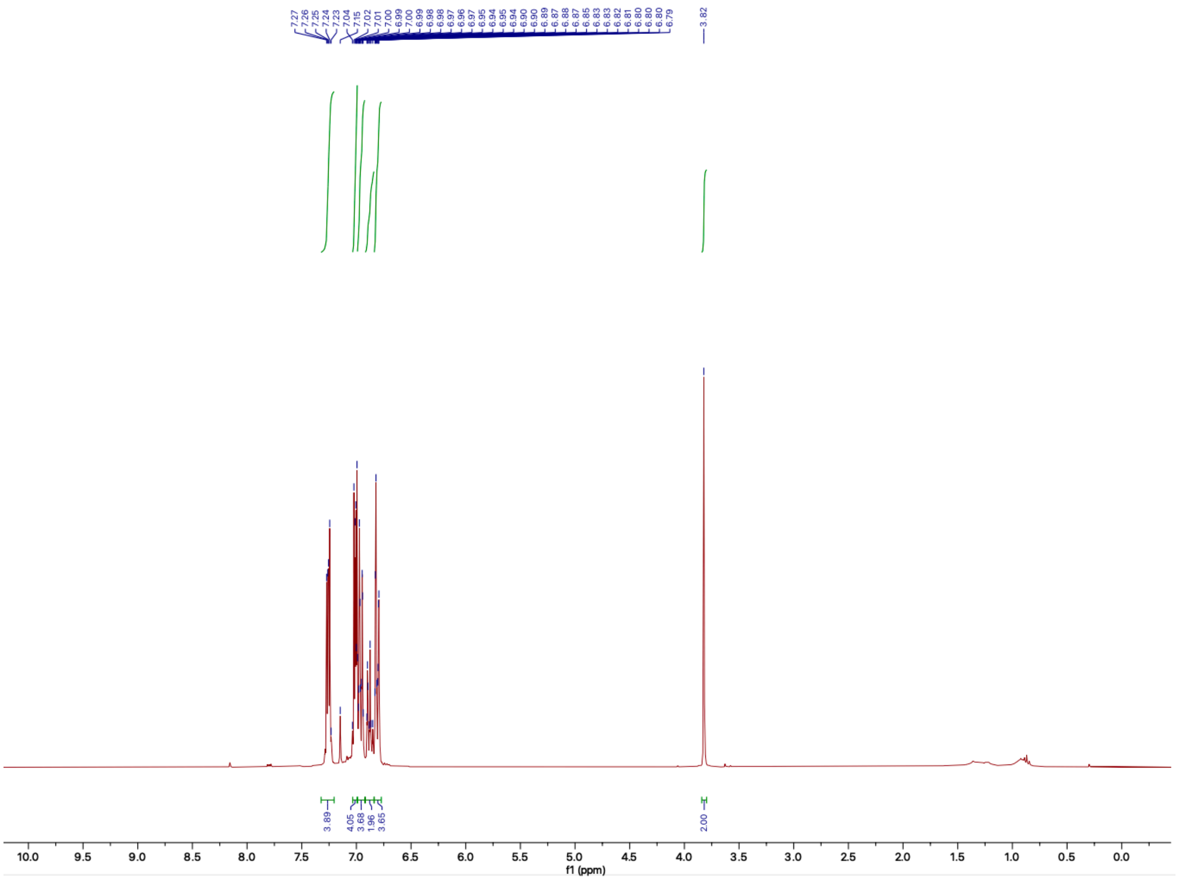

^{13}C{^{1}H}-NMR (126 MHz, benzene-d_6) of **1d**

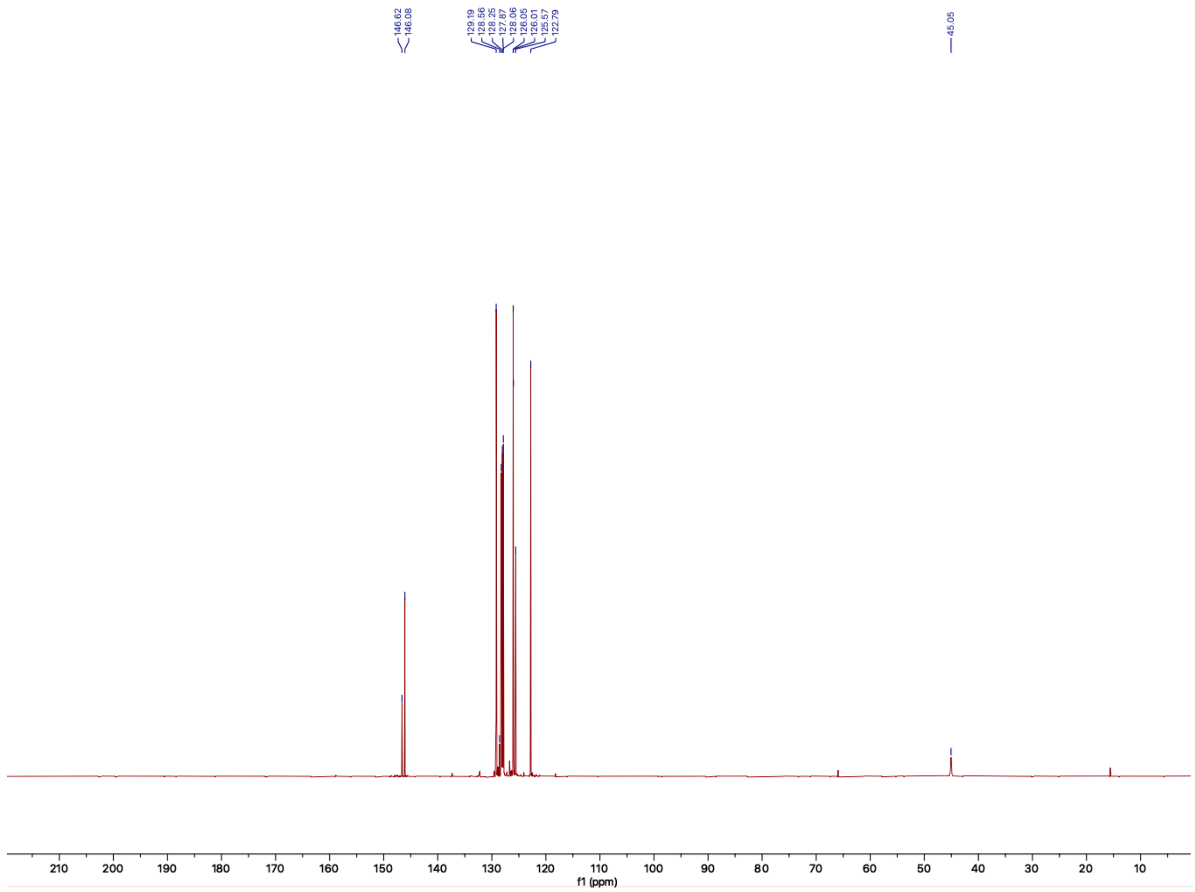

^{11}B-NMR (96 MHz, benzene-d_6) of **1d**

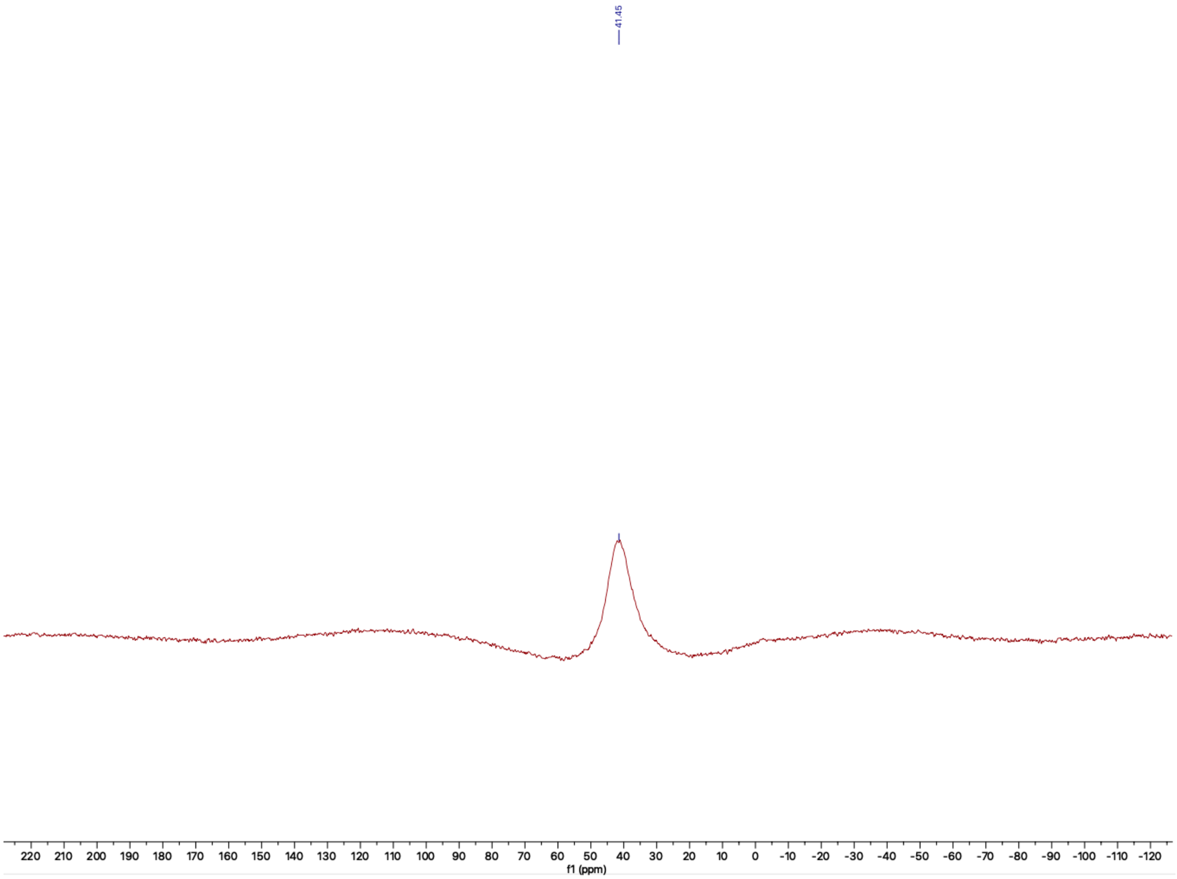

^{1}H-NMR (300 MHz, benzene-d_6) of **3c**

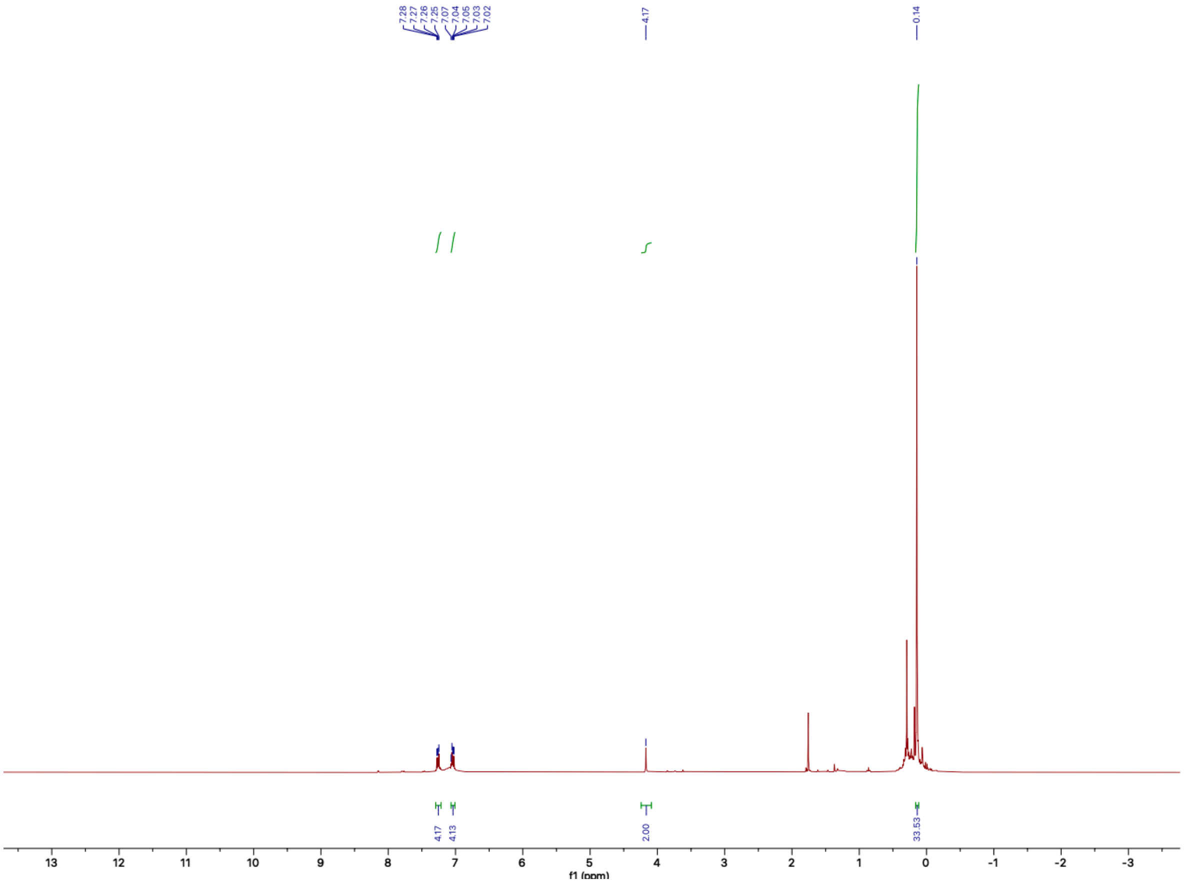

^{13}C{^{1}H}-NMR (125 MHz, $CDCl_3$) of **3c**

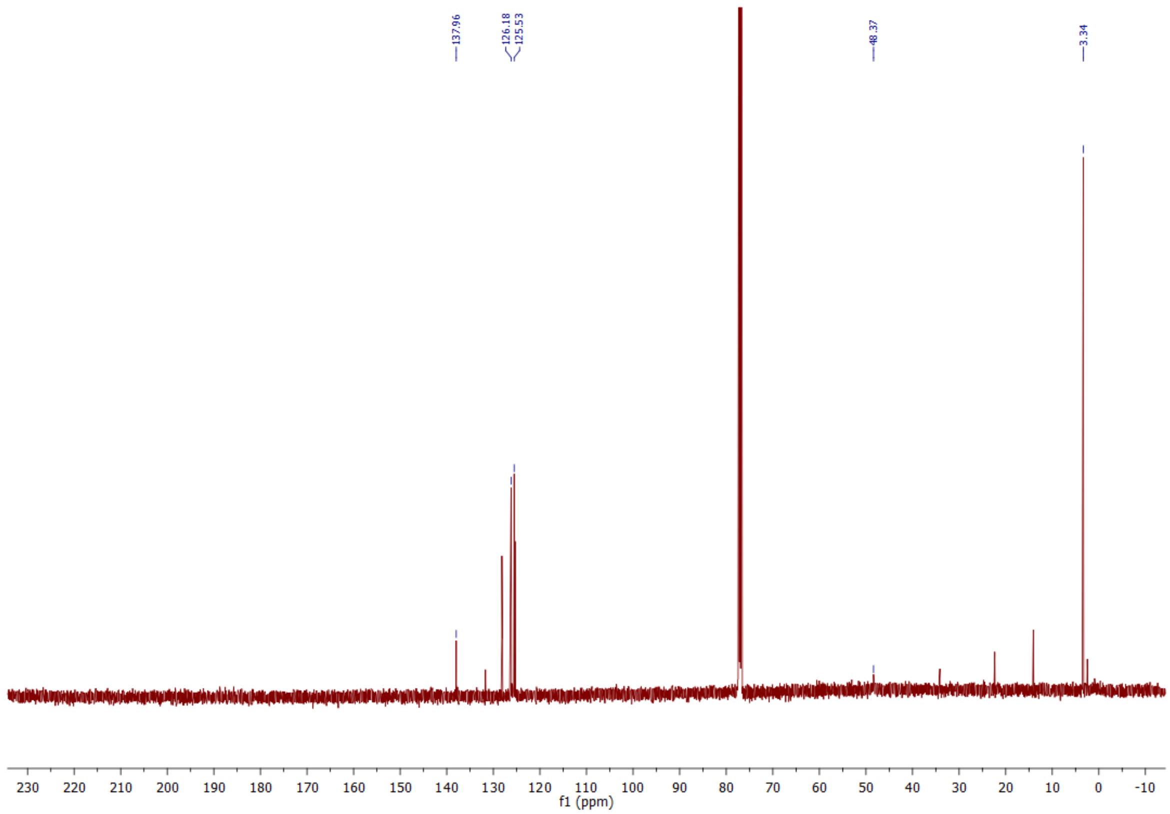

^{11}**B-NMR** (96 MHz, benzene-*d*$_6$) of **3c**

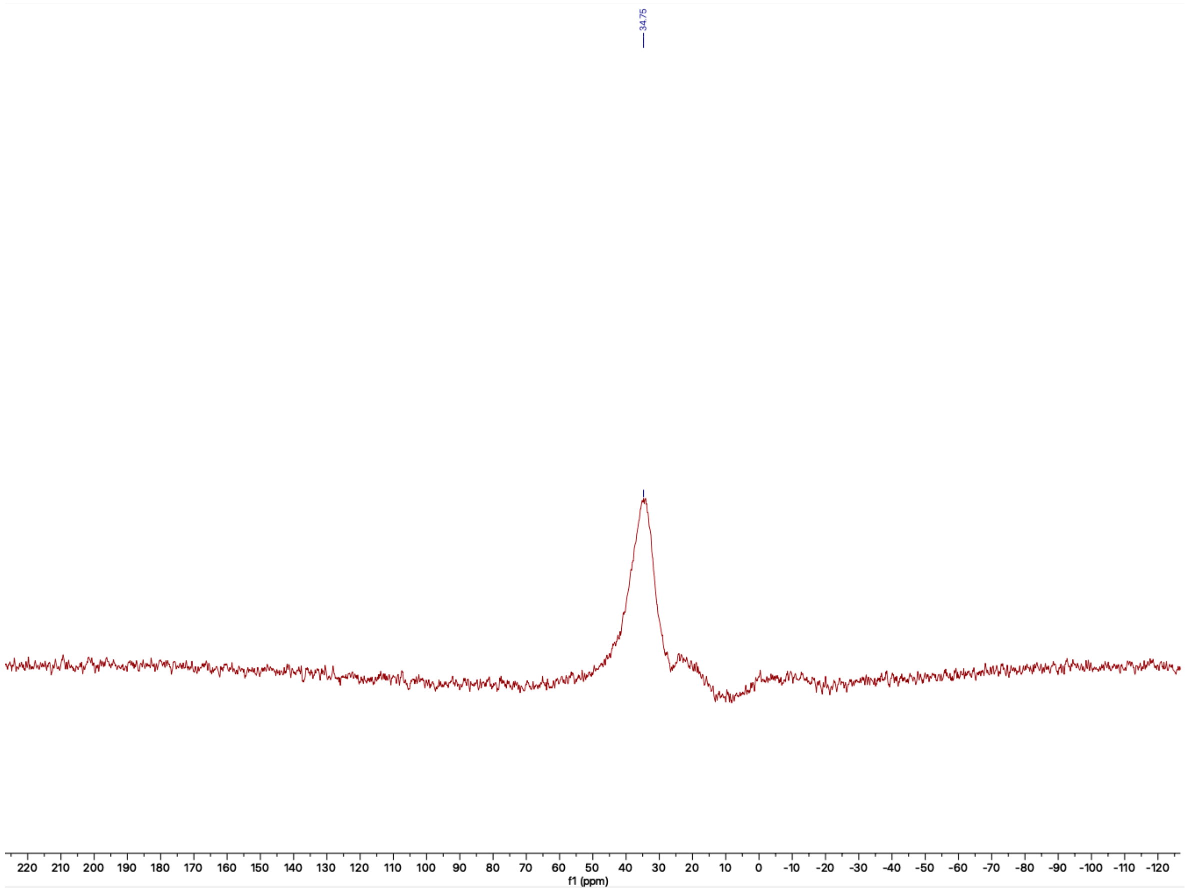

^{1}H-NMR (400 MHz, $CDCl_3$) of **5a**

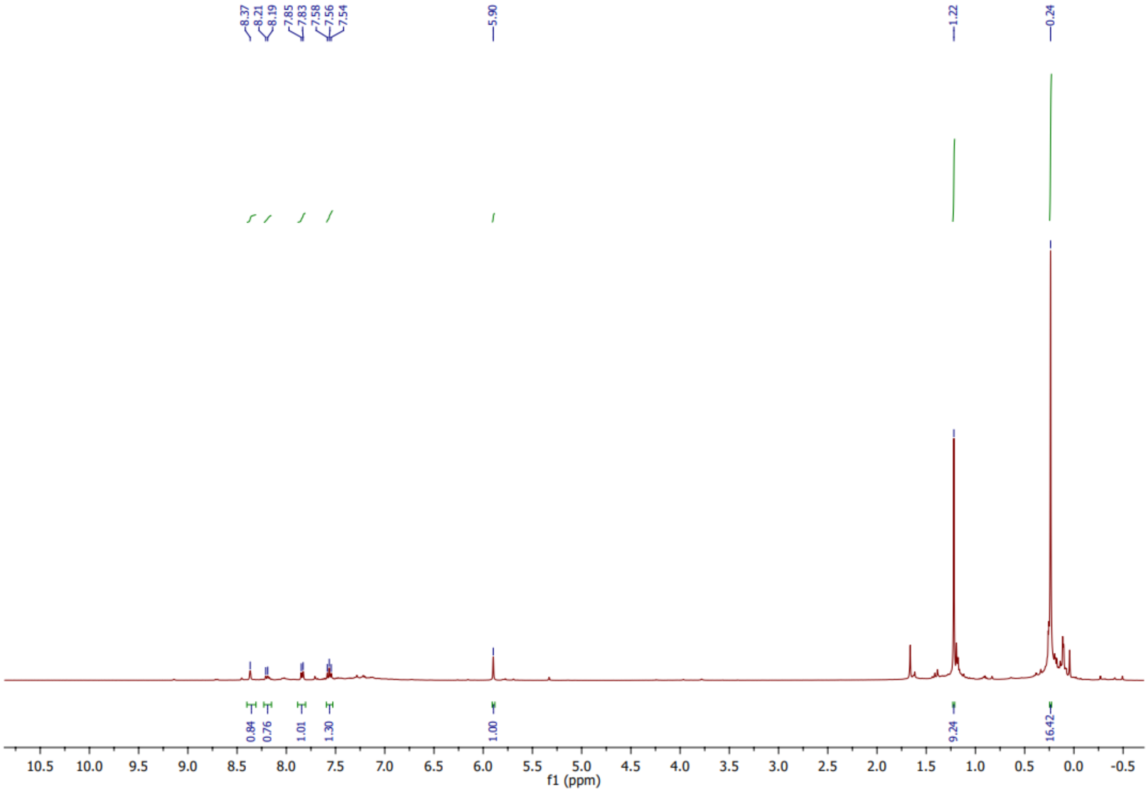

$^{13}C\{^{1}H\}$-NMR (101 MHz, $CDCl_3$) of **5a**

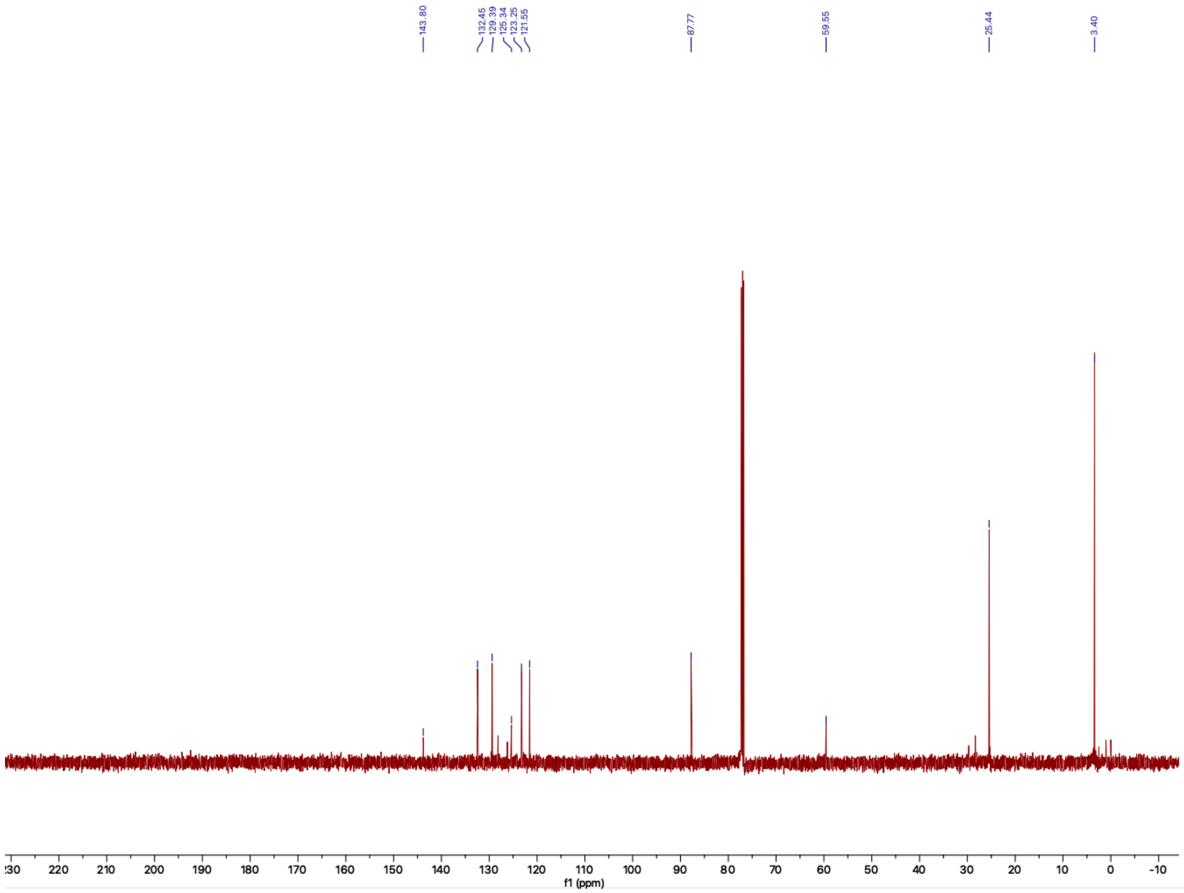

^{11}B-NMR (96 MHz, benzene-d_6) of **5a**

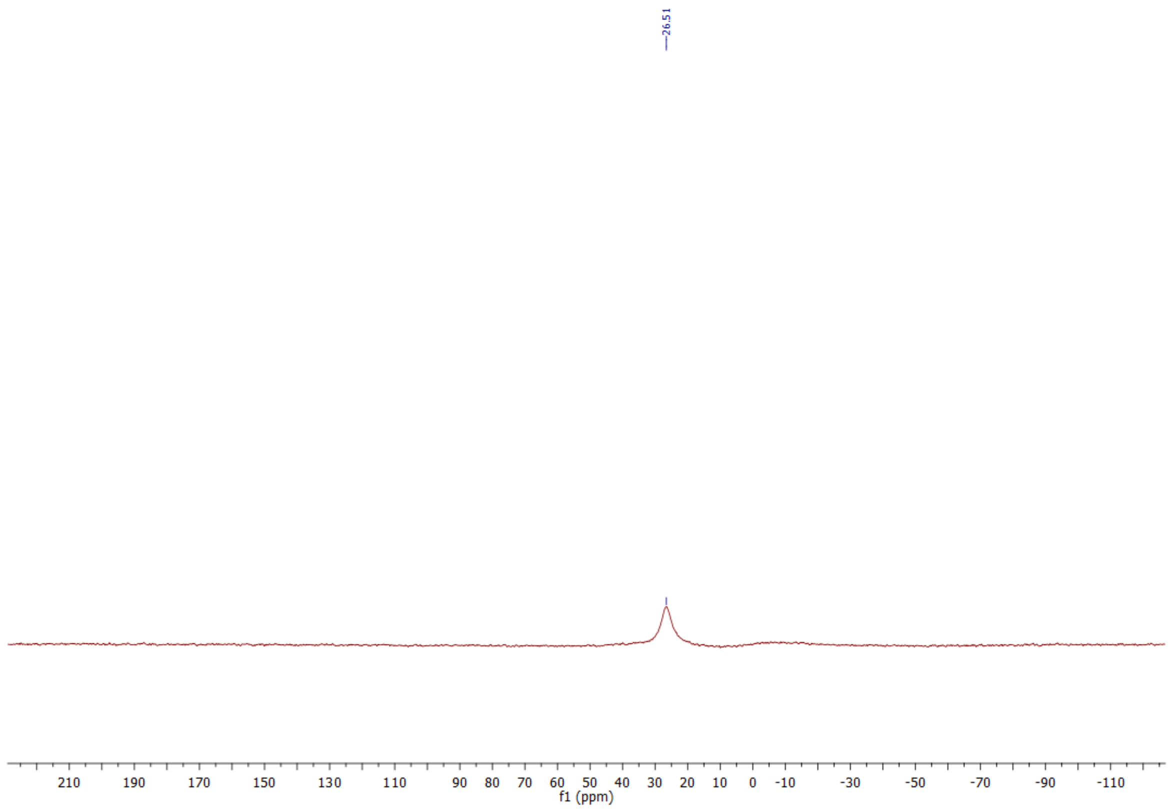

[1]**H-NMR** (300 MHz, $CDCl_3$) of **5b**

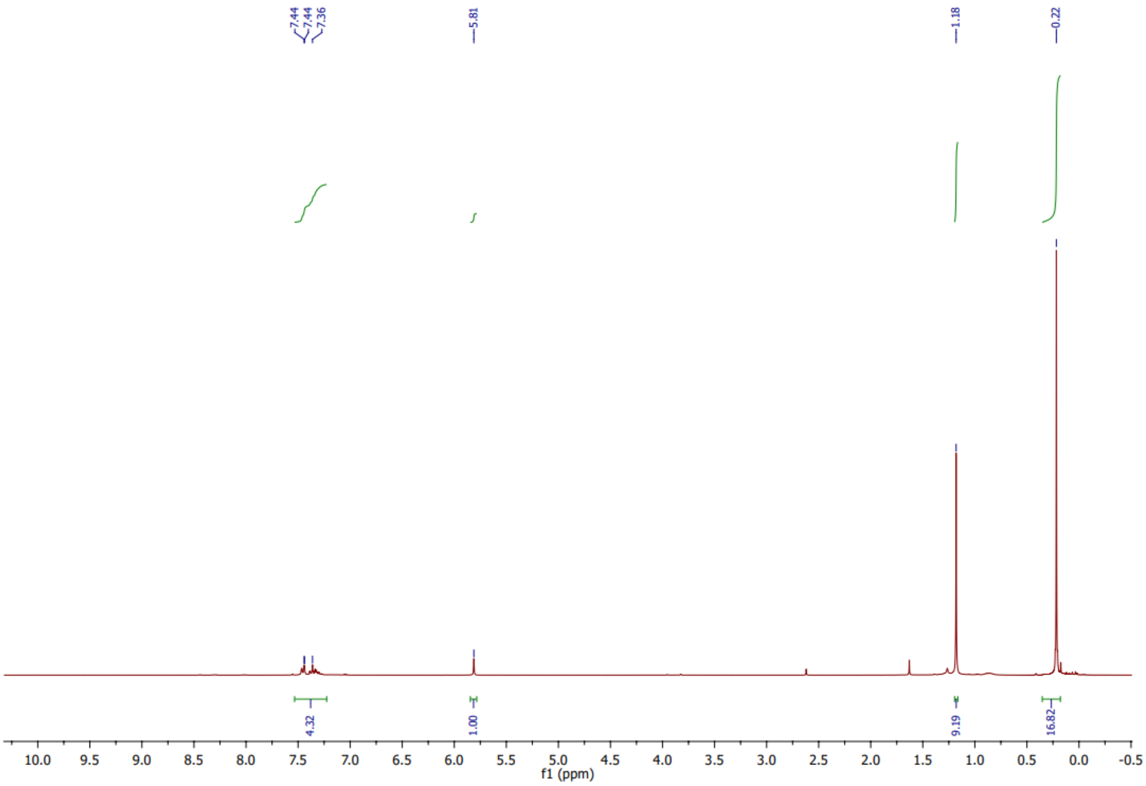

^{13}C{^{1}H}-NMR (125 MHz, $CDCl_3$) of **5b**

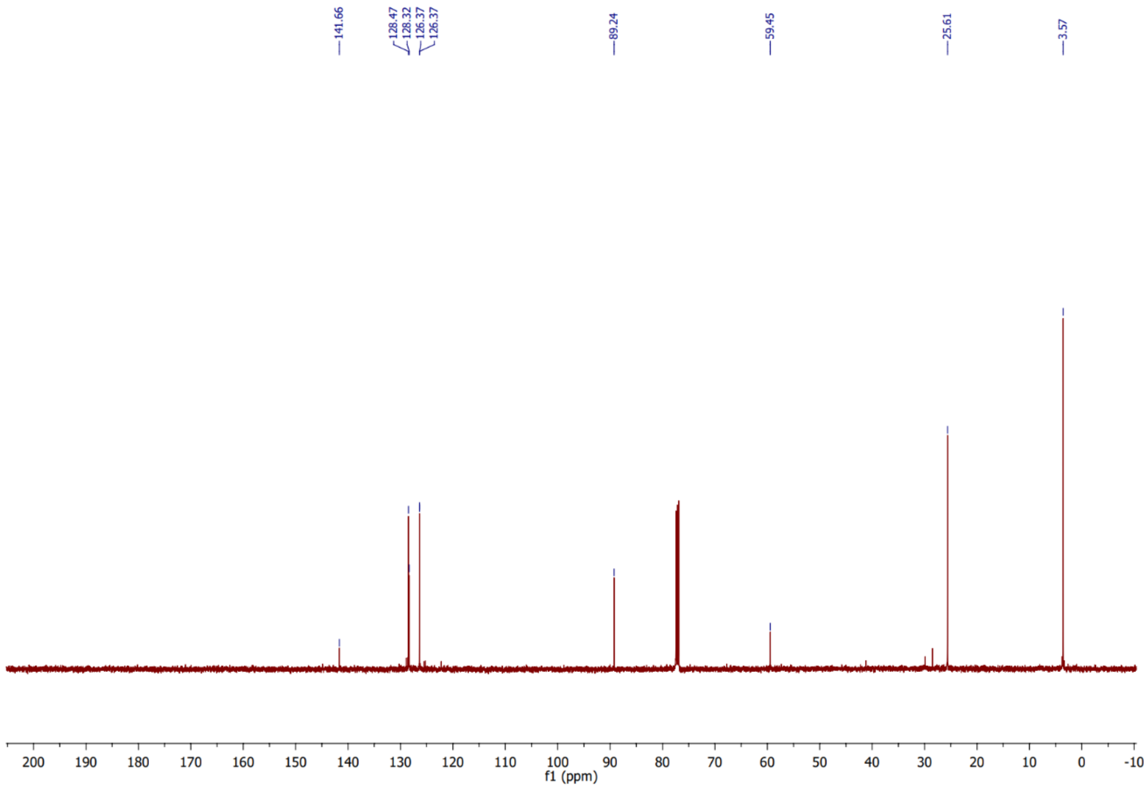

^{11}**B-NMR** (96 MHz, benzene-d_6) of **5b**

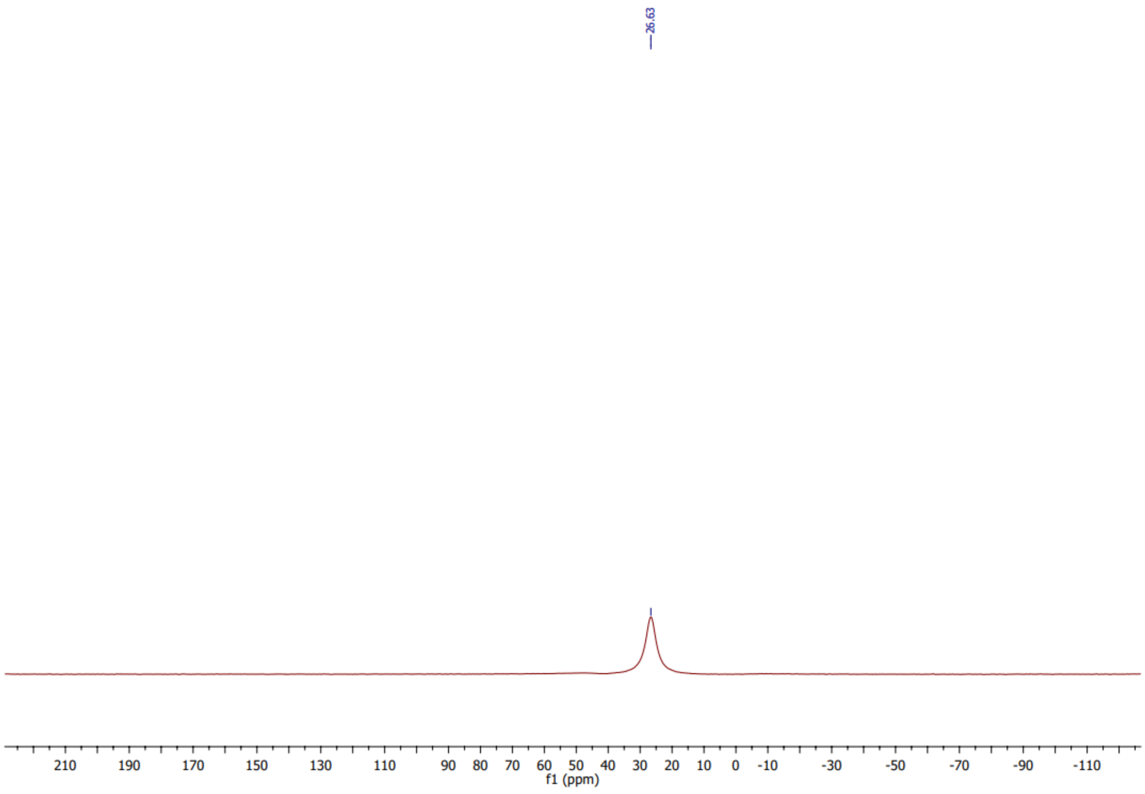

^{1}**H-NMR** (400 MHz, $CDCl_3$) of **5c**

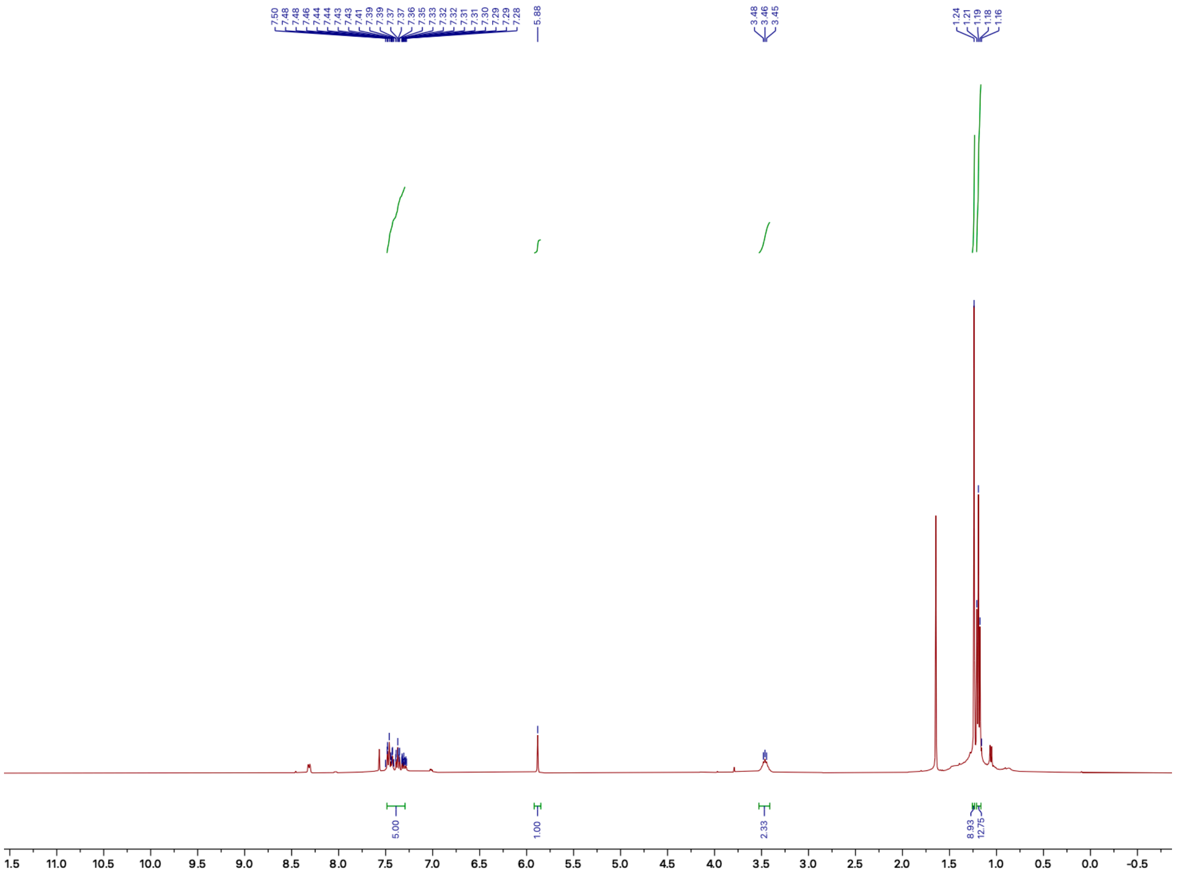

$^{13}C\{^1H\}$**-NMR** (101 MHz, $CDCl_3$) of **5c**

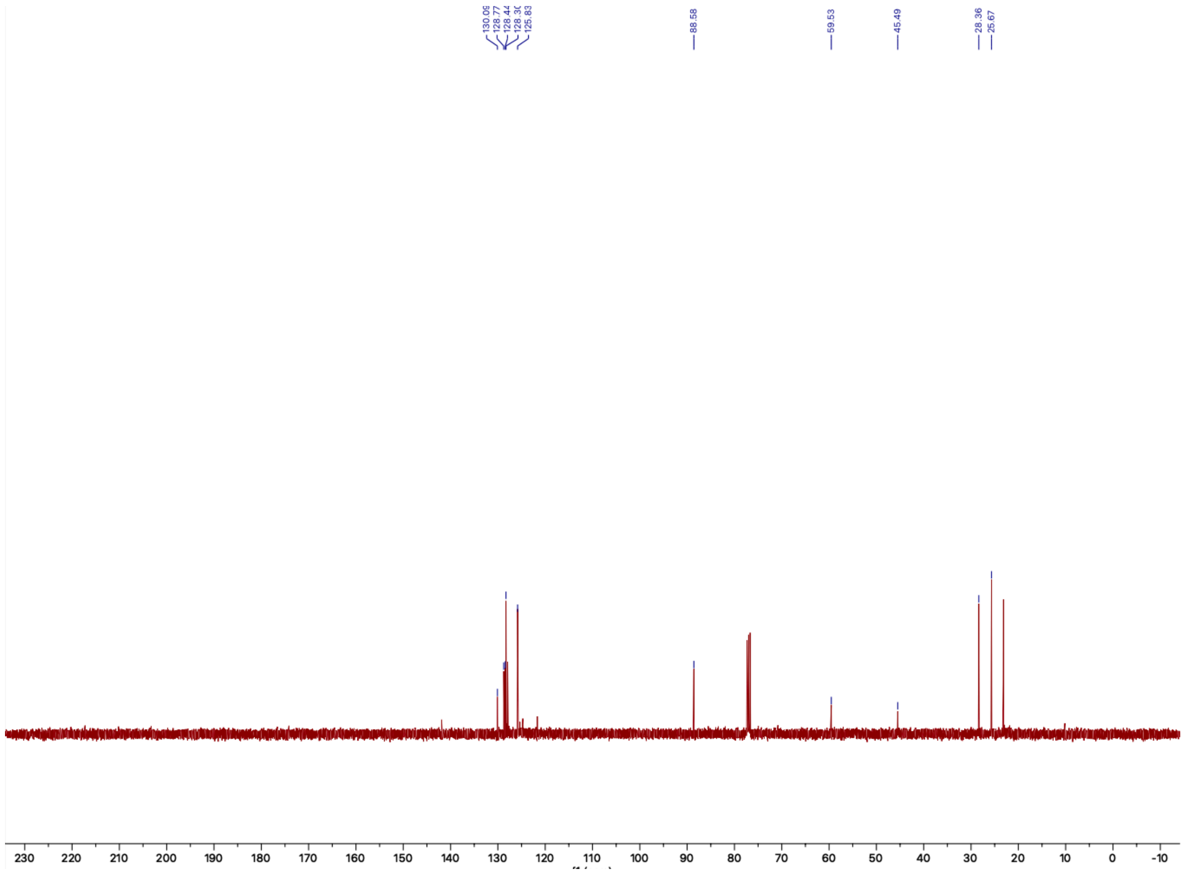

[11]**B-NMR** (benzene-d6, 96 MHz) of **5c**

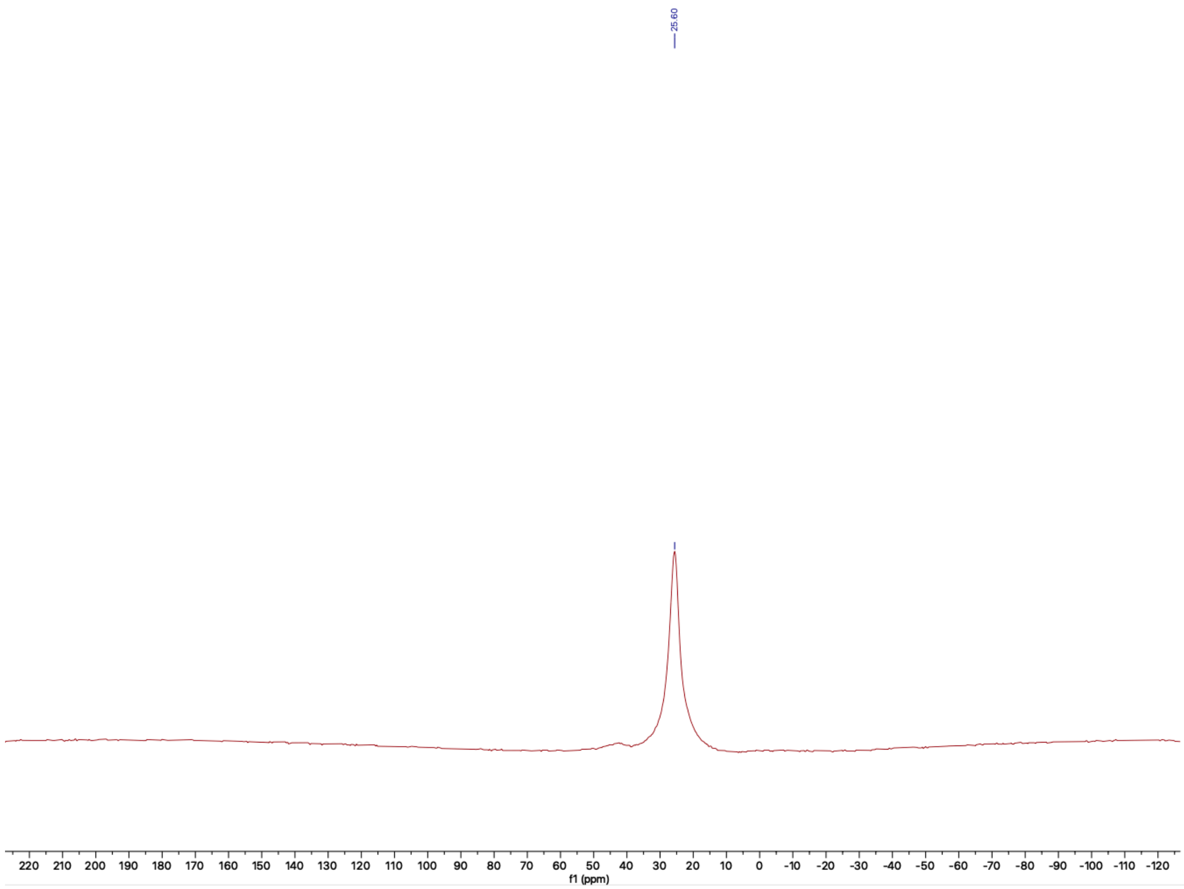

[1]**H-NMR** (400 MHz, $CDCl_3$) of **S2**

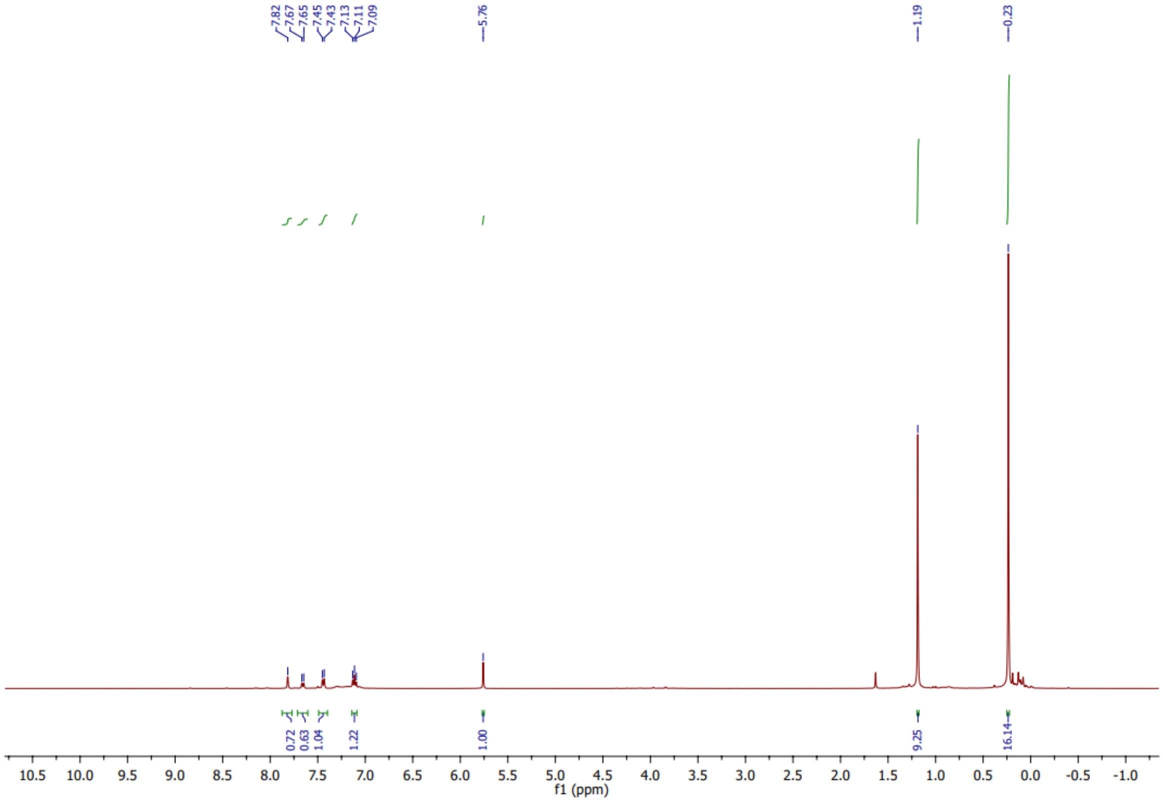

$^{13}C\{^1H\}$-NMR (101 MHz, $CDCl_3$) of **S2**

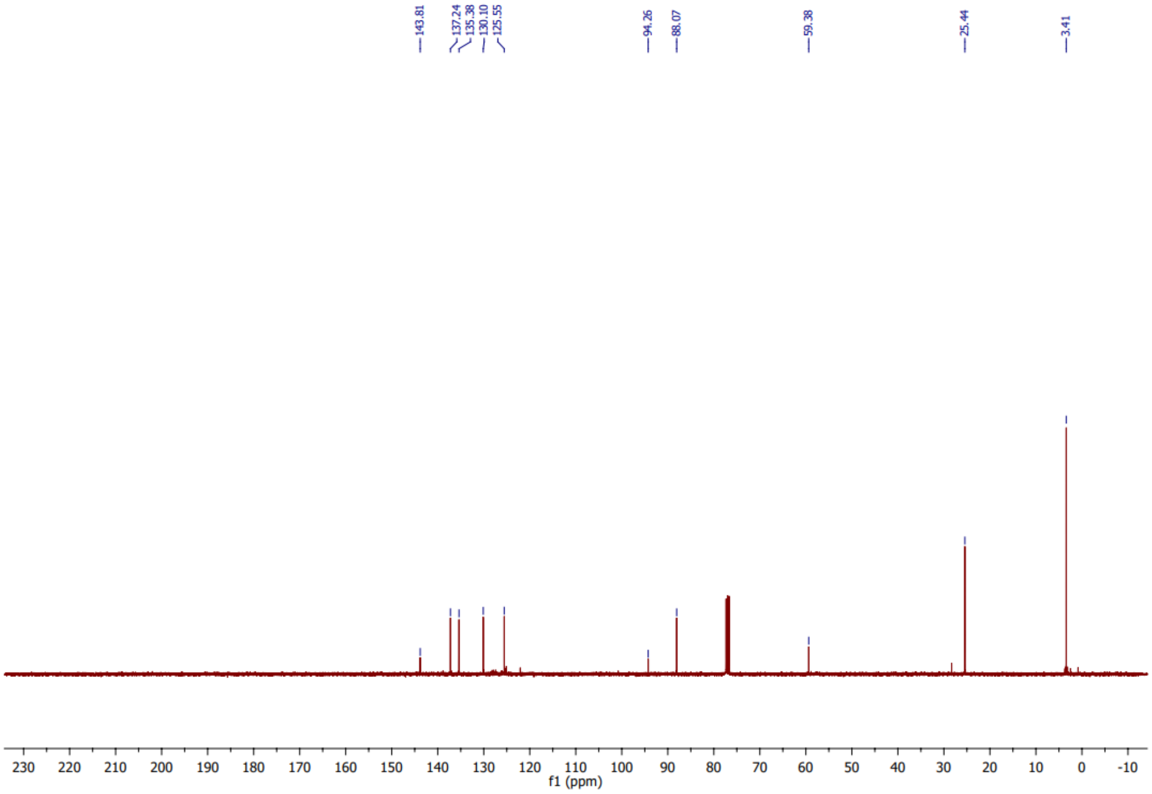

^{11}B-NMR (96 MHz, benzene-d_6) of **S2**

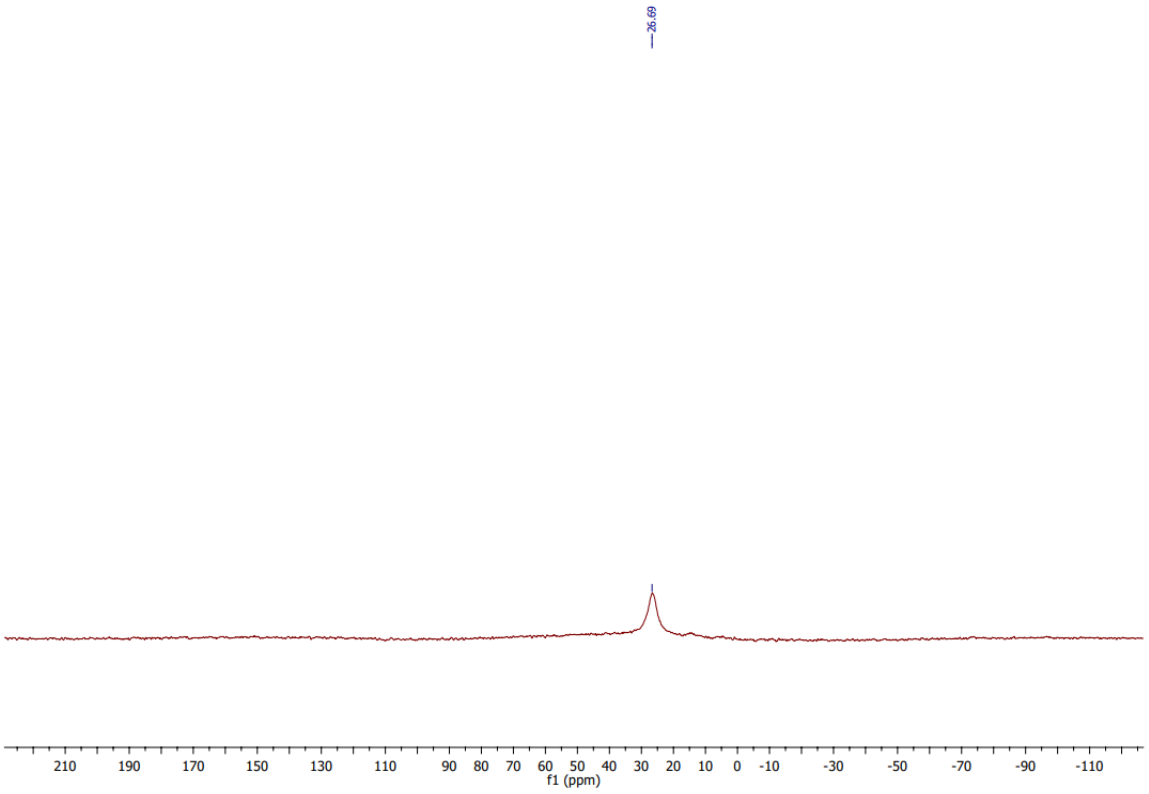

^{1}H-NMR (300 MHz, benzene-d_6) of **6a**

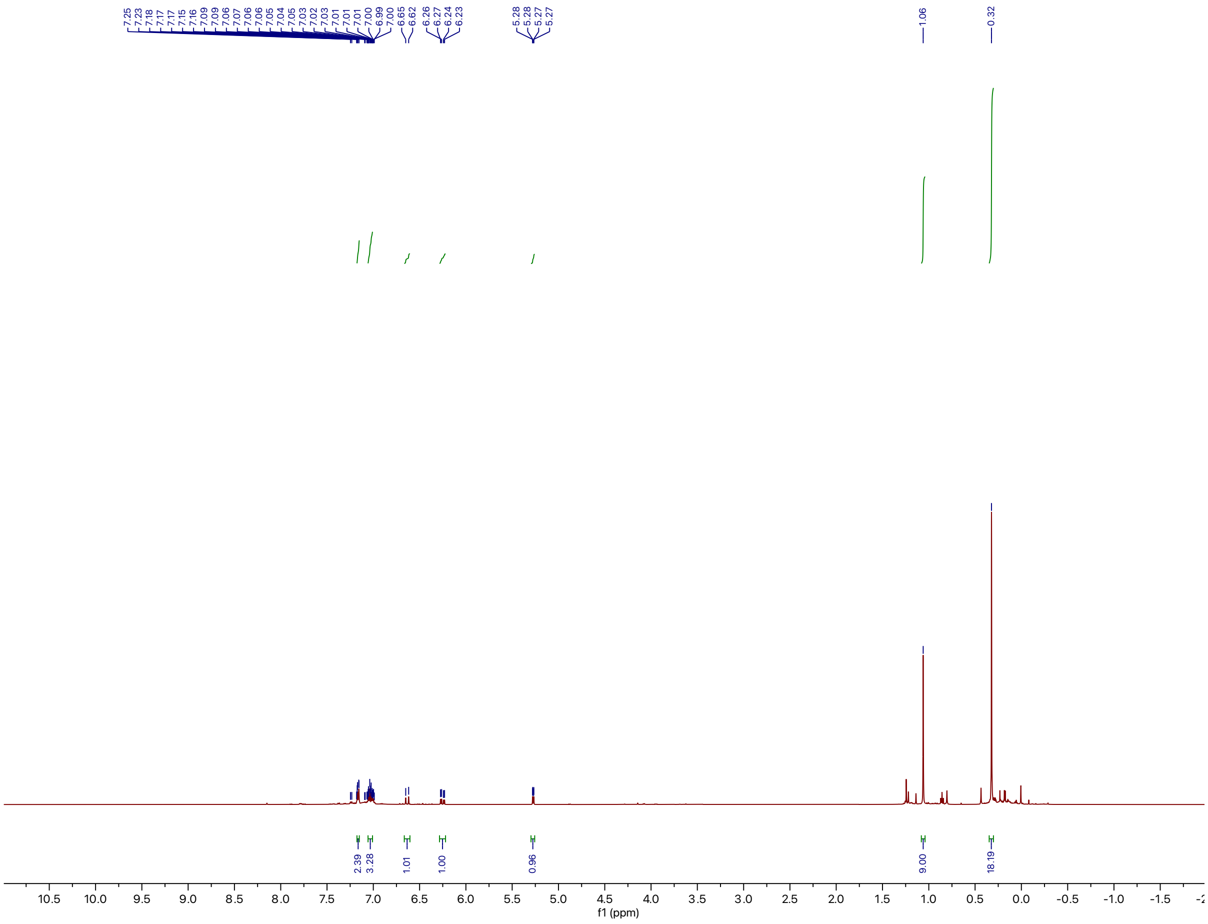

$^{13}C\{^{1}H\}$**-NMR** (75 MHz, $CDCl_3$) of **6a**

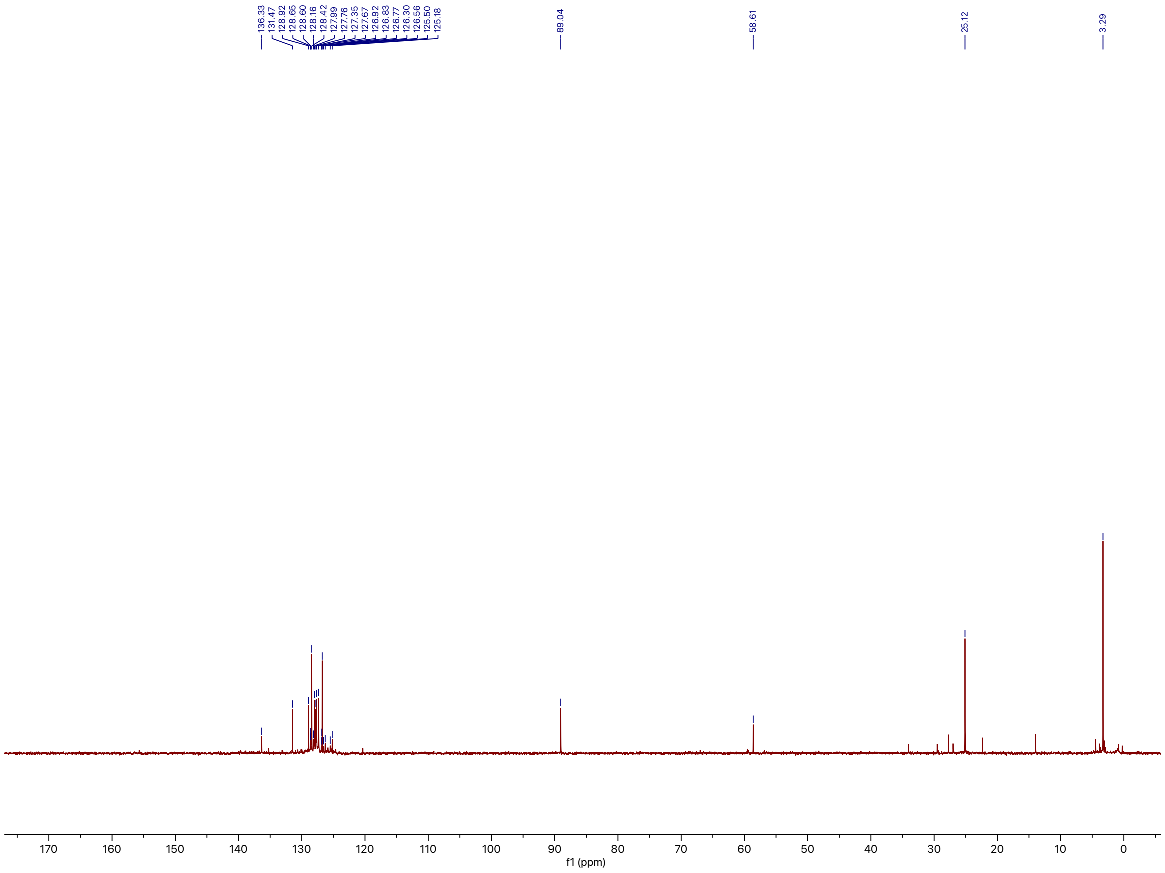

^{11}B-NMR (96 MHz, benzene-d_6) of **6a**

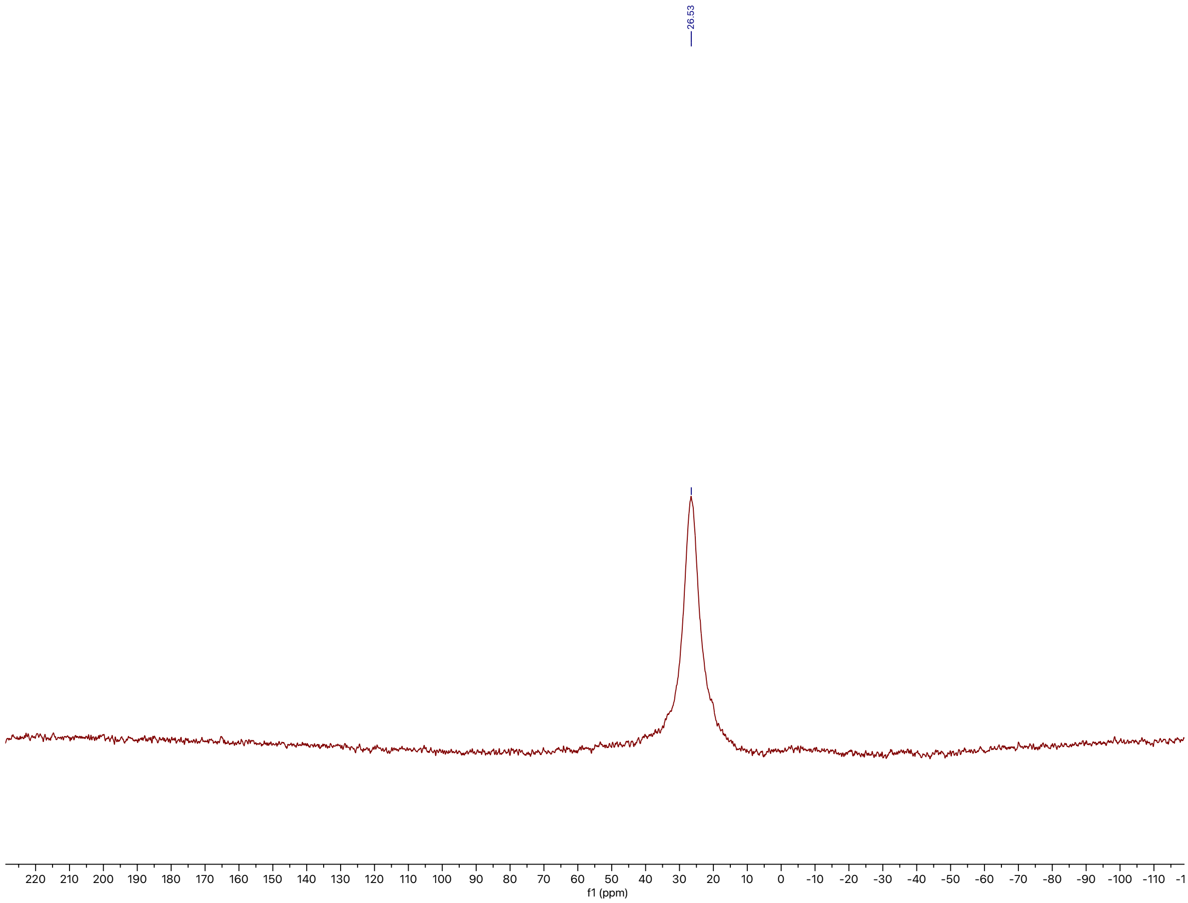

^{1}H-NMR (300 MHz, $CDCl_3$) of **7**

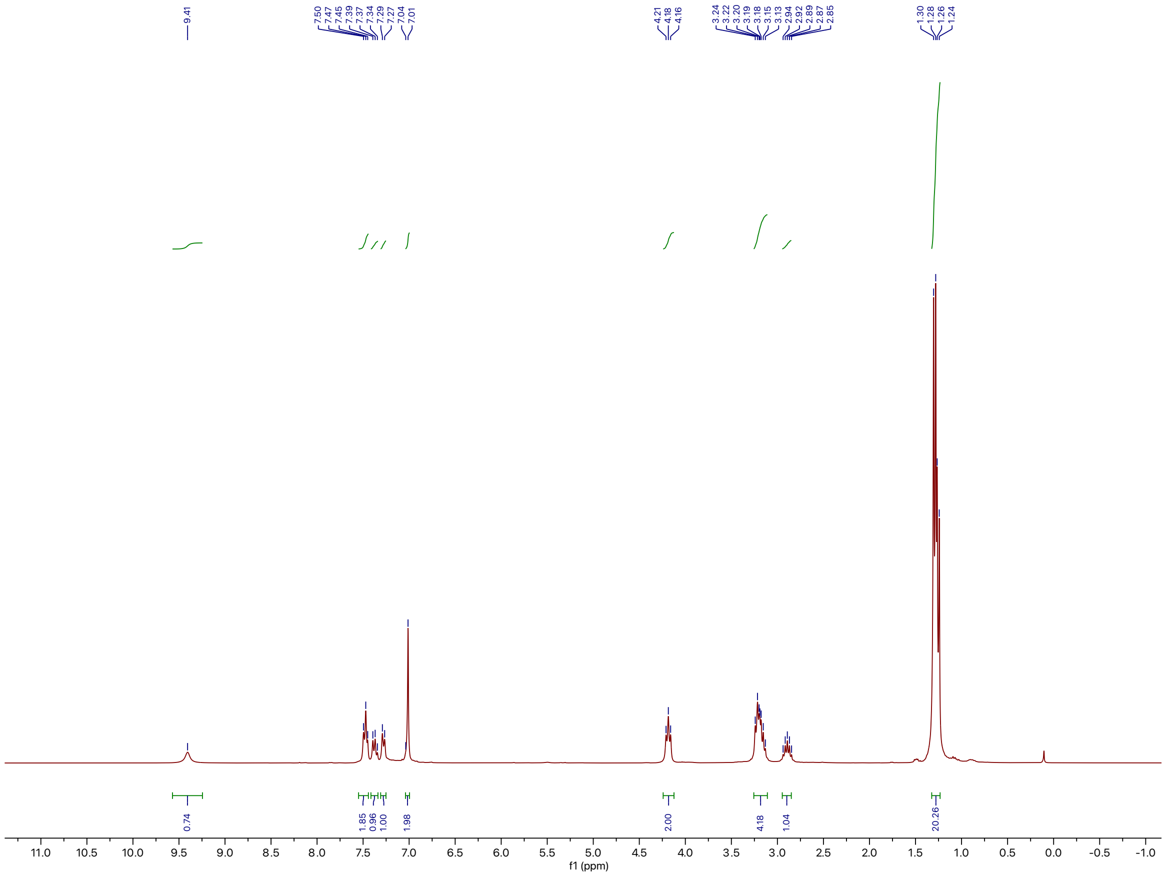

^{13}**C{^{1}H}-NMR** (101 MHz, $CDCl_3$) of **7**

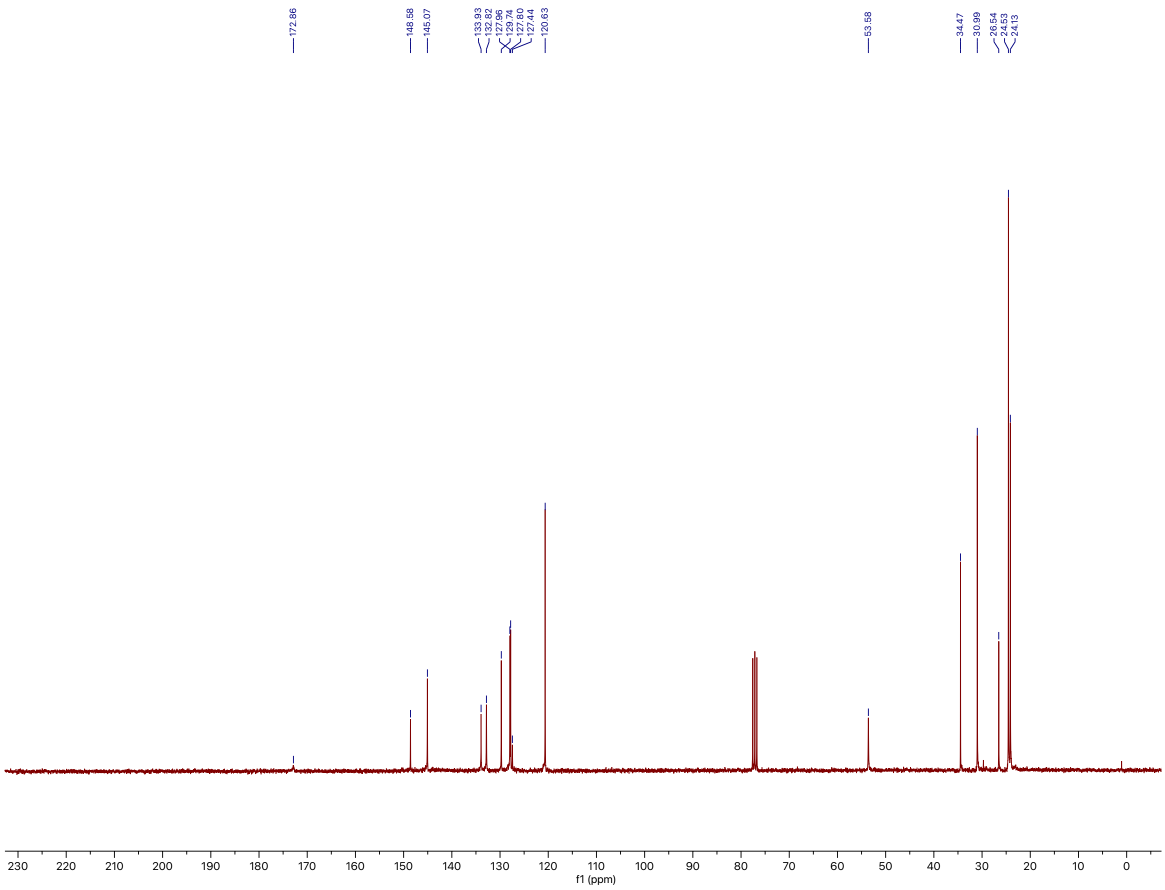

^{1}H-NMR (500 MHz, benzene-d_6) of **7a**

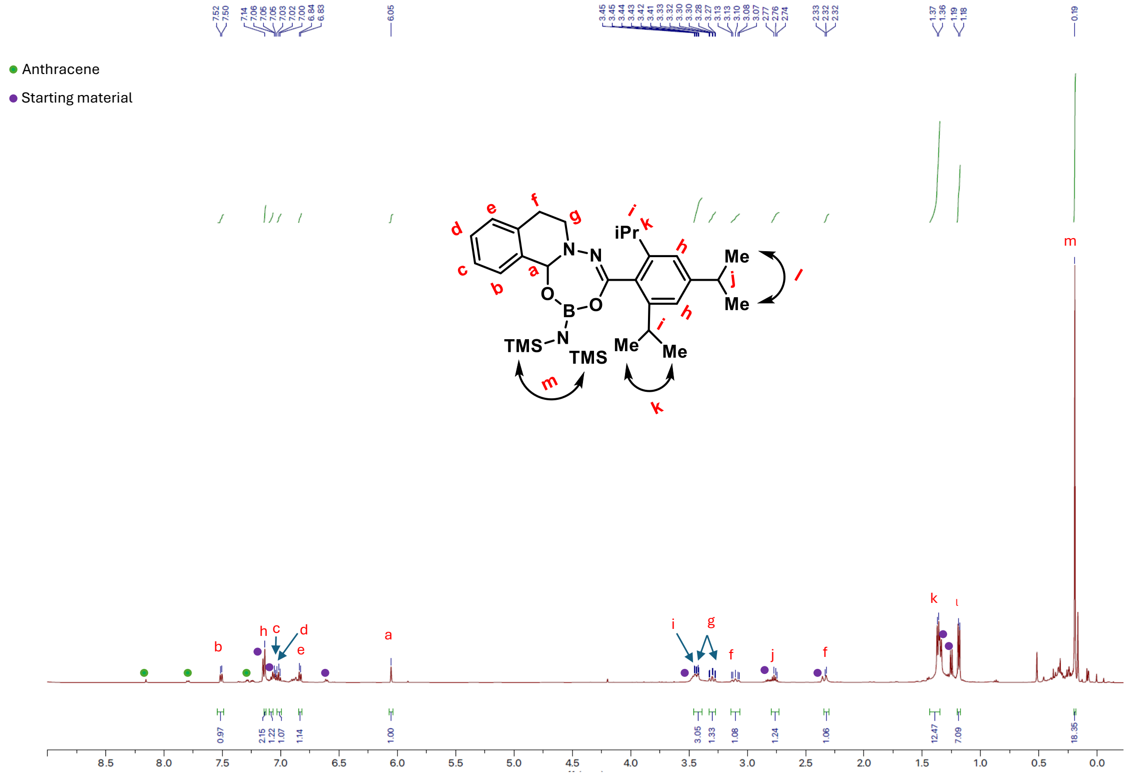

^{13}C{^{1}H}-NMR (126 MHz, $CDCl_3$) of **7a**

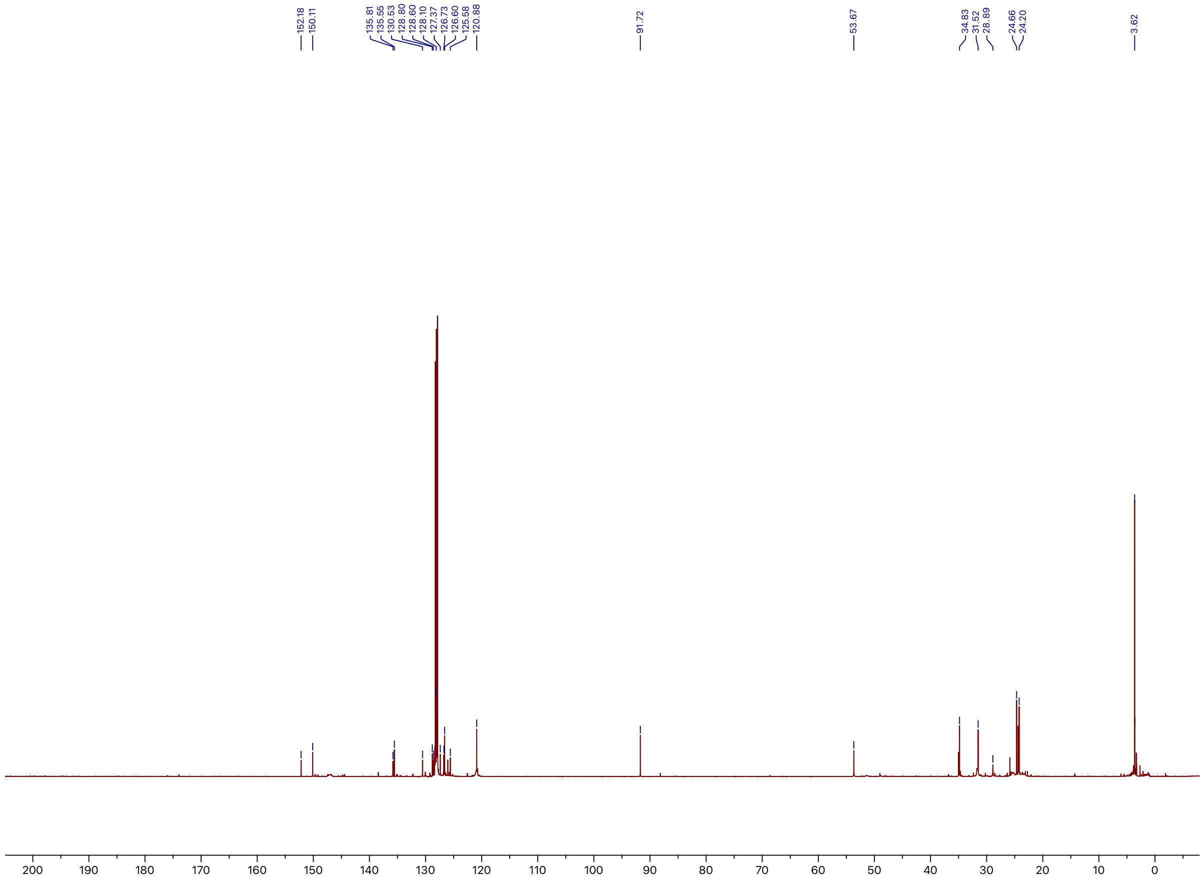

^{11}B-NMR (96 MHz, benzene-d_6) of **7a**

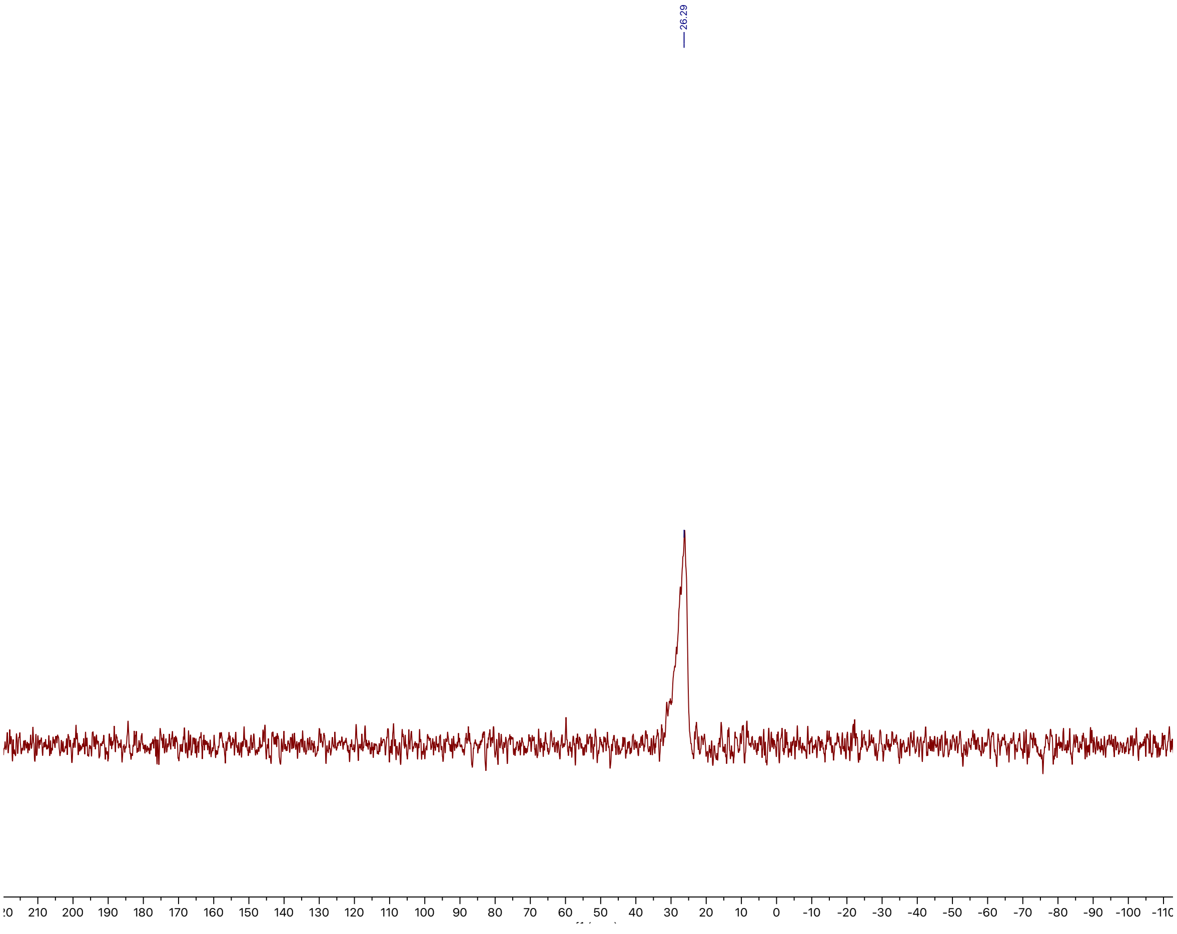

^{1}**H-NMR** (500 MHz, $CDCl_3$) of **9**

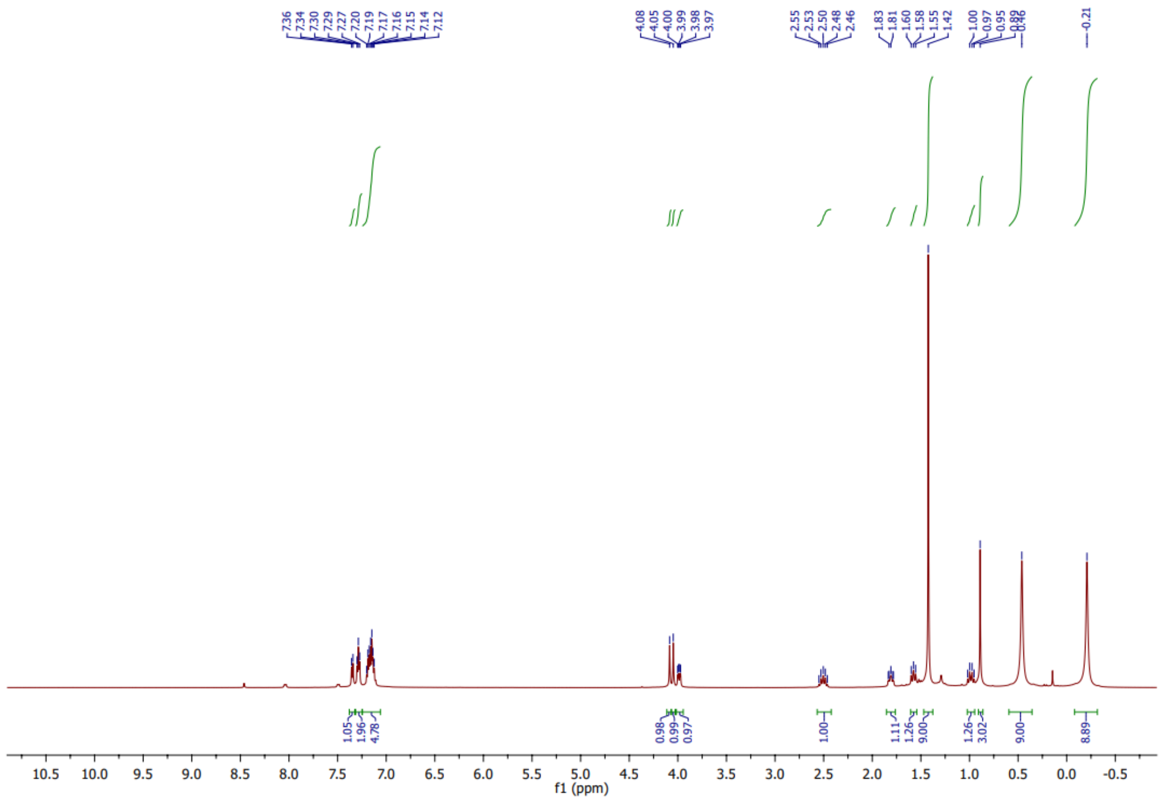

$^{13}C\{^{1}H\}$-NMR (126 MHz, $CDCl_3$) of **9**

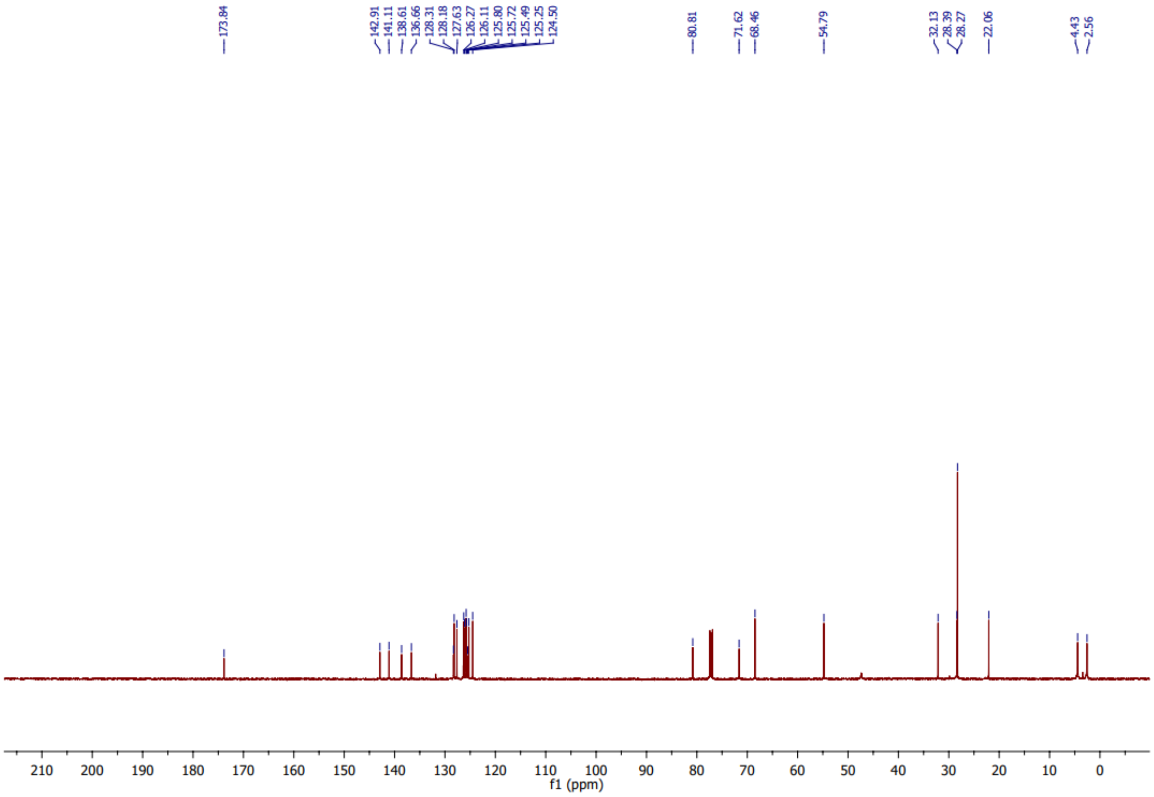

^{11}**B-NMR** (96 MHz, benzene-d_6) of **9**

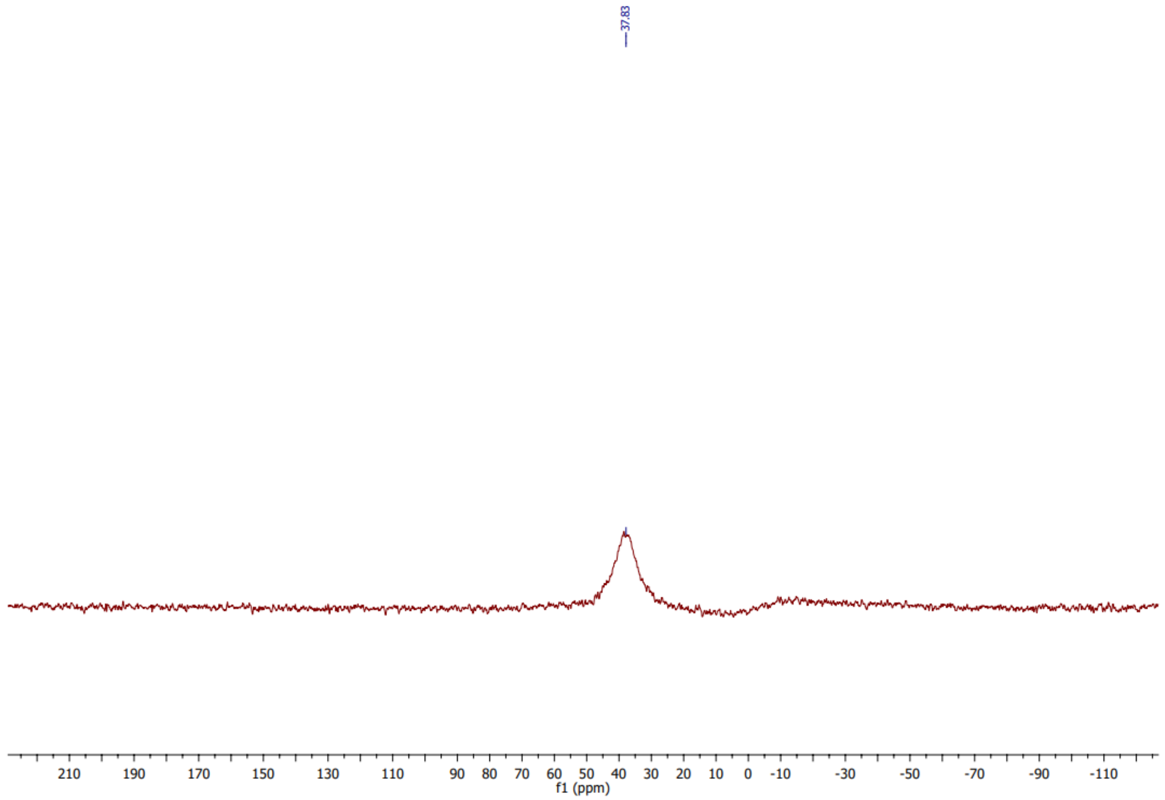

References:

1. Sheldrick, G. M. SHELXT–Integrated space-group and crystal-structure determination. *Acta Crystallographica Section A: Foundations and Advances* **2015**, *71*(1), 3-8.
2. Sheldrick, G. M. Crystal structure refinement with SHELXL. Acta Crystallographica Section C: *Struct. Chem.* **2015**, *71*(1), 3-8.
3. Dolomanov, O. V.; Bourhis, L. J.; Gildea, R. J.; Howard, J. A.; Puschmann, H. OLEX2: a complete structure solution, refinement and analysis program. *Journal of Appl. Crystallogr.* **2009**, *42*(2), 339-341.
4. Ordyszewska, A.; Szynkiewicz, N.; Perzanowski, E.; Chojnacki, J.; Wiśniewska, A.; Grubba, R. Structural and spectroscopic analysis of a new family of monomeric diphosphinoboranes. *J. Chem. Soc., Dalton Trans.* **2019**, *48*(33), 12482-12495.
5. Lis, A. V.; Gostevskii, B. A.; Albanov, A. I.; Yarosh, N. O.; Rakhlin, V. I. Synthesis of volatile bis [bis (trimethylsilyl) amide]-substituted boron derivatives. *Russ. J. Gen. Chem.* **2019**, *87*, 353-356.
6. Ge, F.; Kehr, G.; Daniliuc, C. G.; Erker, G. Borole formation by 1, 1-carboboration. *J. Am. Chem. Soc.* **2014**, *136*(1), 68-71.
7. Bogdanovìc, B.; Janke, N.; Kinzelmann, H. G.; Seevogel, K.; Treber, J. Magnesium Adducts of Substituted Anthracenes–Preparation and Properties. *Chem. Ber.* **1990**, *123*(7), 1529-1536.
8. Robertson, L.; Hartley, R. C. Synthesis of N-arylpyridinium salts bearing a nitrone spin trap as potential mitochondria-targeted antioxidants. *Tetrahedron* **2009**, *65*(27), 5284-5292.
9. Gaussian 16, Revision C.01, M. J. Frisch, G. W. Trucks, H. B. Schlegel, G. E. Scuseria, M. A. Robb, J. R. Cheeseman, G. Scalmani, V. Barone, G. A. Petersson, H. Nakatsuji, X. Li, M. Caricato, A. V. Marenich, J. Bloino, B. G. Janesko, R. Gomperts, B. Mennucci, H. P. Hratchian, J. V. Ortiz, A. F. Izmaylov, J. L. Sonnenberg, D. Williams-Young, F. Ding, F. Lipparini, F. Egidi, J. Goings, B. Peng, A. Petrone, T. Henderson, D. Ranasinghe, V. G. Zakrzewski, J. Gao, N. Rega, G. Zheng, W. Liang, M. Hada, M. Ehara, K. Toyota, R. Fukuda, J. Hasegawa, M. Ishida,

T. Nakajima, Y. Honda, O. Kitao, H. Nakai, T. Vreven, K. Throssell, J. A. Montgomery, Jr., J. E. Peralta, F. Ogliaro, M. J. Bearpark, J. J. Heyd, E. N. Brothers, K. N. Kudin, V. N. Staroverov, T. A. Keith, R. Kobayashi, J. Normand, K. Raghavachari, A. P. Rendell, J. C. Burant, S. S. Iyengar, J. Tomasi, M. Cossi, J. M. Millam, M. Klene, C. Adamo, R. Cammi, J. W. Ochterski, R. L. Martin, K. Morokuma, O. Farkas, J. B. Foresman, and D. J. Fox, Gaussian, Inc., Wallingford CT, 2016.

10. Neese, F.; Wennmohs, F.; Becker, U.; Riplinger, C. The ORCA quantum chemistry program package. *J. Chem. Phys.* **2020**, *152*, 224108.
11. Neese, F. Software update: The ORCA program system - Version 5.0. Wiley Interdiscip. Rev.: *Comput. Mol. Sci.* **2022**, *12*, e1606.
12. Adamo, C.; Barone, V. Toward reliable density functional methods without adjustable parameters: The PBE0 model. *J. Chem. Phys.* **1999**, *110*, 6158-6170.
13. Perdew, J. P.; Burke, K.; Ernzerhof, M. Generalized Gradient Approximation Made Simple. *Phys. Rev. Lett.* **1996**, *77*, 3865-3868.
14. Perdew, J. P.; Burke, K.; Ernzerhof, M. Generalized gradient approximation made simple [Phys. Rev. Lett. 77, 3865 (1996)]. *Phys. Rev. Lett.* **1997**, *78*, 1396.
15. Grimme, S.; Antony, J.; Ehrlich, S.; Kreig, H. A consistent and accurate ab initio parametrization of density functional dispersion correction (DFT-D) for the 94 elements H-Pu. *J. Chem. Phys.* **2010**, *132*, 154104.
16. Grimme, S.; Ehrlich, S.; Goerigk, L. Effect of the damping function in dispersion corrected density functional theory. *J. Comput. Chem.* **2011**, *32*, 1456-1465.
17. Rappoport, D.; Furche, F. Property-optimized Gaussian basis sets for molecular response calculations. *J. Chem. Phys.* **2010**, *133*, 134105.
18. Weigend, F.; Ahlrichs, R. Balanced basis sets of split valence, triple zeta valence and quadruple zeta valence quality for H to Rn: Design and assessment of accuracy. *Phys. Chem. Chem. Phys.* **2005**, *7*, 3297-3305.
19. Weigend, F. Accurate Coulomb-fitting basis sets for H to Rn. *Phys. Chem. Chem. Phys.* **2006**, *8*, 1057-1065.

20. Hellweg, A.; Hättig, C.; Höfener, S.; Klopper, W. Optimized accurate auxiliary basis sets for RI-MP2 and RI-CC2 calculations for the atoms Rb to Rn. *Theor. Chem. Acc.* **2007**, *117*, 587-597.

21. Hellweg, A.; Rappoport, D. Development of new auxiliary basis functions of the Karlsruhe segmented contracted basis sets including diffuse basis functions (def2-SVPD, def2-TZVPPD, and def2-QVPPD) for RI-MP2 and RI-CC calculations. *Phys. Chem. Chem. Phys.* **2015**, *17*, 1010-1017.

22. York, D. M.; Karplus, M. A Smooth Solvation Potential Based on the Conductor-Like Screening Model. *J. Phys. Chem. A* **1999**, *103*, 11060-11079.

23. Barone, V.; Cossi, M. Quantum Calculation of Molecular Energies and Energy Gradients in Solution by a Conductor Solvent Model. *J. Phys. Chem. A* **1998**, *102*, 1995-2001.

24. Ásgeirsson, V.; Birgisson, B. O.; Bjornsson, R.; Becker, U.; Neese, F.; Riplinger, C.; Jónsson, H. Nudged Elastic Band Method for Molecular Reactions Using Energy-Weighted Springs Combined with Eigenvector Following. *J. Chem. Theory Comput.* **2021**, *17*, 4929-4945.

25. Harvey, J. N.; Aschi, M.; Schwartz, H.; Koch, H. The singlet and triplet states of phenyl cation. A hybrid approach for locating minimum energy crossing points between non-interacting potential energy surfaces. *Theor. Chem. Acc.* **1998**, *99*, 95-99.

26. Bannwarth, C.; Ehlert, S.; Grimme, S. GFN2-xTB-An accurate and broadly parametrized self-consistent tight-binding quantum chemical method with multipole electrostatics and density-dependent dispersion contributions. *J. Chem. Theory Comput.* **2019**, *15*(3), 1652-1671.

27. Bannwarth, C.; Caldeweyher, E.; Ehlert, S.; Hansen, A.; Pracht, P.; Seibert, J.; Spicher, S.; Grimme, S. Extended tight-binding quantum chemistry methods. *Wiley Interdiscip. Rev. Comput. Mol. Sci.* **2021**, *11*(2), e1493.

28. Glendening, E. D.; Landis, C. R.; Weinhold, F. Natural bond orbital methods. Wiley Interdiscip. *Rev.: Comput. Mol. Sci.* **2012**, *2*, 1-42.

29. Glendening, E. D.; Landis, C. R.; Weinhold, F. NBO 7.0: New vistas in localized and delocalized chemical bonding theory. *J. Comput. Chem.* **2019**, *40*, 2234-2241.
30. Wiberg, K. B. Application of the pople-santry-segal CNDO method to the cyclopropylcarbinyl and cyclobutyl cation and to bicyclobutane. *Tetrahedron* **1968**, *24*, 1083-1096.
31. Glendening, E. D.; Weinhold, F. Natural resonance theory: I. General formalism. *J. Comput. Chem.* **1998**, *19*, 593-609.
32. Glendening, E. D.; Weinhold, F. Natural resonance theory: II. Natural bond order and valency. *J. Comput. Chem.* **1998**, *19*, 610-627.
33. Glendening, E. D.; Weinhold, F. Natural resonance theory: III. Chemical applications. *J. Comput. Chem.* **1998**, *19*, 628-646.
34. Glendening, E. D.; Landis, C. R.; Weinhold, F. Resonance Theory Reboot. *J. Am. Chem. Soc.* **2019**, *141*, 4156-4166.
35. Glendening, E. D.; Wright, S. J.; Weinhold, F. Efficient optimization of natural resonance theory weightings and bond orders by gram-based convex programming. *J. Comput. Chem.* **2019**, *40*, 2028-2035.
36. Weinhold, F.; Glendening, E. D. Natural resonance-theoretic conceptions of extreme electronic delocalization in soft materials. *Phys. Chem. Chem. Phys.* **2024**, *26*, 2815-2822.
37. Cramer, C. J., *Essentials of Computational Chemistry Theories and Models*. 2nd ed.; John Wiley & Sons: Chichester, UK, 2007.
38. Besora, M.; Vidossich, P.; Lledós, A.; Ujaque, G.; Maseras, F. Calculation of Reaction Free Energies in Solution: A Comparison of Current Approaches. *J. Phys. Chem. A* **2018**, *122*, 1392-1399.
39. Bernardi, F.; Andrea, B.; McDouall, J. J. W.; Robb, M. A.; Schlegel, H. B. MCSCF gradient calculation of transition structures in organic reactions. *Faraday Symp. Chem. Soc.* **1984**, *19*, 137-147.
40. Eade, R. H. A.; Robb, M. A. Direct minimization in MC SCF theory. The quasi-newton method. *Chem. Phys. Lett.* **1981**, *83*, 362-368.

41. Frisch, M. J.; Ragazos, I. N.; Robb, M. A.; Schlegel, H. B. An evaluation of three direct MC-SCF procedures. *Chem. Phys. Lett.* **1992**, *189*, 524-528.
42. Hegarty, D.; Robb, M. A. Application of unitary group methods to configuration interaction calculations. *Mol. Phys.* **1979**, *38*, 1795-1812.
43. Schlegel, H. B.; Robb, M. A. MC SCF gradient optimization of the $H_2CO \rightarrow H_2 + CO$ transition structure. *Chem. Phys. Lett.* **1982**, *93*, 43-46.
44. Schmidt, M. W.; Gordon, M. S. The Construction and Interpretation of MCSCF Wavefunctions. *Ann. Rev. Phys. Chem.* **1998**, *49*, 233-266.
45. Yamamoto, N.; Vreven, T.; Robb, M. A.; Frisch, M. J.; Schlegel, H. B. A direct derivative MC-SCF procedure. *Chem. Phys. Lett.* **1996**, *250*, 373-378.
46. McDouall, J. J. W.; Peasley, K.; Robb, M. A. A simple MC SCF perturbation theory: Orthogonal valence bond Møller-Plesset 2 (OVB MP2). *Chem. Phys. Lett.* **1988**, *148*, 183-189.
47. Grimme, S.; Hansen, A. A Practicable Real-Space Measure and Visualization of Static Electron-Correlation Effects. *Angew. Chem., Int. Ed.* **2015**, *54*, 12308-12313.
48. Bauer, C. A.; Hansen, A.; Grimme, S. The Fractional Occupation Number Weighted Density as a Versatile Analysis Tool for Molecules with a Complicated Electronic Structure. *Chem. Eur. J.* **2017**, *23*, 6150-6164.
